# Sonochemistry and Biocatalysis: Two-Step Green Asymmetric Synthesis of Optically Active Dialkyl(4-(hydroxyalkyl)phenyl)phosphates

**DOI:** 10.1021/acsomega.5c02774

**Published:** 2025-06-30

**Authors:** Lucas Emanuel Beluzzo Iarocz, Marcela Belen Alvarez, Amanda Goldbeck Gerbaudo, Eder João Lenardão, Gelson Perin, Márcio Santos Silva

**Affiliations:** Laboratório de Síntese Orgânica Limpa (LASOL), Centro de Ciências Químicas, Farmacêuticas e de Alimentos (CCQFA), 37902 Universidade Federal de Pelotas (UFPel), P.O. Box 354, 96010-900 Pelotas, RS, Brazil

## Abstract

A green and practical synthetic route for obtaining chiral *O*,*O*-dialkyl-*O*-phenylphosphonate compounds is described here. The two-step synthetic strategy combines sonochemistry with an enzymatic asymmetric reduction. In the first step, aromatic hydroxyketones react with dialkyl *H*-phosphonates employing diphenyl ditelluride as an organocatalyst under ultrasound irradiation for 2 h at 25 °C. The ketophosphonate intermediates were obtained in satisfactory yields (70–97%). In the second step, bioreduction was performed employing *Daucus carota* bits in water at 25 °C for 72 h. The chiral *O*,*O*-dialkyl-*O*-phenylphosphonates were formed in good to excellent yields (50–98%) with satisfactory enantiomeric excesses (up to 99%). This eco-friendly and metal-free synthetic route can also be performed using a two-step sequential synthesis protocol (telescoping approach), reducing waste, cost, and time. The enantiomeric excess of the products was determined by a ^31^P NMR chiral discrimination protocol based on the racemic hydroxyphosphonates using a simple and rapid procedure.

## Graphical abstract

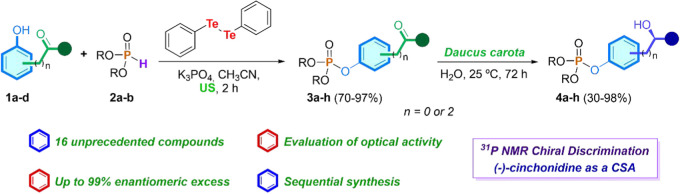

## 1 Introduction

Chiral organophosphorus compounds are known for their consolidate applications in the agrochemical^1,2^ (e.g., herbicides and insecticides) and pharmaceutical^3–6^ (e.g., anti-infectives and oncological drugs) fields. In addition to these applications, chiral phosphorus ligands play a significant role in the synthesis of optically active commercial compounds because of the wide variety of ligands available for organic and metal-based catalysis.^7–11^

The synthesis of chiral compounds remains a challenging task in synthetic chemistry, often requiring elaborate conditions to control the stereocenter.^1–6^ In this context, chiral scaffolds play a crucial role in preparing biologically active chiral organophosphorus compounds, such as hydroxyphosphonates, which are significant intermediates or building blocks to obtain chiral bioactive chemicals (Figure 1). Considering the importance of chiral hydroxyphosphonates, controlling the stereochemistry in synthetic methodologies to prepare them remains a challenging task.

**Figure 1:**
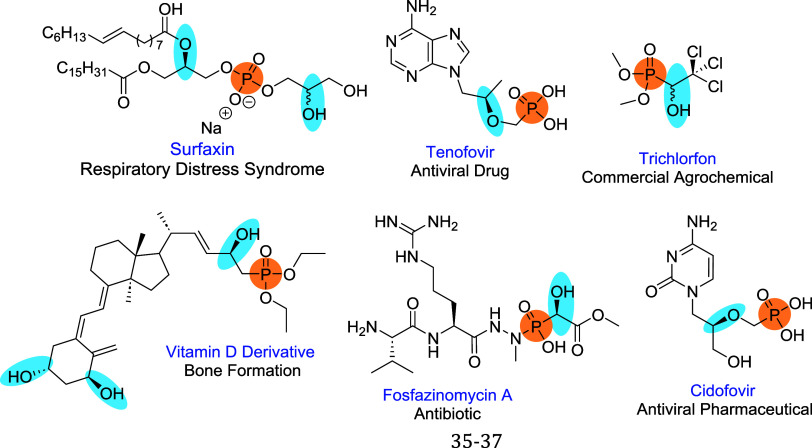
Examples of chiral bioactive chemicals containing organophosphorus and alcohol moieties.

Various synthetic protocols evolved in the last few decades to obtain chiral alcohols, such as the asymmetric catalysis^12–15^ and biocatalysis.^16–19^ To obtain chiral hydroxyphosphonates, routinely, the chiral alcohol substrate is previously prepared and then the phosphonate group is added. For instance, the antiretroviral drug Tenofovir (Figure 1) was prepared using a biocatalytic approach (lipases and *E. coli*/ADH) to obtain the chiral alcohol intermediate, which was subjected to the addition of the phosphonate group.^20^ More recently, Qin and colleagues described the direct synthesis of chiral hydroxyphosphonates via a cascade protocol, which combines photo-oxidation to obtain the carbonyl organic function with a chemoenzymatic reduction to the chiral alcohol, using a KRED mutant enzyme.^21^ By this one-pot protocol, yields up to 92% and enantiomeric excesses up to 99% were obtained. Nonetheless, the reaction scope was limited to diethyl *H*-phosphonate as the organophosphorus source (Scheme 1). Considering that the search for green synthetic protocols is a pivotal aspect in the chemical industry, especially in the synthesis of chiral compounds, and that chiral alcohols are essential starting materials to prepare bioactive organophosphorus chemicals,^22^ herein, we describe a greener, two-step synthetic methodology to prepare *O*,*O*-dialkyl-*O*-phenylphosphonates (Scheme 1).

**Scheme 1:**
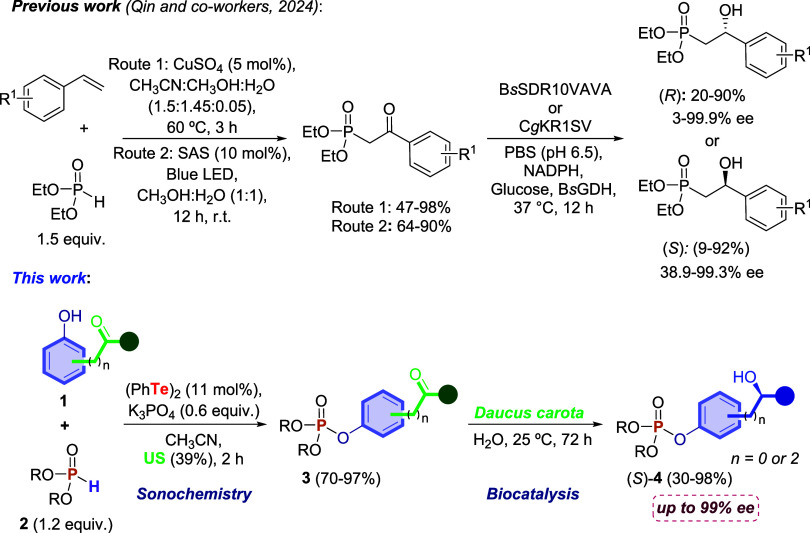
Protocols for the Synthesis of Chiral Hydroxyphosphonates

The two-step synthetic strategy combines sonochemistry and bioreduction. In the first step, ultrasound (US) irradiation is used along with an organocatalyst to add the phosphonate group to aromatic hydroxyketones. The use of US as an alternative energy source has brought several improvements to organic synthesis, such as higher yields, shorter reaction times, and reduced waste.^23^ The ultrasound energy source can be combined with several types of reactions, including organocatalytic, organometallic, organochalcogen, and multicomponent reactions.^23–26^ After the aromatic ketones containing the organophosphorus moiety are synthesized, the next step of our strategy is the enantioselective bioreduction of the prochiral ketones by carrot roots (*Daucus carota*). The effectiveness of carrot roots in the bioreduction of prochiral ketones has been demonstrated using several starting materials.^27–33^ Additionally, carrot roots have demonstrated impressive results in the bioreduction of ketones with low levels of asymmetry,^34^ as well as in the synthesis of bioactive chemicals.^35–37^

Considering the current strategies for the asymmetric reduction of ketones by biocatalysts^38^ and the synthetic versatility of the alcohol function,^12–19^ the enantioselective reduction of ketones containing multiple functional groups is crucial to producing industrially important chemicals. Thus, this synthetic route starts with the reaction between aromatic hydroxyketone derivatives **1** and dialkyl *H*-phosphonates **2** promoted by diphenyl ditelluride (11 mol %) as an organocatalyst under US irradiation (amplitude of 39%) to obtain the ketophosphonate intermediates **3**.

Afterward, the enantioselective bioreduction reaction step of the prochiral ketophosphonates using pieces (7.5 g) of *D. carota* in water (30.0 mL) was carried out. In addition to the synthetic methodologies (Scheme 1), a method was developed to determine the enantiomeric excesses (ee) by phosphorus-31 nuclear magnetic resonance (^31^P NMR) spectroscopy employing (−)-cinchonidine alkaloid as a cheaper and readily available chiral solvating agent (CSA) in a simple and rapid procedure. To develop the ^31^P NMR protocol, racemic *O*,*O*-diethyl-*O*-phenylphosphonate *rac*-**4a** was employed.

## 2 Results and Discussion

At the beginning, the focus was to prepare the new ketophosphonate derivatives **3**, which were used in a recent synthetic protocol developed by our group.^39^ This methodology is based on a dehydrogenative phosphorylation reaction employing diphenyl ditelluride as an organocatalyst and ultrasound as an alternative energy source. The choice of this route was due to the greening aspects of these experimental conditions since neither metal nor oxidant agents are used, while the reaction time is reduced compared to conventional heating. It is important to mention that diaryl ditellurides are reactive toward *H*-phosphonate compounds^40–42^ and can be easily removed from the reaction medium. Additionally, (PhTe)_2_ is an easy-to-handle, nonhygroscopic solid, unlike many metal salts, and exhibits good solubility in most organic solvents.

As shown in Scheme 2, the first reaction involved 4-hydroxyacetophenone **1a** and diethyl *H*-phosphonate **2a**, affording diethyl­(4-acetylphenyl) phosphonate **3a** in 86% yield. On a 4 mmol scale, there was a decrease in yield, and product **3a** was obtained in 63% yield. Although the yield decreased, the reaction conditions remained unchanged on the gram scale.^43^ From this perspective, when the reaction was carried out using conventional heating (oil bath at 100 °C) for 72 h, compound **3a** was obtained in only 43% yield. Changes in the experimental conditions did not lead to an increase in the yield of product **3a**. Eight functionalized ketophosphonate derivatives **3** were prepared in good to excellent yields (70–97%), which were used to evaluate the efficiency of enantioselective bioreduction by *D. carota*. *H*-Phosphonates **2** containing *O*-phenyl and *O*-benzyl groups attached to the phosphorus atom failed to provide respective products **3i** and **3j**. The same lack of reactivity was observed with 2-hydroxyacetophenone **1e**, and compound **3k** could not be obtained under our conditions.

**Scheme 2:**
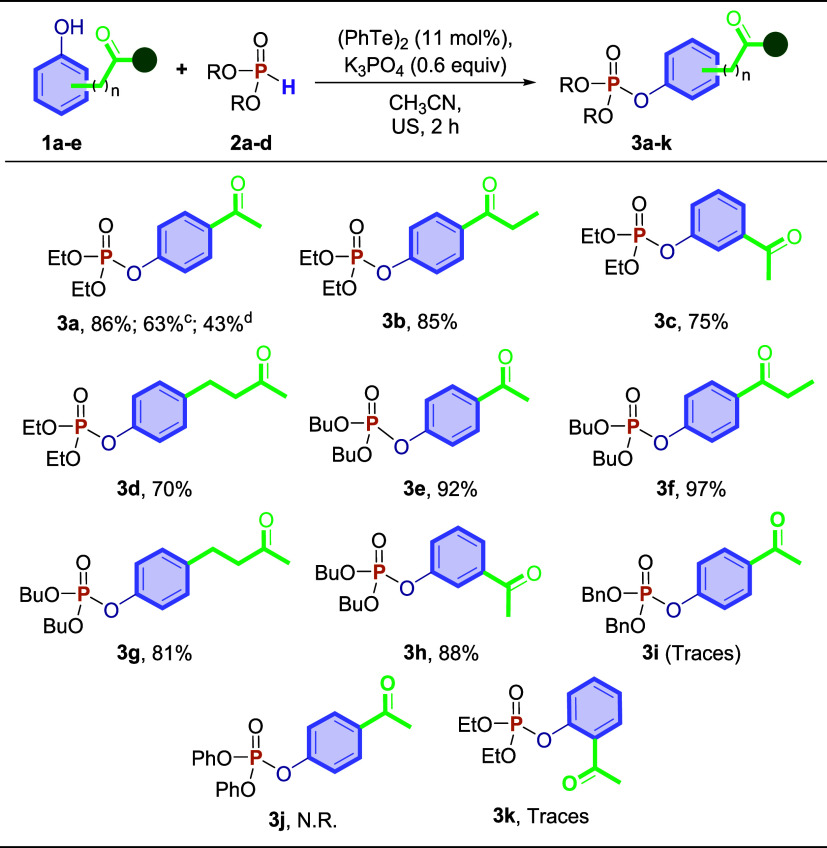
Reaction Scope in the Synthesis of *O*,*O*-dialkyl/aryl-*O*-phenylphosphonate Derivatives **3a**–**k** ^c^,^d^

c Reaction on the 4 mmol scale.
d Reaction under conventional heating (oil bath at 100 °C) for 72 h.
a Conditions: **1** (2.5 mmol), **2** (3.0 mmol), diphenyl ditelluride (11 mol %), K_3_PO_4_ (1.5 mmol) in CH_3_CN (5.0 mL). The reactions were sonicated with a US probe (39% of amplitude) for 2 h. N.R. = no reaction.
b Yields after column chromatography.

With starting materials **3** in hand, we turned our attention to preparing chiral alcohols. Because our focus is also to develop a simple and rapid NMR protocol to carry out chiral discrimination, initially, we prepared the *rac*-*O*,*O*-dialkyl-*O*-phenylphosphonates **4** for a proper chiral recognition evaluation.

Thus, diethyl­(4-acetylphenyl)­phosphonate **3a** was used as a model substrate to optimize the amount of reducing agent (NaBH_4_) and the better solvent to synthesize *O*,*O*-diethyl-*O*-phenylphosphonate *rac*-**4a**. According to Table S1, 1.2 equiv of NaBH_4_ and 2.0 mL of a mixture of EtOH:H_2_O (9:1) afforded the best yield (99%) of *rac*-diethyl­(4-(1-hydroxyethyl)­phenyl)­phosphonate **4a**. After obtaining the optimized experimental conditions, the synthetic methodology was extended to other ketophosphonates **3**, as shown in Scheme 3. The expected *rac*-*O*,*O*-dialkyl-*O*-phenylphosphonates *rac*-**4** were obtained in good to excellent yields (85–99%) after 10–90 min of reaction.

**Scheme 3:**
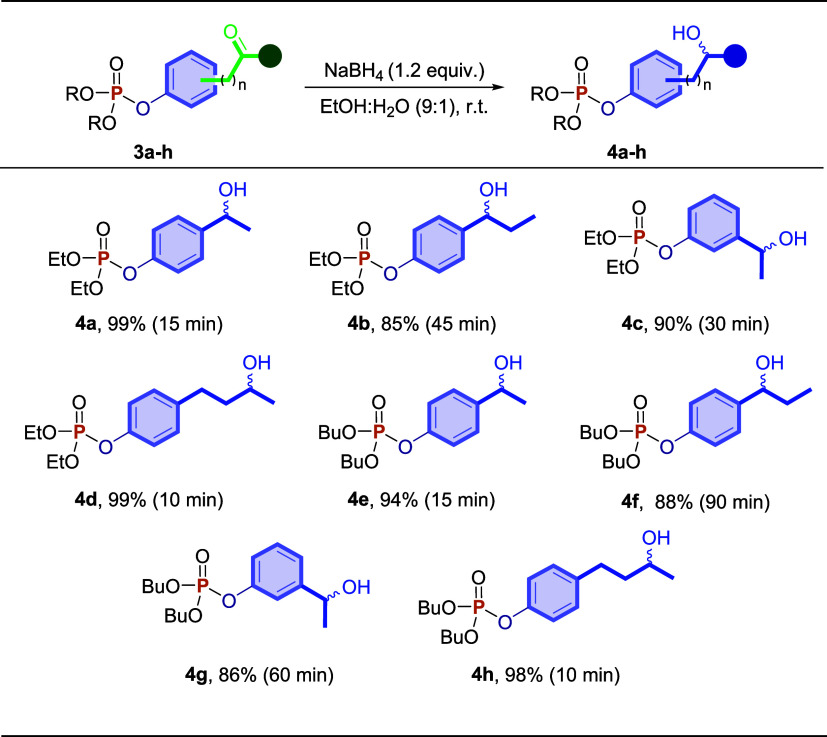
Reaction Scope of the Synthesis of *rac*-*O*,*O*-dialkyl-*O*-phenylphosphonates **4a**–**h** ^a^,^b^

a Conditions: **3** (0.5 mmol) and NaBH_4_ (0.6 mmol) in 2.0 mL of EtOH:H_2_O (9:1) as the solvent.
b Yields are for isolated products, after column chromatography.

The analysis of enantiopurity is an essential step in the development of drugs and other chemicals, as all living organisms in nature are chiral-responsive.^44^ To perform the chiral discrimination of a compound by NMR experiments, a nonequivalent diastereomeric mixture must be produced. Based on this demand, a study of distinct chiral solvating agents (CSAs) was carried out in the presence of *rac*-**4a** (Scheme S1), as the use of CSAs involves a simple, practical, and rapid procedure.^45^ Additionally, to examine relevant differences in the NMR chemical shifts (Δδ^R/S^), phosphorus-31 (^31^P) nuclide was used instead of proton NMR, as it has a high natural abundance of the spin-1/2 nucleus, a wider chemical shift dispersion, no signal overlap, and a magnetogyric ratio of 40.5%.^46–48^

To evaluate the performance of CSA to assess enantiopurity, an equimolar mixture of CSA and *rac*-diethyl­(4-(1-hydroxyethyl)­phenyl)­phosphonate **4a** (0.05 mmol) was dissolved in 700 μL of CDCl_3_ (Scheme S1). In this study, only CSA-5 ((+)-BINOL) and CSA-6 ((−)-cinchonidine) afforded a nonequivalent diastereomeric mixture in the ^31^P­{^1^H} NMR experiment (Figures S52 and S82, respectively). No chiral discrimination was detected when DMSO-*d*_6_ was used as a solvent. Additionally, the ^1^H NMR spectra (Figures S51 and S81) of chiral discrimination by (+)-BINOL and (−)-cinchonidine were checked, but signal overlap hampered an accurate enantiomeric measurement.

According to Figures S52 and S82, the splitting values (Δδ^R/S^) obtained in the ^31^P­{^1^H} NMR experiments for (−)-cinchonidine and (+)-BINOL were 0.0655 ppm (10.61 Hz) and 0.0046 ppm (0.74 Hz), respectively (Scheme S1). The splitting value observed using (+)-BINOL as a chiral solvating agent was not enough to observe both diastereomeric peaks because of the partial overlap of signals. However, for the (−)-cinchonidine chiral solvating agent, the splitting value in the ^31^P­{^1^H} NMR experiment was sufficient for a proper ee measurement, based on the enantiodifferentiation quotient.^49,50^ A test employing 0.1 mmol (2.0 equiv) (−)-cinchonidine in CDCl_3_ did not lead to a significant increase in the efficiency of the chiral discrimination protocol, and the obtained splitting value (Δδ^R/S^) was 0.0711 ppm (11.52 Hz). An attempt was made to conduct an experiment with benzene-*d*_6_ in order to evaluate a higher magnetic nonequivalence for the dynamic diastereoisomeric system due to the anisotropic effect of the benzene ring. Unfortunately, the alkaloid (−)-cinchonidine is insoluble in this solvent (Figure S2).

Based on these results, (−)-cinchonidine (CSA-6) was chosen as the standard CSA to perform the ^31^P NMR chiral discrimination of the other *rac*-*O*,*O*-dialkyl-*O*-phenylphosphonates **4**. Thus, the ^31^P­{^1^H} NMR experiments established the chiral discrimination of the *rac*-hydroxyphosphonates **4** with a Δδ^R/S^ range between 0.0192 and 0.0725 ppm. Unfortunately, for *rac*-hydroxyphosphonates **4d** and **4h**, in which the asymmetric center is far from the phosphonate group, it was not possible to observe the NMR splitting of the phosphorus-31 signals. For these compounds, chiral discrimination was done by gas chromatography (GC) with a chiral stationary phase (Figures S83 and S84). This fact emphasizes the limitation of NMR chiral discrimination protocols when the main organic function and/or active NMR nuclide is far from the asymmetric center.^49^

Once the NMR chiral discrimination methodology was established, the next step consisted of an optimization study for the bioreduction of ketophosphonate derivatives **3** by *D. carota* to obtain the respective chiral *O*,*O*-dialkyl-*O*-phenylphosphonates (*S*)-**4**. In this way, based on the synthetic procedures used in bioreduction,^25–31^ it was established that the amounts of *D. carota* and water are the main parameters to be optimized in this biocatalytic reaction. When we used 7.5 g of carrot bits and 30 mL of water, product **4a** was formed in 98% yield (Table 1, entry 1).

**Table 1:**
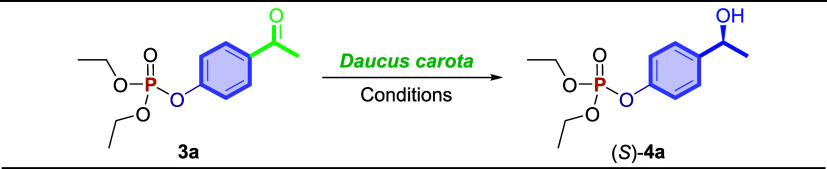
Optimization Results of Bioreduction^a^,^b^

entry	*D. carota* (g)	H_2_O (mL)	yield (%)
**1**	**7.50**	**30**	**98**
2	7.50	15	86
3	3.75	30	90
4	3.75	15	85
5	5.63	22	88
6^c^	7.50	24	88
7^d^	7.50	24	41
8^e^	7.50	24	70
9^f^	7.50	24	43

a Reaction performed using **1a** (0.25 mmol), wild carrot, and water as the solvent. The resulting mixture was stirred for 72 h at 25 °C.

b Isolated yields obtained after column chromatography.

c Six milliliters of EtOH was used as the cosolvent.

d Six milliliters of DMSO was used as the cosolvent.

e Six milliliters of MeCN was used as the cosolvent.

f Six milliliters of DMF was used as the cosolvent.

The decrease in the amount of water (15 mL) provided a yield of 86% (entry 2), and the combination of different amounts of catalyst and water (entries 3–5) allowed us to obtain compound **4a** in excellent yields (85–90%). In addition, distinct cosolvents were tested (DMSO, DMF, MeCN, and EtOH) (entries 6–9), and compound **4a** was obtained in yields ranging from 41 to 88%, which shows that the enzymatic activity decreases in the presence of cosolvents (20% v/v), providing lower yields after 72 h of reaction.

The enzymatic synthesis of enantiomerically enriched *O*,*O*-dialkyl-*O*-phenylphosphonates **4a**–**h** was carried out employing a scale of 0.25 mmol of ketophosphonates **3a**–**h** (Scheme 4). When **3a** was used as the substrate, the chiral product **4a** was obtained in excellent yield (98%) and ee (>99%). When additional methylene was added to product **4b**, both the yield (70%) and ee decreased (86%). Although the yield decreased with the hydroxyalkyl moiety at the *meta*-position (**4c**: 77%) or in the presence of the butyl group on the phosphonate group at the *para*-position of the aromatic ring (**4e**: 74%), the ee remained high (92 and >99%, respectively). It is clear that changes in the model substrate **3a** reduce the effectiveness of *D. carota* bioreduction, as can also be observed in products **4f** and **4g** (Scheme 4). When the asymmetric center is far from the aromatic ring, the product **4d** (*n* = 2) was obtained in 90% yield. Substrate **3h**, another example in which the asymmetric center is far from the aromatic ring, was also efficiently reduced to chiral *O*,*O*-dibutyl-*O*-phenylphosphonate **4h**, providing a yield of 79%.

**Scheme 4:**
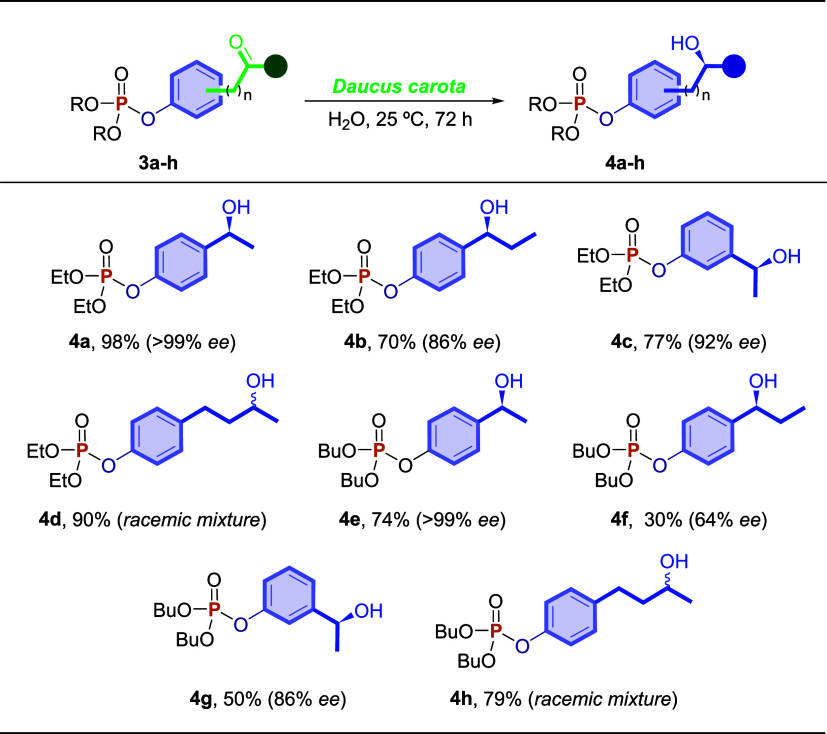
Scope of the Synthesis of Chiral *O*,*O*-dialkyl-*O*-phenylphosphonates **4a**–**h** ^a^,^b^

a Conditions: **3** (0.25 mmol) and *D. carota* (7.5 g) in 30.0 mL of water for 72 h at 25 °C.
b Isolated yields after column chromatography.

It is important to mention that the enantiomeric excesses of products **4d** and **4h** were analyzed by chiral chromatography, as they were not suitable for the ^31^P­{^1^H} NMR experiments in the presence of (−)-cinchonidine. The GC analyses demonstrated that the enantioselectivity of bioreduction was not effective, obtaining products **4d** and **4h** in a racemic form. The determination of ee for the other prepared compounds, however, could not be performed by GC under the standard conditions. Additionally, based on Kazlauskas’s rules,^51^ the decrease in the enantiomeric excesses for products **4d** and **4h**, in our case observed as a racemic mixture, can be attributed to the small difference between the moieties attached to the carbonyl organic function.

Considering the green aspects of this synthetic strategy, a two-step sequential (telescoping) synthesis was also evaluated to reduce time, cost, and waste. For this test, 4-hydroxyacetophenone **1a** and diethyl *H*-phosphonate **2a** (2.0 equiv) were used as the starting materials. Thus, the first step was performed according to the previous standard synthetic protocol (Scheme 1) for 2 h of the reaction. Next, the reaction mixture was transferred, without previous purification or extraction, to an Erlenmeyer flask containing 30.0 mL of water and 7.5 g of *D. carota*. The reaction was allowed to proceed for 72 h, yielding a product with an ee >99%. The remaining ketophosphonate **3a** was recovered in a 61% yield. To increase the reaction yield, a buffer solution of phosphate-buffered saline (PBS, pH = 7.4) was used instead of water due to the presence of a base in the first step. By using this buffer solution in the telescoping approach (Scheme 5: route B), the reaction performance improved, providing the product **4a** in 75% yield (ee > 99%), along with 11% of unreacted intermediate **3a**. The chiral product **4a** was isolated in only 28% yield (Scheme 5: route A) but with a higher ee.

**Scheme 5:**
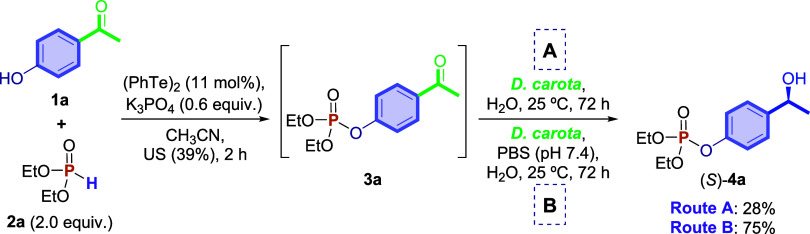
Telescoping Reaction to Obtain the Chiral *O*,*O*-diethyl-*O*-phenylphosphonate **4a**

## 3 Conclusions

Chiral alcohols are ubiquitous in nature and are found in many marketed drugs. We developed a two-step green alternative synthetic route to obtain chiral *O*,*O*-dialkyl-*O*-phenylphosphonates. In the first step, ultrasound irradiation increased yields and shortened reaction times, improving the performance of the dehydrogenative phosphorylation of aromatic hydroxyketones. In the second step, bioreduction using easily available orange carrots proved to be an efficient and practical procedure compared with the traditional enantioselective protocols, which use transition metals and hydrogen gas. Finally, a simple and rapid ^31^P NMR chiral discrimination protocol was developed, facilitating the chiral discrimination processes using the cheap and readily available (−)-cinchonidine alkaloid.

## 4 Experimental Section

### 4.1 General Information

The reactions were monitored by TLC carried out on Merck silica gel (60 F254) by using UV light as a visualization agent and the mixture containing 5% vanillin in 10% H_2_SO_4_ under heating as a developing agent. Column chromatography was performed by using Merck silica gel (pore size 60 Å, 230*–*400 mesh). Mass spectra (MS) were obtained on a gas chromatograph coupled to a Shimadzu GCMS-QP2010 mass spectrometer. Fragments are described by their mass/charge ratio (*m*/*z*) with the relative abundance (%) in parentheses. High-resolution mass spectra (HRMS) were recorded in positive ion mode (APCI) using a Q-TOF spectrometer. High-resolution mass spectra (HRMS) were recorded in positive ion mode (APCI) using a Q-TOF spectrometer and obtained on a HESI Quadrupole-Orbitrap spectrometer (Q-extractive focus, Thermo Scientific) equipped with an APCI source operating in positive ion mode. The samples were solubilized in acetonitrile and analyzed by direct infusion at a constant flow rate. The acquisition parameters were as follows: scanning type, full MS; resolution, 70000; polarity, positive. Ionization conditions (HESI) included sheath gas at 20, auxiliary gas at 10, spray voltage of 2.8 kV, and a capillary temperature of 300 °C. The mass-to-charge ratio (*m*/*z*) data was processed and analyzed using Bruker Daltonics software: Compass Data Analysis and Isotope Pattern. Hydrogen nuclear magnetic resonance (^1^H NMR) and carbon-13 nuclear magnetic resonance (^13^C NMR) spectra were obtained on a Bruker Avance III HD spectrometer at 400 and 100 MHz, respectively. Spectra were recorded in CDCl_3_ solutions. Chemical shifts (δ) are reported in ppm, referenced to tetramethylsilane (TMS) in 0.00 ppm or the residual solvent peak of CHCl_3_ (7.26 ppm) as the internal reference for ^1^H NMR and the solvent peak of CDCl_3_ (77.23 ppm) for ^13^C NMR. Coupling constants (*J*) are reported in hertz. Phosphorus-31 nuclear magnetic resonance spectra (^31^P NMR) were obtained at 162 MHz and referenced to PPh_3_ (−6.00 ppm) employing the substitution method (IUPAC). Abbreviations to denote the multiplicity of a particular signal are s (singlet), d (doublet), t (triplet), q (quartet), quint (quintet), sext (sextet), dd (doublet of doublet), dt (doublet of triplet), td (triplet of doublet), tt (triplet of triplet), qd (quartet of dublet), and m (multiplet). Optical rotations were determined on a JASCO DIP-378 polarimeter (UFABC) (Sodium D line at 589 nm). CHCl_3_ (0.5 mL) was used as the solvent, along with 5.0 mg of compound **4**. Chiral GC-FID analyses were recorded on a Varian 450-GC (UFABC) with a Chirasil-Dex CB-β-cyclodextrin (25 × 0.25 mm^2^) column using H_2_ as the carrier gas. The oven conditions are as follows: 170 °C for 15 min, then ramped at 2 °C/min until 180 °C, followed by holding at 180 °C for 40 min. Compounds **3a**–**h** were prepared according to a published procedure.^39^

### 4.2 Typical Procedure for the Synthesis of Ketophosphonates **3a**–**h**

To a 10.0 mL glass tube were added the appropriate aromatic hydroxyketone **1** (2.5 mmol), dialkyl *H*-phosphonate **2** (3.0 mmol), K_3_PO_4_ (1.5 mmol; 0.032 g), diphenyl ditelluride (11 mol %, 0.113 g), and CH_3_CN (4.0 mL). Afterward, the vial containing the reaction mixture was sonicated with an ultrasound probe (39% amplitude) for 2 h. After completion of the reaction (followed by TLC), the resulting solution was diluted with water (15.0 mL) and the product was extracted with ethyl acetate (3 × 15.0 mL). The organic layer was separated, dried over MgSO_4_, filtered, and concentrated under vacuum. The residue was purified by column chromatography using silica gel and a mixture of hexane/ethyl acetate as an eluent to afford **3a–h**. The organocatalyst can be easily recovered by simple filtration using silica gel.

### 4.3 Typical Procedure for the Synthesis of *rac*-Dialkyl (4-(1-hydroxyalkyl)­phenyl)­phosphonates **4a**–**h**

To a glass tube, 0.50 mmol appropriate ketophosphonate **3** and 2.0 mL of EtOH:H_2_O (9:1) were added. The solution was kept under magnetic stirring at room temperature, and NaBH_4_ (0.60 mmol, 0.023 g) was added to the reaction media. The mixture was stirred for a time varying from 10 to 60 min. Then, 4.0 mL of saturated ammonium chloride (NH_4_Cl) solution was dropped to quench the reaction. The resulting solution was extracted with ethyl acetate (3 × 10.0 mL). The organic layer was separated, dried over MgSO_4_, and concentrated under vacuum. The residue was purified by column chromatography using silica gel and a mixture of hexane/ethyl acetate as an eluent to afford *rac*-**4a–h**.

### 4.4 Typical Procedure for the Synthesis of (*S*)-Dialkyl (4-(1-hydroxyalkyl)­phenyl)­phosphonates **4a**–**h**

In an Erlenmeyer flask, 0.25 mmol appropriate ketophosphonate **3** was added, followed by the addition of 7.5 g of carrot bits and 30.0 mL of distilled water. The system was then immersed in an oil bath and kept under magnetic stirring at 25 °C for 72 h. The consumption of the starting material was monitored by TLC. After 72 h, the reaction mixture was extracted with dichloromethane (3 × 15.0 mL), dried over MgSO_4_, and concentrated under vacuum. The residue was purified by column chromatography using silica gel and a mixture of hexane/ethyl acetate as an eluent to afford (*S*)-**4a–h**.

### 4.5 Typical Procedure for the ^31^P NMR Chiral Discrimination of Racemic and Chiral **4a**–**h**

In an NMR tube, 0.05 mmol respective hydroxyphosphonate **4** was added along with (−)-cinchonidine (1.0 equiv, 0.015 g) in 700 μL of CDCl_3_. Next, the ^31^P­{^1^H} NMR experiments were performed employing the following NMR parameters: 32 scans, 1.25 s of acquisition time, 160 ppm of spectral window, 14 μs of pulse width, and 0.5 s of relaxation delay.

### 4.6 Characterization Data

#### 4.6.1 4-Acetylphenyl diethyl phosphate (**3a**)

Purified by column chromatography (hexane/ethyl acetate = 70:30); Yield: 0.585 g (86%); yellowish oil. ^1^H NMR (CDCl_3_, 400 MHz) δ (ppm) = 7.97 (d, *J* = 8.5 Hz, 2H); 7.31 (d, *J* = 8.7 Hz, 2H); 4.28–4.20 (m, 4H); 2.59 (s, 3H); 1.37 (td, *J* = 7.1 and 0.9 Hz, 6H).^13^C­{^1^H} NMR (CDCl_3_, 100 MHz) δ (ppm) = 196.8; 154.6 (d, *J*_C–P_ = 6.7 Hz); 134.0; 130.4; 120.0 (d, *J*_C–P_ = 5.2 Hz); 65.0 (d, *J*_C–P_ = 6.0 Hz); 26.6; 16.2 (d, *J*_C–P_ = 6.5 Hz). ^31^P­{^1^H} NMR (CDCl_3,_ 162 MHz) δ (ppm) = −12.6. MS (rel. int., %) *m*/*z*: 272 (31.69), 257 (100.0), 229 (42.17), 201 (37.05), 77 (3.33). HRMS (APCI-QTOF) *m*/*z*: [M + H]^+^ Calcd for C_12_H_17_O_5_P: 273.0886; Found: 273.0882.

#### 4.6.2 Diethyl (4-propionylphenyl)­phosphate (**3b**)

Purified by column chromatography (hexane/ethyl acetate = 75:25); yield: 0.606 g (85%); yellow oil. ^1^H NMR (CDCl_3_, 400 MHz) δ (ppm) = 7.98 (d, *J* = 8.5 Hz, 2H); 7.30 (dd, *J* = 8.8 and 0.8 Hz, 2H); 4.28–4.20 (m, 4H); 2.98 (q, *J* = 7.3 Hz, 2H); 1.37 (td, *J* = 7.1 and 1.0 Hz, 6H); 1.22 (t, *J* = 7.2 Hz, 3H). ^13^C­{^1^H} NMR (CDCl_3_, 100 MHz) δ (ppm) = 199.6; 154.4 (d, *J*_C–P_ = 6.8 Hz); 133.9; 130.2; 120.1 (d, *J*_C–P_ = 5.0 Hz); 65.0 (d, *J*_C–P_ = 6.0 Hz); 31.9; 16.2 (d, *J*_C–P_ = 6.5 Hz); 8.3. ^31^P­{^1^H} NMR (CDCl_3,_ 162 MHz) δ (ppm) = −12.5. MS (rel. int., %) *m*/*z*: 286 (7.18), 257 (100.0), 229 (31.81), 201 (31.26), 77 (2.24). HRMS (APCI-QTOF) *m*/*z*: [M + H]^+^ Calcd for C_13_H_19_O_5_P: 287.1043; Found: 287.1039.

#### 4.6.3 3-Acetylphenyl diethyl phosphate (**3c**)

Purified by column chromatography (hexane/ethyl acetate = 70:30); yield: 0.511 g (75%); yellow oil. ^1^H NMR (CDCl_3_, 400 MHz) δ (ppm) = 7.77 (m, 2H); 7.45 (m, 2H); 4.28–4.20 (m, 4H); 2.60 (s, 3H); 1.37 (td, *J* = 7.0 and 1.0 Hz, 6H).^13^C­{^1^H} NMR (CDCl_3_, 100 MHz) δ (ppm) = 197.0; 151.1 (d, *J*_C–P_ = 6.7 Hz); 138.8; 130.0; 124.8 (d, *J*_C–P_ = 4.5 Hz); 119.8 (d, *J*_C–P_ = 5.2 Hz); 64.8 (d, *J* = 6.1 Hz); 26.7; 16.1 (d, *J* = 6.6 Hz). ^31^P­{^1^H} NMR (CDCl_3,_ 162 MHz) δ (ppm) = −12.1. MS (rel. int., %) *m*/*z*: 272 (37.50), 257 (100.0), 229 (81.91), 201 (57.81), 77 (12.02). HRMS (APCI-QTOF) *m*/*z*: [M + H]^+^ Calcd for C_12_H_17_O_5_P: 273.0886; Found: 273.0885.

#### 4.6.4 Diethyl (4-(3-oxobutyl)­phenyl)­phosphate (**3d**)

Purified by column chromatography (hexane/ethyl acetate = 80:20); yield: 0.526 g (70%); yellowish oil. ^1^H NMR (CDCl_3_, 400 MHz) δ (ppm) = 7.16–7.10 (m, 4H); 4.25–4.17 (m, 4H); 2.86 (t, *J* = 7.4 Hz, 2H); 2.73 (t, *J* = 7.6 Hz, 2H); 2.14 (s, 3H); 1.35 (td, *J* = 7.1 and 0.9 Hz, 6H). ^13^C­{^1^H} NMR (CDCl_3_, 100 MHz) δ (ppm) = 207.9; 149.2 (d, *J*_C–P_ = 6.9 Hz); 137.9; 129.7; 120.1 (d, *J*_C–P_ = 4.8 Hz); 64.7 (d, *J*_C–P_ = 6.0 Hz); 45.2; 30.2; 29.1; 16.2 (d, *J*_C–P_ = 6.6 Hz). ^31^P­{^1^H} NMR (CDCl_3,_ 162 MHz) δ (ppm) = −11.8. MS (rel. int., %) *m*/*z*: 300 (13.03), 257 (66.10), 201 (46.61), 77 (20.93), 43 (100.0). HRMS (APCI-QTOF) *m*/*z*: [M + H]^+^ Calcd for C_14_H_21_O_5_P: 301.1199; Found: 301.1200.

#### 4.6.5 4-Acetylphenyl dibutyl phosphate (**3e**)

Purified by column chromatography (hexane/ethyl acetate = 80:20); yield: 0.753 g (92%); yellowish oil. ^1^H NMR (CDCl_3_, 400 MHz) δ (ppm) = 7.94 (d, *J* = 8.5 Hz, 2H); 7.29 (dd, *J* = 8.8 and 0.8 Hz, 2H); 4.17–4.12 (m, 4H); 2.57 (s, 3H); 1.67 (quint, *J* = 6.5 Hz, 4H); 1.38 (sext, *J* = 7.4 Hz, 4H); 0.91 (t, *J* = 7.4 Hz, 6H). ^13^C­{^1^H} NMR (CDCl_3_, 100 MHz) δ (ppm) = 196.9; 154.7 (d, *J*_C–P_ = 6.5 Hz); 134.1; 130.5; 120.1 (d, *J*_C–P_ = 5.3 Hz); 68.7 (d, *J* = 6.3 Hz); 32.3 (d, *J* = 6.7 Hz); 26.7; 18.8; 13.7. ^31^P­{^1^H} NMR (CDCl_3,_ 162 MHz) δ (ppm) = −12.3. MS (rel. int., %) *m*/*z*: 328 (4.07), 313 (20.03), 77 (9.71), 43 (47.61), 41 (100.0). HRMS (APCI-QTOF) *m*/*z*: [M + H]^+^ Calcd for C_16_H_25_O_5_P: 329.1512; Found: 329.1509.

#### 4.6.6 Dibutyl (4-propionylphenyl)­phosphate (**3f**)

Purified by column chromatography (hexane/ethyl acetate = 85:15); yield: 0.832 g (97%); yellow oil. ^1^H NMR (CDCl_3_, 400 MHz) δ (ppm) = 7.90 (d, *J* = 8.7 Hz, 2H); 7.22 (d, *J* = 8.8 Hz, 2H); 4.09 (qd, *J* = 6.9 and 2.3 Hz, 4H); 2.91 (q, *J* = 7.2 Hz, 2H); 1.61 (quint, *J* = 6.7 Hz, 4H); 1.33 (sext, *J* = 7.5 Hz, 4H); 1.15 (t, *J* = 7.2 Hz, 3H); 0.85 (t, *J* = 7.4 Hz, 6H). ^13^C­{^1^H} NMR (CDCl_3_, 100 MHz) δ (ppm) = 196.7; 154.5 (d, *J*_C–P_ = 6.5 Hz); 133.9; 130.2; 120.1 (d, *J*_C–P_ = 5.3 Hz); 68.7 (d, *J*_C–P_ = 6.4 Hz); 32.3 (d, *J* = 6.7 Hz); 31.9; 18.8; 13.7; 8.4. ^31^P­{^1^H} NMR (CDCl_3,_ 162 MHz) δ (ppm) = −12.3. MS (rel. int., %) *m*/*z*: 342 (5.84), 313 (100.0), 257 (32.25), 201 (50.88), 77 (1.82). HRMS (APCI-QTOF) *m*/*z*: [M + H]^+^ Calcd for C_17_H_27_O_5_P: 343.1669; Found: 343.1666.

#### 4.6.7 3-Acetylphenyl dibutyl phosphate (**3g**)

Purified by column chromatography (hexane/ethyl acetate = 80:20); yield: 0.600 g (88%); yellow oil. ^1^H NMR (CDCl_3_, 400 MHz) δ (ppm) = 7.78–7.76 (m, 2H); 7.46–7,44 (m. 2H); 4.17 (qd, *J* = 6.7 and 1.9 Hz, 4H); 2.60 (s, 3H); 1.69 (quint, *J* = 6.7 Hz, 4H); 1.41 (sext, *J* = 7.4 Hz, 4H); 0.93 (t, *J* = 7.4 Hz, 6H). ^13^C­{^1^H} NMR (CDCl_3_, 100 MHz) δ (ppm) = 197.1; 151.2 (d, *J*_C–P_ = 6.8 Hz); 138.9; 130.1; 125.0; 124.8 (d, *J*_C–P_ = 4.6 Hz); 119.9 (d, *J*_C–P_ = 5.3 Hz); 68.6 (d, *J*_C–P_ = 6.3 Hz); 32.3 (d, *J* = 6.7 Hz); 26.8; 18.7; 13.6. ^31^P­{^1^H} NMR (CDCl_3,_ 162 MHz) δ (ppm) = −11.8. MS (rel. int., %) *m*/*z*: 328 (11.24), 313 (34.03), 257 (47.22), 217 (100.0), 77 (9.18). HRMS (APCI-QTOF) *m*/*z*: [M + H]^+^ Calcd for C_16_H_25_O_5_P: 329.1512; Found: 329.1507.

#### 4.6.8 Dibuthyl (4-(3-oxobutyl)­phenyl)­phosphate (**3h**)

Purified by column chromatography (hexane/ethyl acetate = 85:15); yield: 0.722 g (81%); yellowish oil. ^1^H NMR (CDCl_3_, 400 MHz) δ (ppm) = 7.15–7.10 (m, 4H); 4.16–4.11 (m, 4H); 2.86 (t, *J* = 7.5 Hz, 2H); 2.73 (t, *J* = 7.4 Hz, 2H); 2.13 (s, 3H); 1.67 (quint, *J* = 6.7 Hz, 4H); 1.40 (sext, *J* = 7.5 Hz, 4H); 0.92 (t, *J* = 7.4 Hz, 2H). ^13^C­{^1^H} NMR (CDCl_3_, 100 MHz) δ (ppm) = 207.9; 149.2 (d, *J*_C–P_ = 7.0 Hz); 137.8; 129.6; 120.1 (d, *J*_C–P_ = 4.8 Hz); 68.4 (d, *J*_C–P_ = 6.3 Hz); 45.2; 32.3 (d, *J*_C–P_ = 6.9 Hz); 30.2; 29.1; 18.7; 13.7. ^31^P­{^1^H} NMR (CDCl_3,_ 162 MHz) δ (ppm) = −11.6. MS (rel. int., %) *m*/*z*: 356 (5.76), 257 (19.23), 201 (100.0), 77 (11.69), 43 (26.14). HRMS (APCI-QTOF) *m*/*z*: [M + H]^+^ Calcd for C_18_H_29_O_5_P: 357.1825; Found: 357.1825.

#### 4.6.9 Diethyl (4-(1-hydroxyethyl)­phenyl)­phosphate (**4a**)

Purified by column chromatography (hexane/ethyl acetate = 40:60); yield: 0.136 g (99%); yellowish oil. ^1^H NMR (CDCl_3_, 400 MHz) δ (ppm) = 7.32 (d, *J* = 8.6 Hz, 2H); 7.14 (d, *J* = 7.8 Hz, 2H); 4.83 (q, *J* = 6.4 Hz, 1H); 4.20–4.16 (m, 4H); 3.16 (s, 1H); 1.43 (d, *J* = 6.5 Hz, 3H); 1.33 (td, *J* = 7.0 and 0.8 Hz, 6H). ^13^C­{^1^H} NMR (CDCl_3_, 100 MHz) δ (ppm) = 149.7 (d, *J*_C–P_ = 6.8 Hz); 143.1; 126.8; 119.8 (d, *J*_C–P_ = 4.9 Hz); 69.4; 64.7 (d, *J*_C–P_ = 6.0 Hz); 25.4; 16.1 (d, *J*_C–P_ = 6.7 Hz). ^31^P­{^1^H} NMR (CDCl_3,_ 162 MHz) δ (ppm) = −12.1. MS (rel. int., %) *m*/*z*: 274 (15.49), 259 (70.84), 231 (45.12), 203 (100.0), 77 (72.57). HRMS (APCI-QTOF) *m*/*z*: [M + H]^+^ Calcd for C_12_H_17_O_5_P: 273.0897; Found: 273.0902.

#### 4.6.10 Diethyl (4-(1-hydroxypropyl)­phenyl)­phosphate (**4b**)

Purified by column chromatography (hexane/ethyl acetate = 40:60); yield: 0.122 g (85%); yellow oil. ^1^H NMR (CDCl_3_, 400 MHz) δ (ppm) = 7.29 (d, *J* = 8.7 Hz, 2H); 7.16 (dd, *J* = 8.7 and 1.0 Hz, 2H); 4.56 (t, *J* = 6.5 Hz, 1H); 4.23–4.15 (m, 4H); 1.81–1.66 (m, 2H); 1.33 (td, *J* = 7.1 and 1.0 Hz, 6H); 0.88 (t, *J* = 7.4 Hz, 3H). ^13^C­{^1^H} NMR (CDCl_3_, 100 MHz) δ (ppm) = 150.1 (d, *J*_C–P_ = 6.8 Hz); 141.6; 127.5; 120.0 (d, *J*_C–P_ = 4.9 Hz); 75.5; 64.8 (d, *J*_C–P_ = 5.9 Hz); 32.1; 16.2 (d, *J*_C–P_ = 6.6 Hz); 10.2. ^31^P­{^1^H} NMR (CDCl_3,_ 162 MHz) δ (ppm) = −11.9. MS (rel. int., %) *m*/*z*: 288 (2.49), 270 (72.34), 259 (72.96), 116 (100.0), 77 (61.52). HRMS (APCI-QTOF) *m*/*z*: [M + H]^+^ Calcd for C_13_H_21_O_5_P: 289.1199; Found: 289.1199.

#### 4.6.11 Diethyl (3-(1-hydroxypropyl)­phenyl)­phosphate (**4c**)

Purified by column chromatography (hexane/ethyl acetate = 40:60); yield: 0.123 g (90%); yellow oil. ^1^H NMR (CDCl_3_, 400 MHz) δ (ppm) = 7.27 (t, *J* = 7.9 Hz, 1H); 7.22 (s, 1H); 7.15 (d, *J* = 7.6 Hz, 1H); 7.08 (dt, *J* = 8.1 and 1.2 Hz, 1H); 4.83 (q, *J* = 6.5 Hz, 1H); 4.22–4.15 (m, 4H); 3.20 (s, 1H); 1.44 (d, *J* = 6.1 Hz, 3H); 1.33 (tt, *J* = 7.1 and 1.2 Hz, 6H). ^13^C­{^1^H} NMR (CDCl_3_, 100 MHz) δ (ppm) = 150.8 (d, *J*_C–P_ = 6.9 Hz); 148.6; 122.2; 118.6 (d, *J*_C–P_ = 4.6 Hz); 117.2 (d, *J*_C–P_ = 5.1 Hz); 69.6; 64.7 (d, *J* = 6.0 Hz); 25.3; 16.2 (d, *J* = 6.6 Hz). ^31^P­{^1^H} NMR (CDCl_3,_ 162 MHz) δ (ppm) = −12.0. MS (rel. int., %) *m*/*z*: 274 (15.81), 231 (54.71), 203 (100.0), 77 (45.15), 43 (20.45). HRMS (APCI-QTOF) *m*/*z*: [M + H]^+^ Calcd for C_12_H_17_O_5_P: 275.1043; Found: 275.1039;.

#### 4.6.12 Diethyl (4-(3-hydroxybutyl)­phenyl)­phosphate (**4d**)

Purified by column chromatography (hexane/ethyl acetate = 40:60); yield: 0.150 g (99%); yellowish oil. ^1^H NMR (CDCl_3_, 400 MHz) δ (ppm) = 7.10 (q, *J* = 8.4 Hz, 4H); 4.17 (t, *J* = 7.2 Hz, 4H); 3.76 (sext, *J* = 6.0 Hz, 1H); 2.73–2.56 (m, 2H); 1.77–1.62 (m, 2H); 1.31 (t, *J* = 7.0 Hz, 6H); 1.18 (d, *J* = 6.1 Hz, 3H). ^13^C­{^1^H} NMR (CDCl_3_, 100 MHz) δ (ppm) = 148.9 (d, *J*_C–P_ = 7.1 Hz); 139.1; 129.6; 119.9 (d, *J*_C–P_ = 4.8 Hz); 67.3; 64.7 (d, *J*_C–P_ = 6.1 Hz); 40.9; 31.5; 23.7; 16.2 (d, *J*_C–P_ = 6.7 Hz). ^31^P­{^1^H} NMR (CDCl_3,_ 162 MHz) δ (ppm) = −11.8. MS (rel. int., %) *m*/*z*: 301 (1.17), 257 (36.48), 201 (36.14), 77 (20.73), 43 (100.0). HRMS (APCI-QTOF) *m*/*z*: [M + H]^+^ Calcd for C_14_H_23_O_5_P: 302.1277; Found: 302.1233.

#### 4.6.13 Dibutyl (4-(1-hydroxyethyl)­phenyl)­phosphate (**4e**)

Purified by column chromatography (hexane/ethyl acetate = 50:50); yield: 0.155 g (94%); yellow oil. ^1^H NMR (CDCl_3_, 400 MHz) δ (ppm) = 7.34 (d, *J* = 8.7 Hz, 2H); 7.18 (dd, *J* = 8.7 and 1.0 Hz, 2H); 4.89 (q, *J* = 6.4 Hz, 1H); 4.14 (qd, *J* = 6.7 and 2.6 Hz, 4H); 1.67 (quint, *J* = 6.6 Hz, 4H); 1.47 (d, *J* = 6.4 Hz, 3H); 1.40 (sext, *J* = 7.5 Hz, 4H); 0.92 (t, *J* = 7.4 Hz, 6H). ^13^C­{^1^H} NMR (CDCl_3_, 100 MHz) δ (ppm) = 150.2 (d, *J*_C–P_ = 6.7 Hz); 142.7; 120.2 (d, *J*_C–P_ = 4,8 Hz); 70.0; 68.5 (d, *J* = 6.3 Hz); 32.4 (d, *J* = 6.7 Hz); 25.5; 18.8; 13.7. ^31^P­{^1^H} NMR (CDCl_3,_ 162 MHz) δ (ppm) = −11.6. MS (rel. int., %) *m*/*z*: 272 (31.69), 257 (100.0), 229 (42.17), 201 (37.05), 77 (3.33). HRMS (APCI-QTOF) *m*/*z*: [M + H]^+^ Calcd for C_16_H_25_O_5_P: 331.1669; Found: 331.1662.

#### 4.6.14 Dibutyl (4-propionylphenyl)­phosphate (**4f**)

Purified by column chromatography (hexane/ethyl acetate = 50:50); yield: 0.151 g (88%); yellowish oil. ^1^H NMR (CDCl_3_, 400 MHz) δ (ppm) = 7.21 (d, *J* = 8.0 Hz, 2H); 7.08 (d, *J* = 8.3 Hz, 2H); 4.49 (t, *J* = 6.5 Hz, 1H); 4.05 (q, *J* = 6.5 Hz, 4H); 1.76–1.55 (m, 6H); 1.31 (sext, *J* = 7.4 Hz, 4H); 0.86–0.79 (m, 9H). ^13^C­{^1^H} NMR (CDCl_3_, 100 MHz) δ (ppm) = 150.0 (d, *J*_C–P_ = 6.9 Hz); 141.6; 127.4; 119.9 (d, *J*_C–P_ = 4.9 Hz); 75.3; 68.4 (d, *J* = 6.3 Hz); 32.3 (d, *J* = 6.8 Hz); 32.1; 18.7; 13.7; 10.2. ^31^P­{^1^H} NMR (CDCl_3,_ 162 MHz) δ (ppm) = −11.8. MS (rel. int., %) *m*/*z*: 344 (1.50), 315 (52.66), 214 (100.0), 203 (63.79), 77 (35.43). HRMS (APCI-QTOF) *m*/*z*: [M + H]^+^ Calcd for C_17_H_29_O_5_P: 345.1825; Found: 345.1823.

#### 4.6.15 Dibutyl (3-(1-hydroxyethyl)­phenyl)­phosphate (**4g**)

Purified by column chromatography (hexane/ethyl acetate = 50:50); yield: 0.142 g (86%); yellow oil. ^1^H NMR (CDCl_3_, 400 MHz) δ (ppm) = 7.28 (d, *J* = 7.9 Hz, 1H); 7.23 (s, 1H); 7.16 (d, *J* = 7.6 Hz, 1H); 7.09 (dt, *J* = 8.1 and 1.2 Hz, 1H); 4.86 (q, *J* = 6.4 Hz, 1H); 4.16–4.10 (m, 4H); 1.67 (d, *J* = 6.6 Hz, 4H); 1.43–1.35 (m, 4H); 1.46 (d, *J* = 6.5 Hz, 3H); 0.94–0.90 (m, 6H). ^13^C­{^1^H} NMR (CDCl_3_, 100 MHz) δ (ppm) = 151.0 (d, *J*_C–P_ = 6.9 Hz); 148.4; 129.8; 122.1; 118. Nine (d, *J*_C–P_ = 4.7 Hz); 117.3 (d, *J*_C–P_ = 5.0 Hz); 69.9; 68.5 (d, *J*_C–P_ = 6.3 Hz); 32.4 (d, *J*_C–P_ = 6.7 Hz); 25.3; 18.8; 13.7. ^31^P­{^1^H} NMR (CDCl_3,_ 162 MHz) δ (ppm) = −11.8. MS (rel. int., %) *m*/*z*: 330 (3.18), 203 (35.07), 77 (26.88), 43 (35.22), 41 (100.0). HRMS (APCI-QTOF) *m*/*z*: [M + H]^+^ Calcd for C_16_H_27_O_5_P: 331.1668; Found: 331.1669.

#### 4.6.16 Dibutyl (4-(3-hydroxybutyl)­phenyl)­phosphate (**4h**)

Purified by column chromatography (hexane/ethyl acetate = 50:50); yield: 0.176 g (98%); yellowish oil. ^1^H NMR (CDCl_3_, 400 MHz) δ (ppm) = 7.13 (q, *J* = 8.5 Hz, 4H); 4.13 (q, *J* = 5.1 Hz, 4H); 3.80 (sext, *J* = 6.0 Hz, 1H); 2.77–2.60 (m, 2H); 1.77–1.63 (m, 6H); 1.40 (quint, *J* = 7.5 Hz, 4H); 1.22 (d, *J* = 6.1 Hz, 3H); 0.92 (t, *J* = 7.4 Hz, 6H). ^13^C­{^1^H} NMR (CDCl_3_, 100 MHz) δ (ppm) = 149.1 (d, *J*_C–P_ = 7.0 Hz); 139.0; 129.7; 120.0 (d, *J*_C–P_ = 4.8 Hz); 68.4 (d, *J*_C–P_ = 6.3 Hz); 67.5; 32.4 (d, *J*_C–P_ = 6.9 Hz); 31.5; 23.8 18.8; 13.7. ^31^P­{^1^H} NMR (CDCl_3,_ 162 MHz) δ (ppm) = −11.6. MS (rel. int., %) *m*/*z*: 358 (11.80), 213 (40.86), 201 (100.0), 77 (24.82), 43 (30.56). HRMS (APCI-QTOF) *m*/*z*: [M + H]^+^ Calcd for C_18_H_31_O_5_P: 359.1982; Found: 359.1982.

### 4.7 Data of Chiral Compounds (*S*)-**4a–h**

#### 4.7.1 (*S*)-Diethyl (4-(1-hydroxyethyl)­phenyl)­phosphate (**4a**)

Yield: 0.068 g (98%); yellowish oil. [α]_D_ = −34.04.

#### 4.7.2 (*S*)-Diethyl (4-(1-hydroxypropyl)­phenyl)­phosphate (**4b**)

Yield: 0.050 g (70%); yellow oil. [α]_D_ = −17.83.

#### 4.7.3 (*S*)-Diethyl (3-(1-hydroxypropyl)­phenyl)­phosphate (**4c**)

Yield: 0.053 g (77%); yellow oil. [α]_D_ = −22.86.

#### 4.7.4 (*S*)-diethyl (4-(3-hydroxybutyl)­phenyl)­phosphate (**4d**)

Yield: 0.068 g (90%); yellowish oil. [α]_D_ = −7.35.

#### 4.7.5 (*S*)-Dibutyl (4-(1-hydroxyethyl)­phenyl)­phosphate (**4e**)

Yield: 0.061 g (74%); yellow oil. [α]_D_ = −19.74.

#### 4.7.6 (*S*)-Dibutyl (4-propionylphenyl)­phosphate (**4f**)

Yield: 0.026 g (30%); yellowish oil. [α]_D_ = −38.80.

#### 4.7.7 (*S*)-Dibutyl (3-(1-hydroxyethyl)­phenyl)­phosphate (**4g**)

Yield: 0.041 g (50%); yellow oil. [α]_D_ = −11.75.

#### 4.7.8 (*S*)-Dibutyl (4-(3-hydroxybutyl)­phenyl)­phosphate (**4h**)

Yield: 0.071 g (79%); yellowish oil. [α]_D_ = −9.08.

## Supplementary Material



## Notes

Spectral data (^1^H, ^13^C, ^31^P NMR) for compounds **3a**–**h** (Figures S3–S26) and **4a**–**h** (Figures S27–S50); GC/MS and HRMS data for compounds **3** and **4**; Chiral NMR (^1^H and ^31^P) discrimination data for the racemic (Figures S51–S57 and S59–S64) and enantiomerically enriched (Figures S67–S78) compounds **4a**, **4b**, **4c**, **4e**, **4f**, and **4g**; chiral GC-FID analyses for compounds *rac*-**4a**, *rac*-**4b**, *rac*-**4d**, and *rac*-**4h** (Figures S85–S88); and values of optical rotation analysis for compounds (*S*)-**4**. (PDF)
