# Supplementary material for: Sonochemistry and Biocatalysis: Two-Step Green Asymmetric Synthesis of Optically Active Dialkyl(4-(hydroxyalkyl)phenyl)phosphates
Source: ACS Omega. 2025 Jun 30;10(27):29452–62. doi: 10.1021/acsomega.5c02774 (PMC12268435; doi:10.1021/acsomega.5c02774)
Supplement: Supplementary file 1 [file ao5c02774_si_001.pdf]

SUPPORTING INFORMATION

Sonochemistry and Biocatalysis: Two-Step Green Asymmetric Synthesis of Optically Active Dialkyl(4-(hydroxyalkyl)phenyl)phosphates

Lucas Emanuel Beluzzo Iarocz,[a] Marcela Belen Alvarez,[a] Amanda Goldbeck Gerbaudo,[a] Eder João Lenardão,[a] Gelson Perin[a]* and Márcio Santos Silva[a]*

[a] Laboratório de Síntese Orgânica Limpa (LASOL), Centro de Ciências Químicas, Farmacêuticas e de Alimentos (CCQFA), Universidade Federal de Pelotas (UFPel) - P.O. Box 354 - 96010-900, Pelotas, RS, Brazil.

Table of Contents

Contents	Page
Optimization of the Chemical Reduction	S2
Scheme S1	S3
Figure S1	S4
Figure S2	S5
Selected NMR Spectra: Compounds **3a-h**	S6
Selected NMR Spectra: Compounds **4a-h**	S30
Selected NMR Spectra: Chiral Discrimination by ^{1}H and ^{31}P NMR Spectroscopies	S54
Selected Chiral Chromatography Analyses	S88

Optimization of the Chemical Reduction

Table S1. Exploratory study in the optimization of reaction conditions to obtain the racemic hydroxyphosphonate **4a** [a,b]

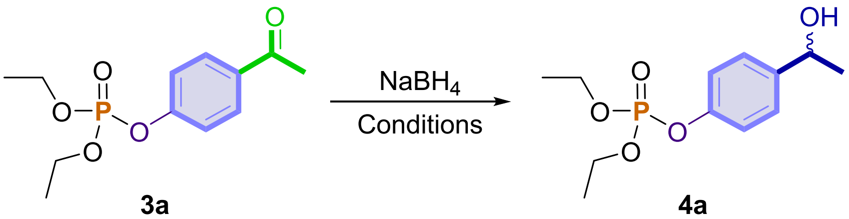

Entry	$NaBH_4$ (equiv.)	Solvent	Time (h)	Yield (%)
1	1.0	CH_3OH	6	88
2	1.0	EtOH	0.5	89
3[c]	1.0	PEG-400	1	96
4[d]	1.0	EtOH:H_2O	0.25	90
**5**	**1.2**	**EtOH:H_2O**	**0.25**	**99**

[a] Reaction performed using **3a** (0.25 mmol), sodium borohydride ($NaBH_4$) and 2.0 mL of solvent. [b] Isolated yields obtained after column chromatography. [c] Reaction at 50 ºC. [d] Mixture of EtOH and H_2O (9:1).

Scheme S1. CSAs evaluated in the $^{31}P\{^1H\}$ NMR experiments for the chiral discrimination of *O*,*O*-dialkyl-*O*-phenylphosphonate **4a.**

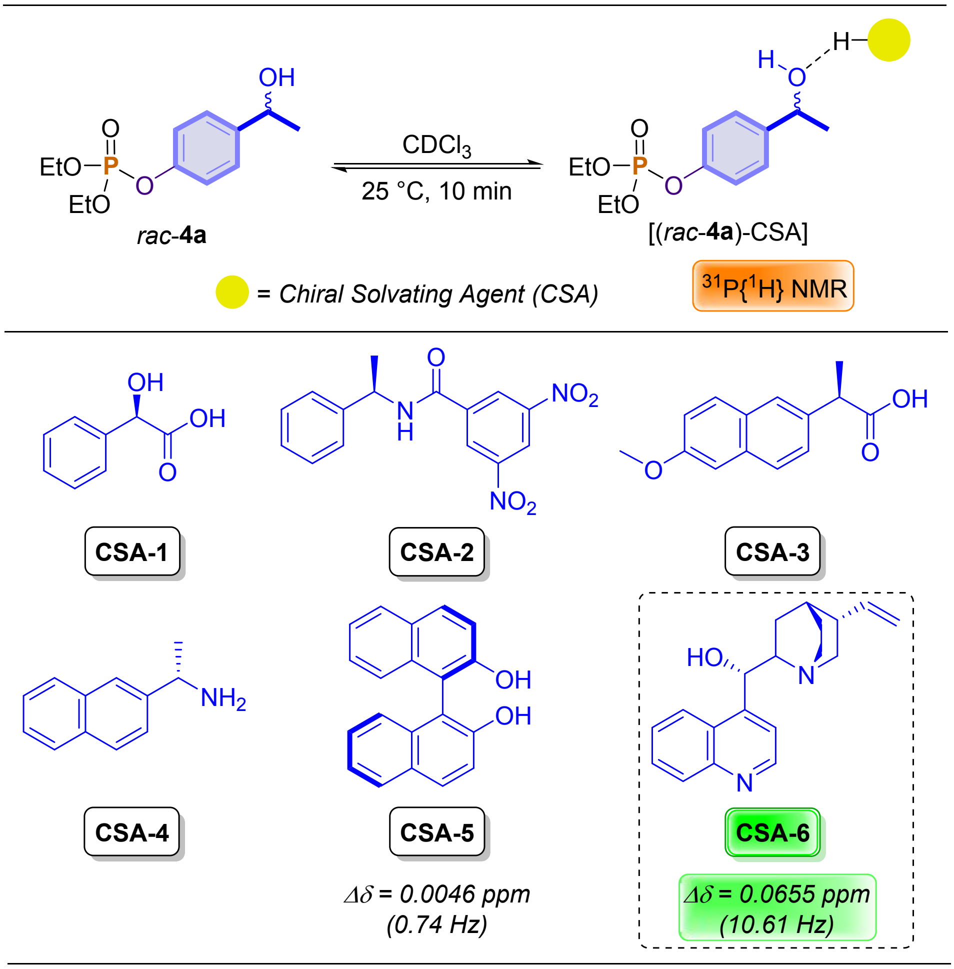

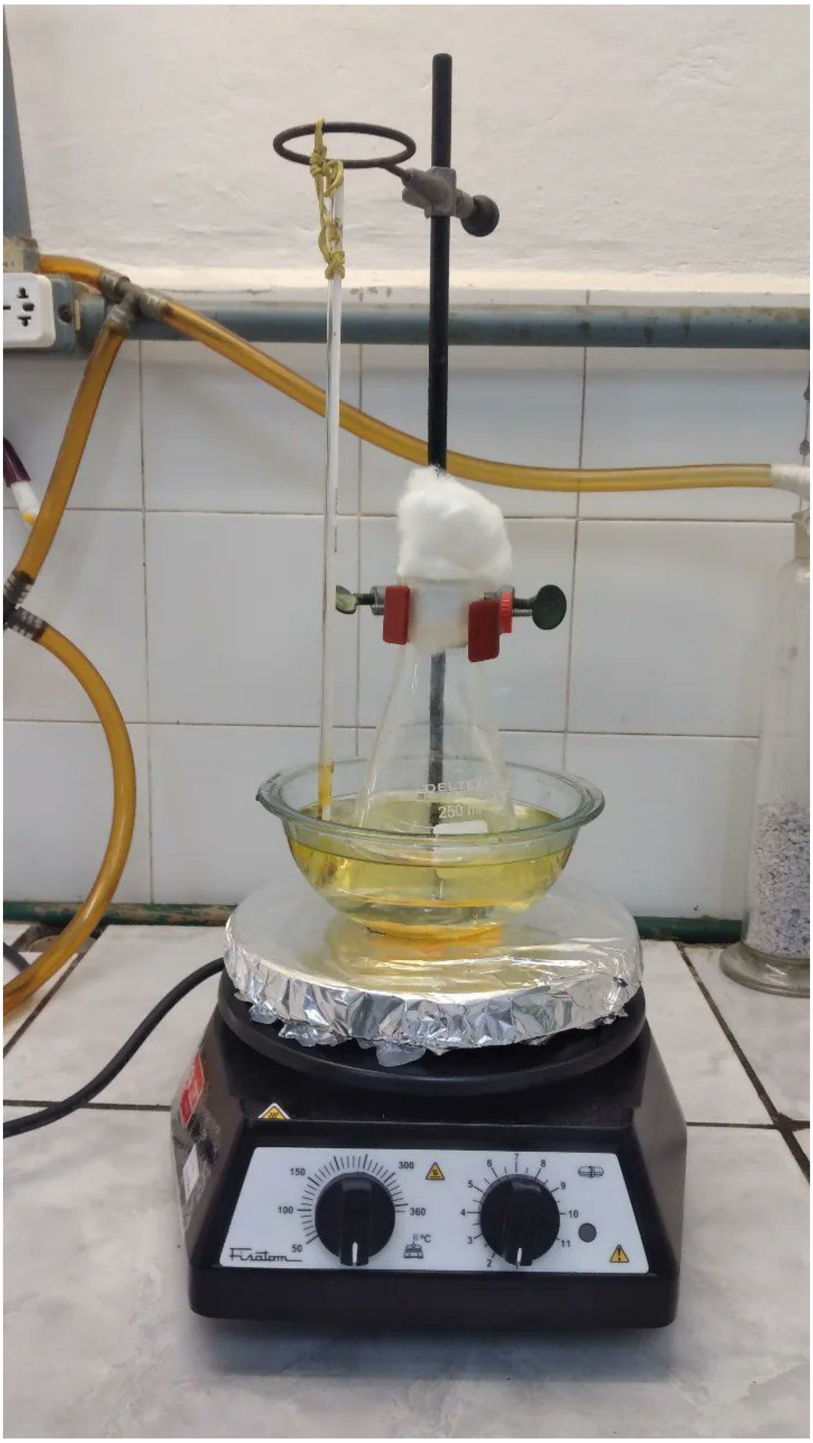

Figure S1. Reaction system used for bioreductions with carrot bits

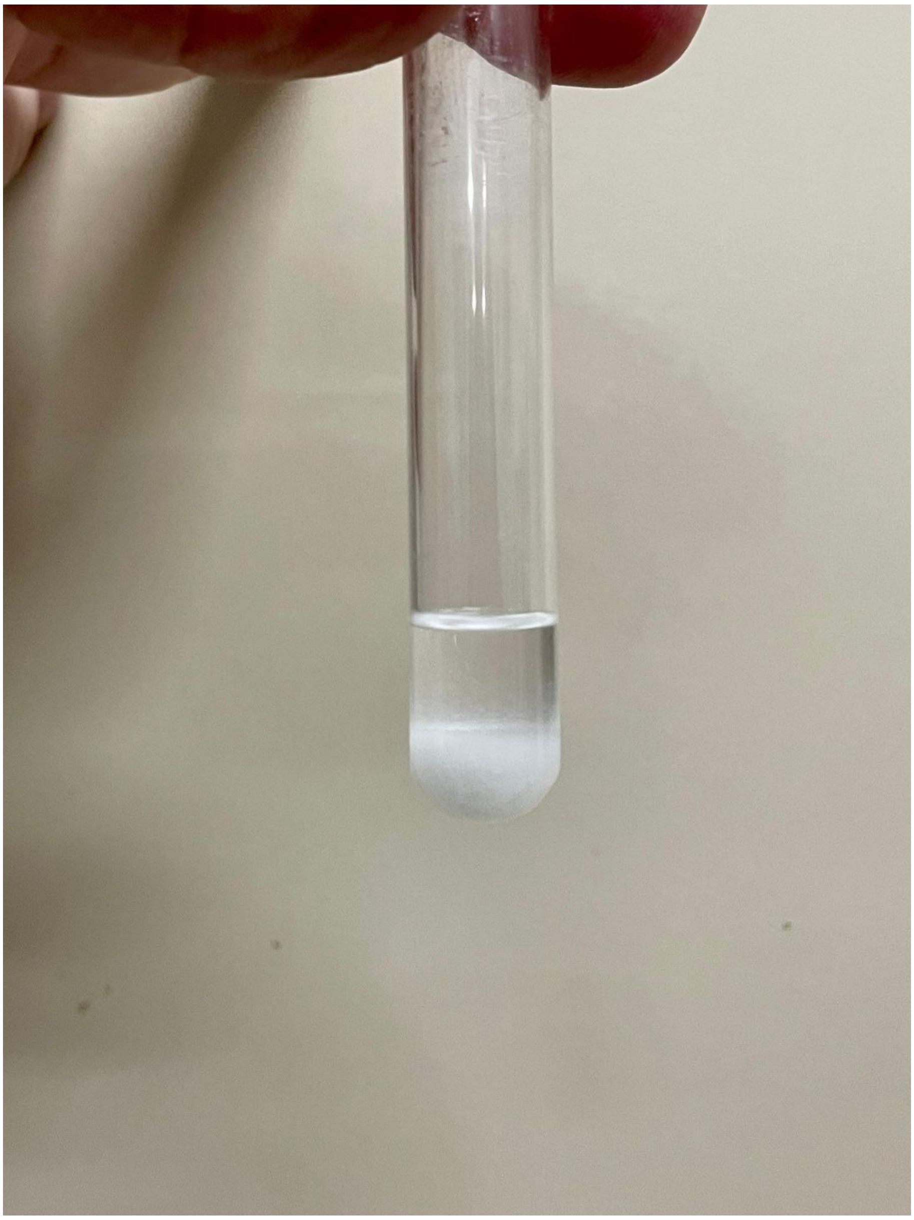

Figure S2. (-)-Cinchonidine solubility test in benzene-d_6

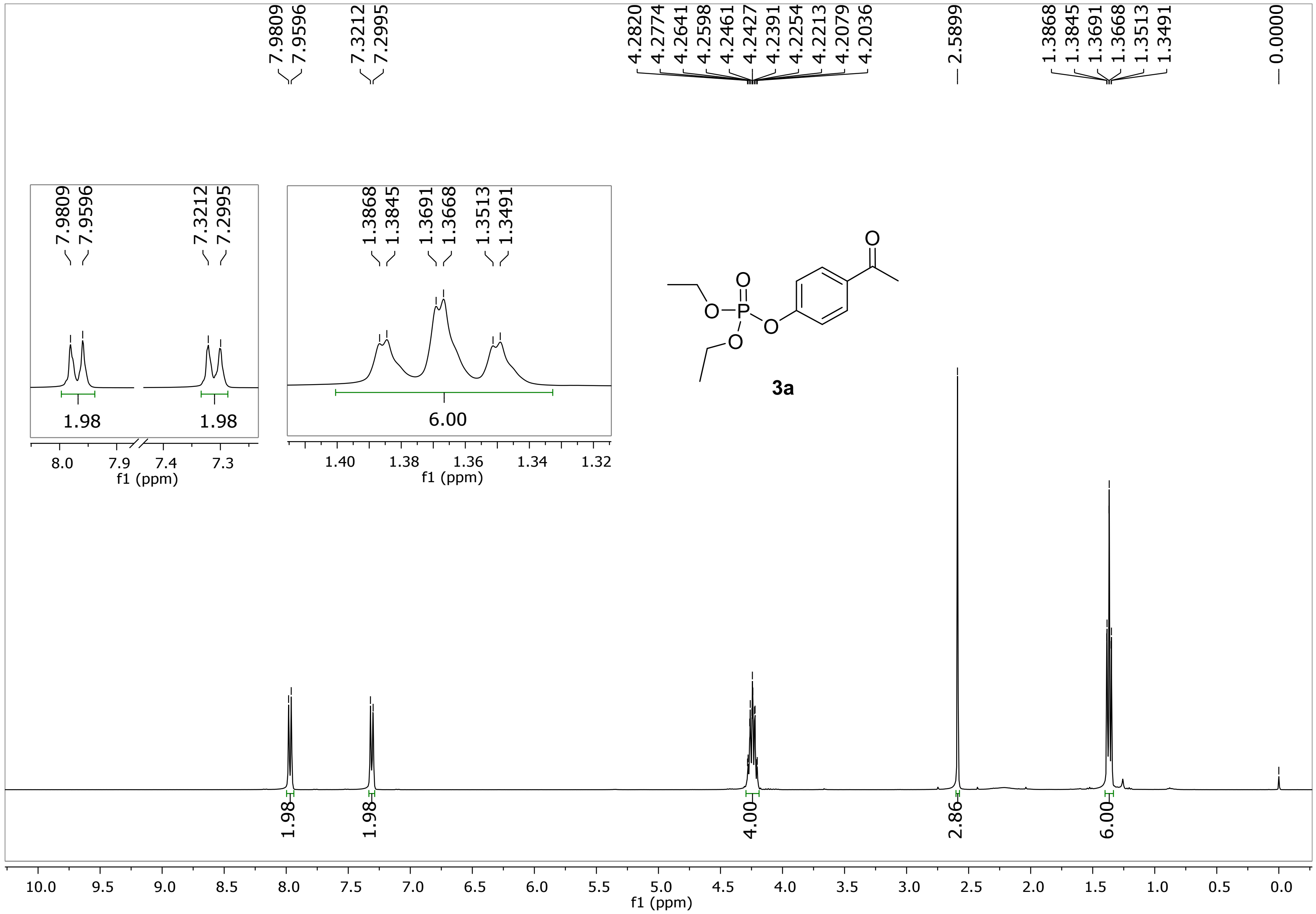

Figure S3. ^{1}H NMR (400 MHz, $CDCl_3$) spectrum of compound **3a**

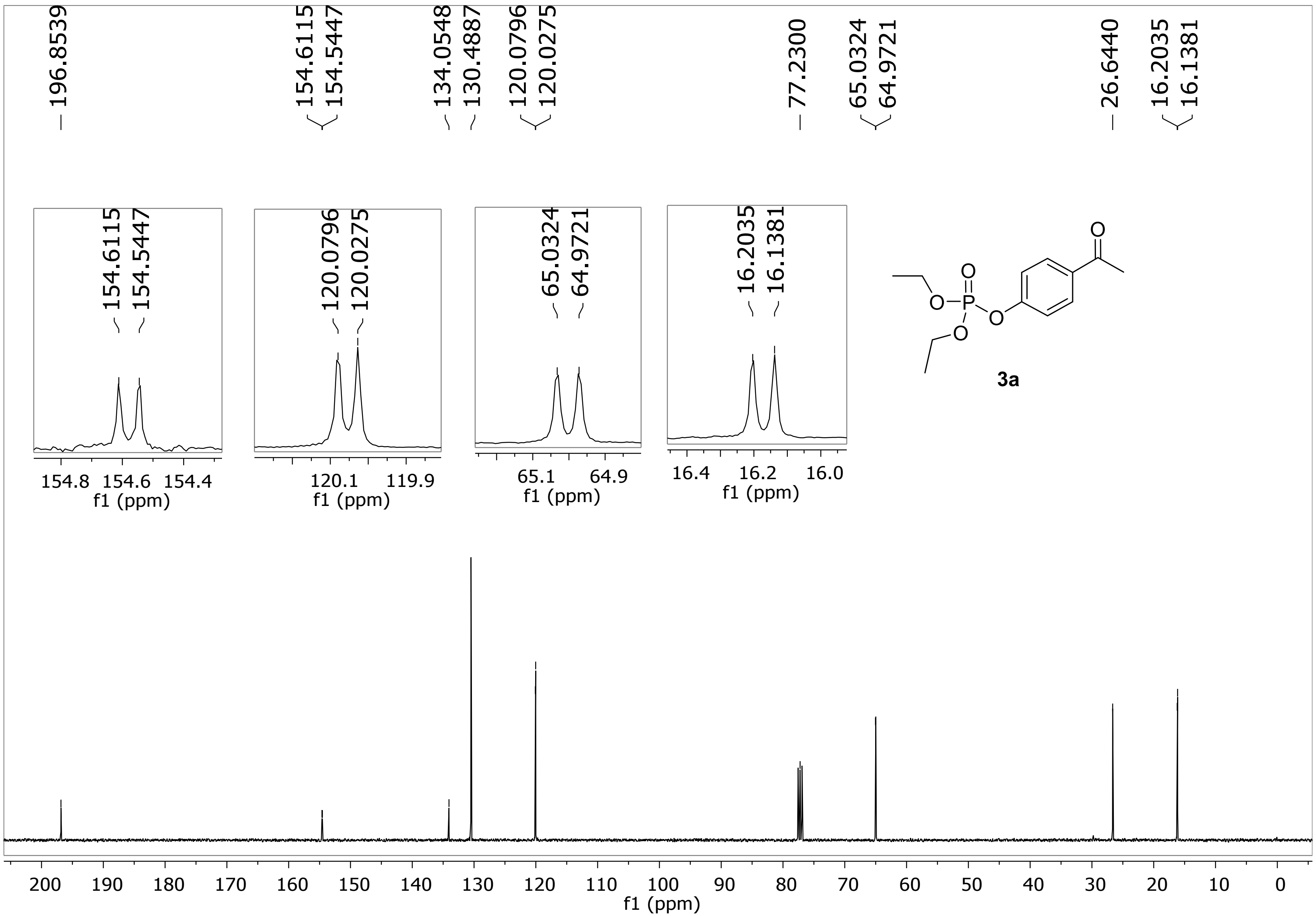

Figure S4. $^{13}C\{^1H\}$ NMR (100 MHz, $CDCl_3$) spectrum of compound **3a**

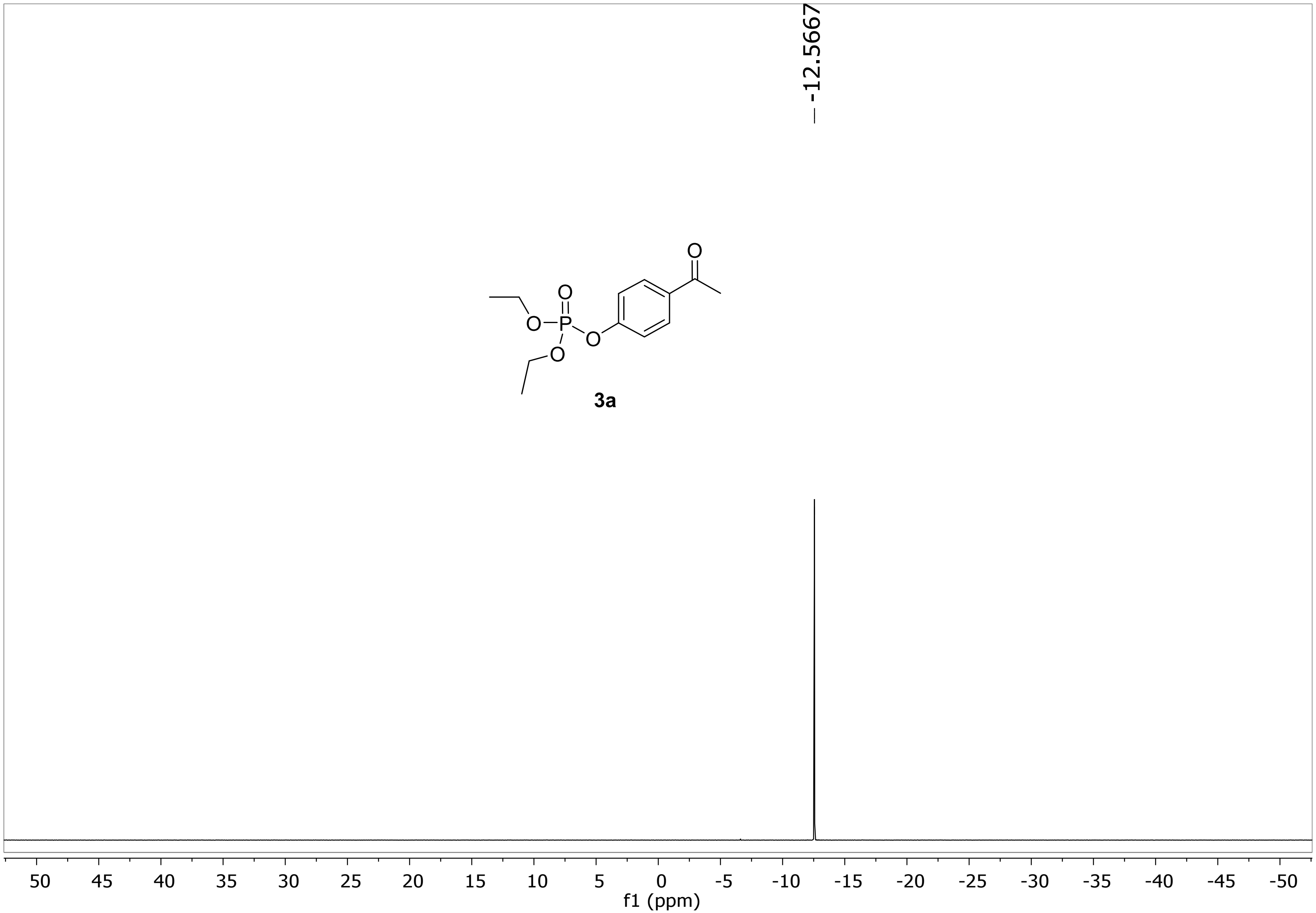

Figure S5. $^{31}P\{^{1}H\}$ NMR (162 MHz, $CDCl_3$) spectrum of compound **3a**

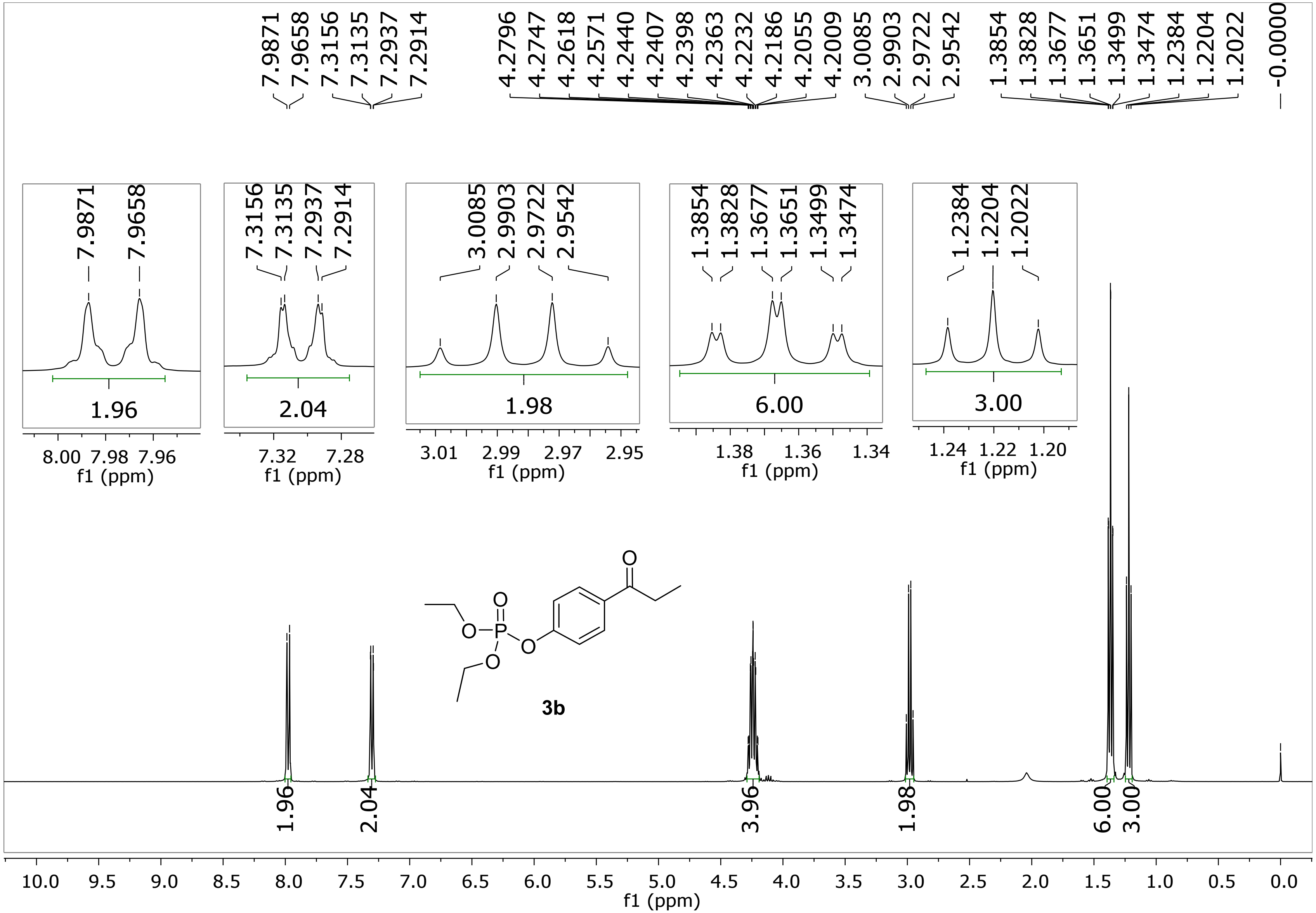

Figure S6. ^{1}H NMR (400 MHz, $CDCl_3$) spectrum of compound **3b**

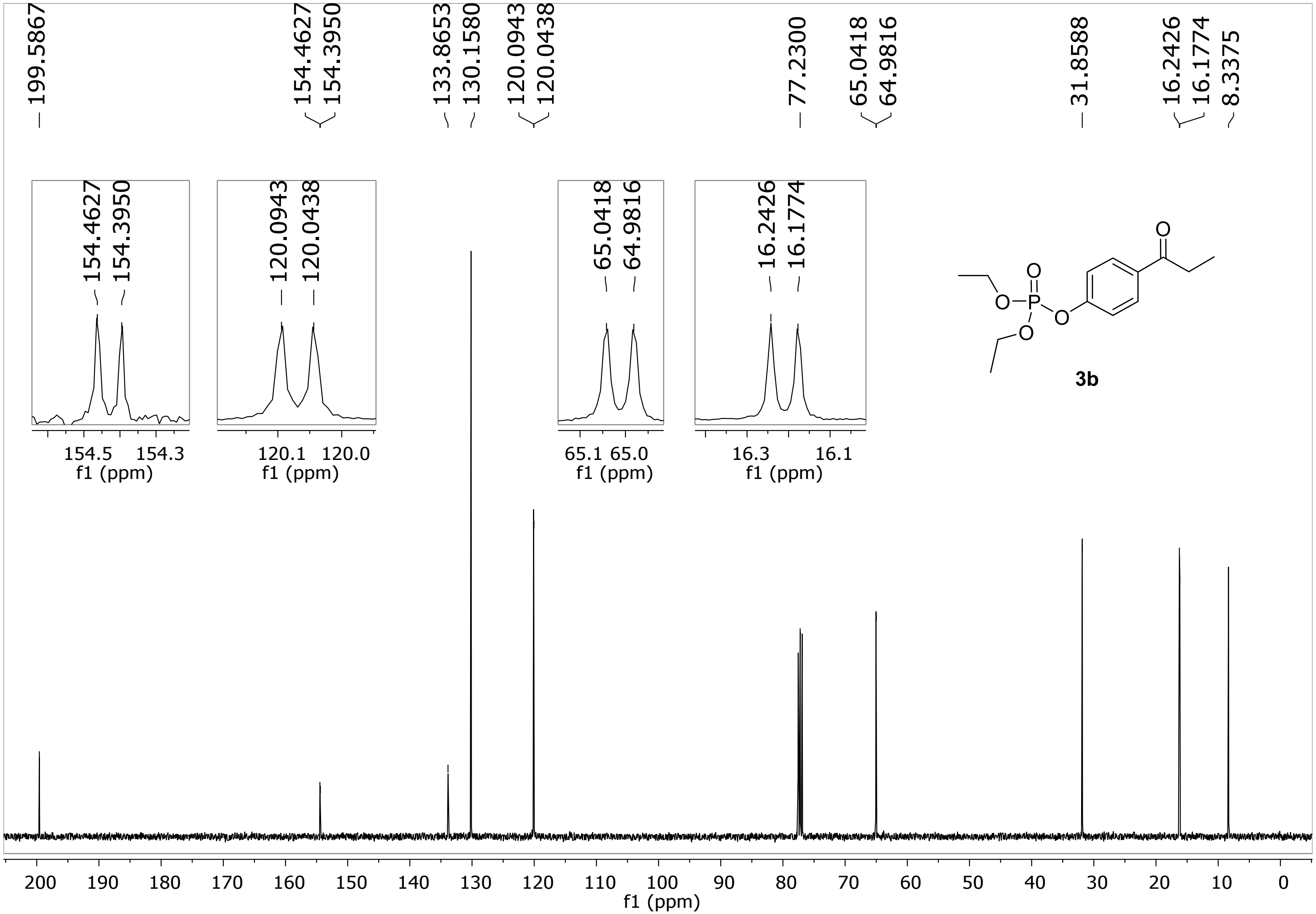

Figure S7. $^{13}C\{^1H\}$ NMR (100 MHz, $CDCl_3$) spectrum of compound **3b**

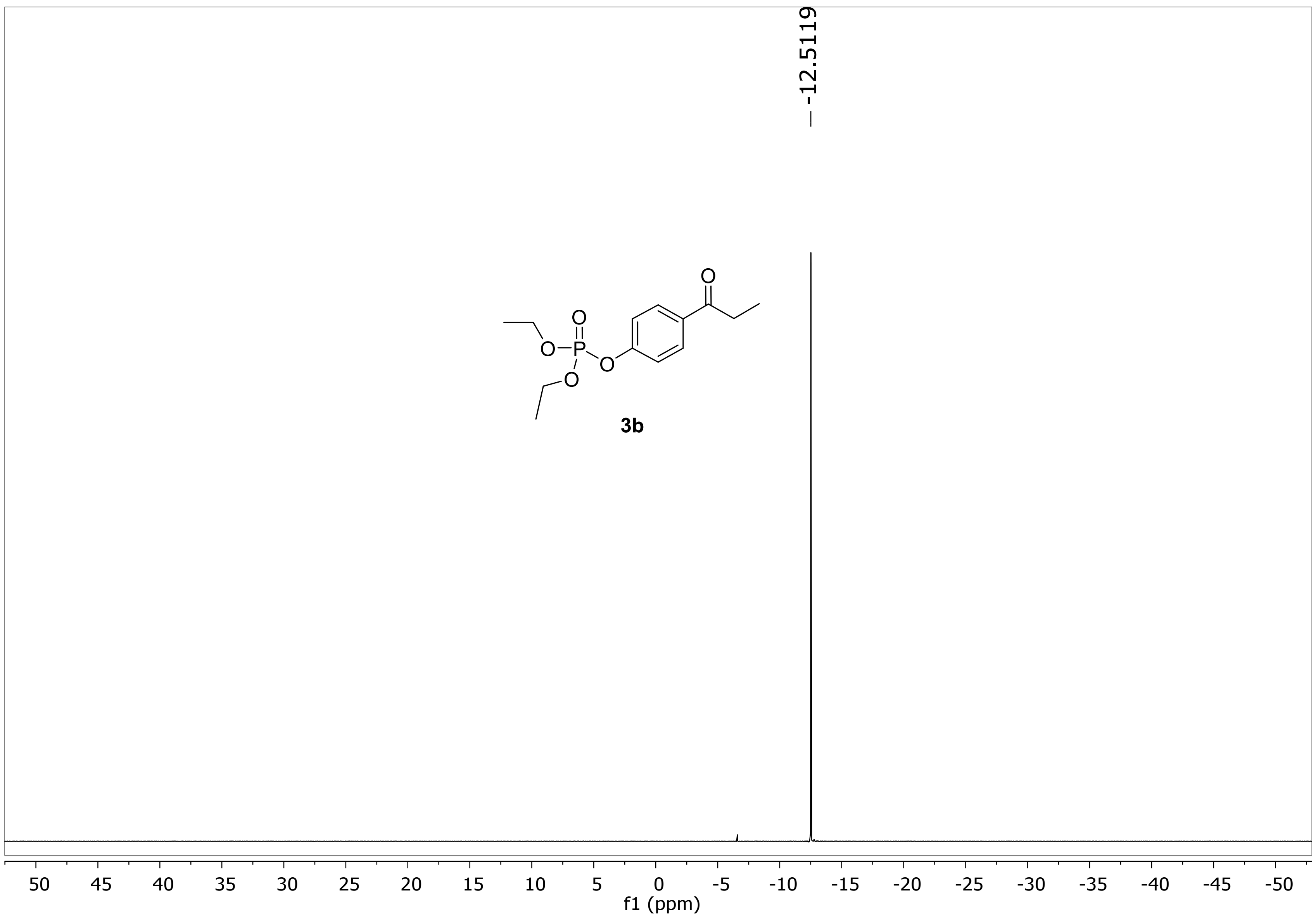

Figure S8. $^{31}P\{^{1}H\}$ NMR (162 MHz, $CDCl_3$) spectrum of compound **3b**

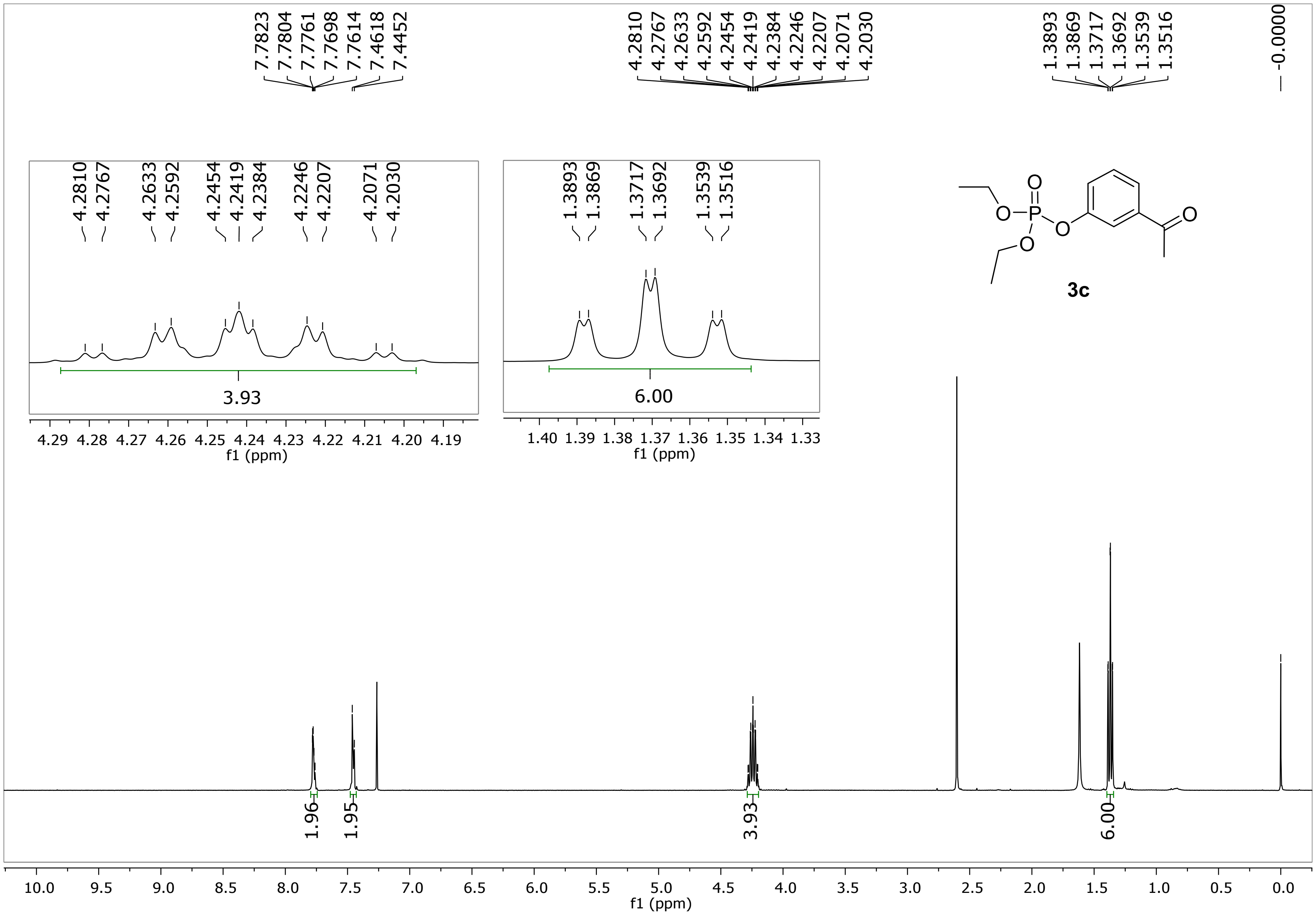

Figure S9. ^{1}H NMR (400 MHz, $CDCl_3$) spectrum of compound **3c**

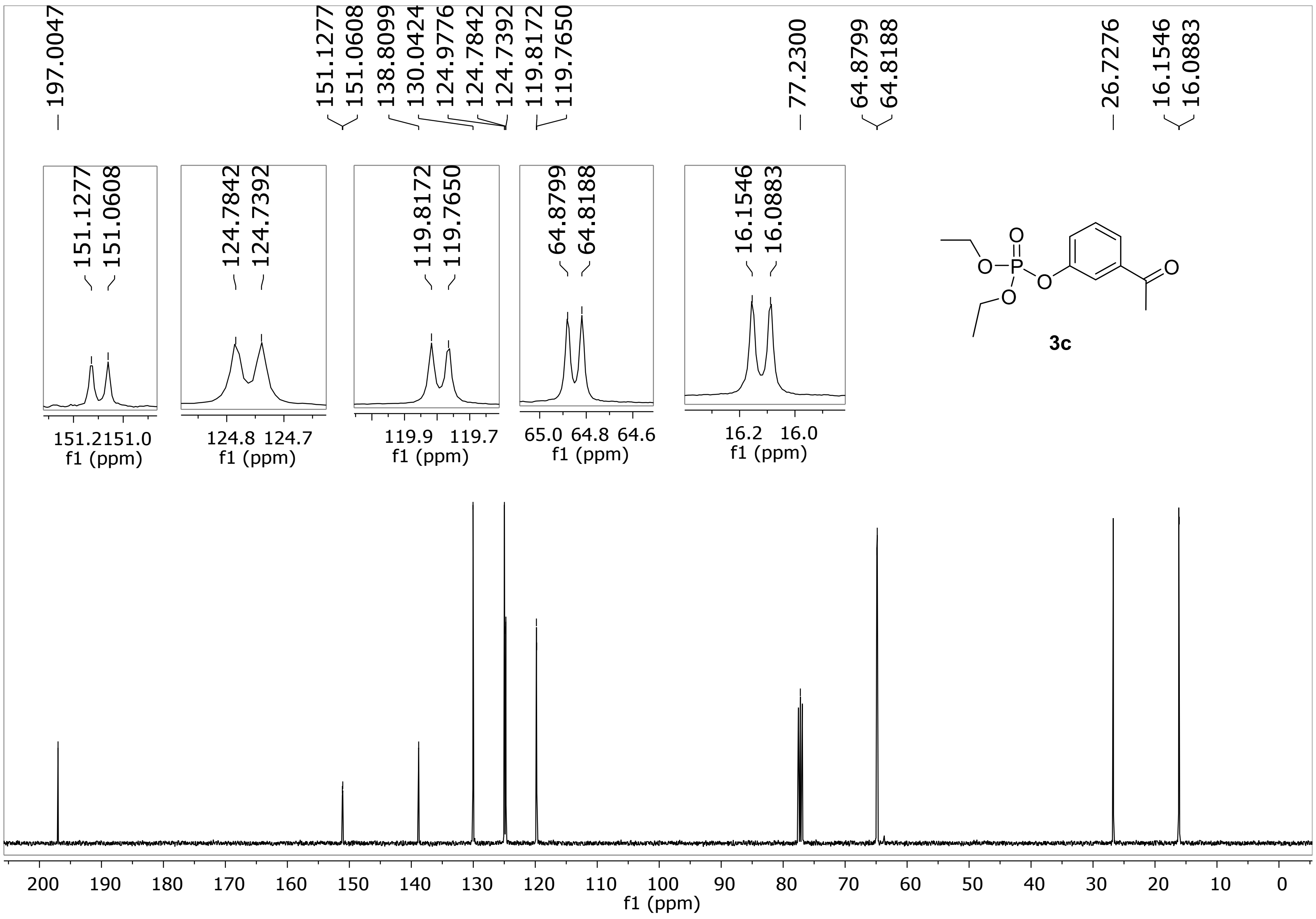

Figure S10. $^{13}C\{^{1}H\}$ NMR (100 MHz, $CDCl_3$) spectrum of compound **3c**

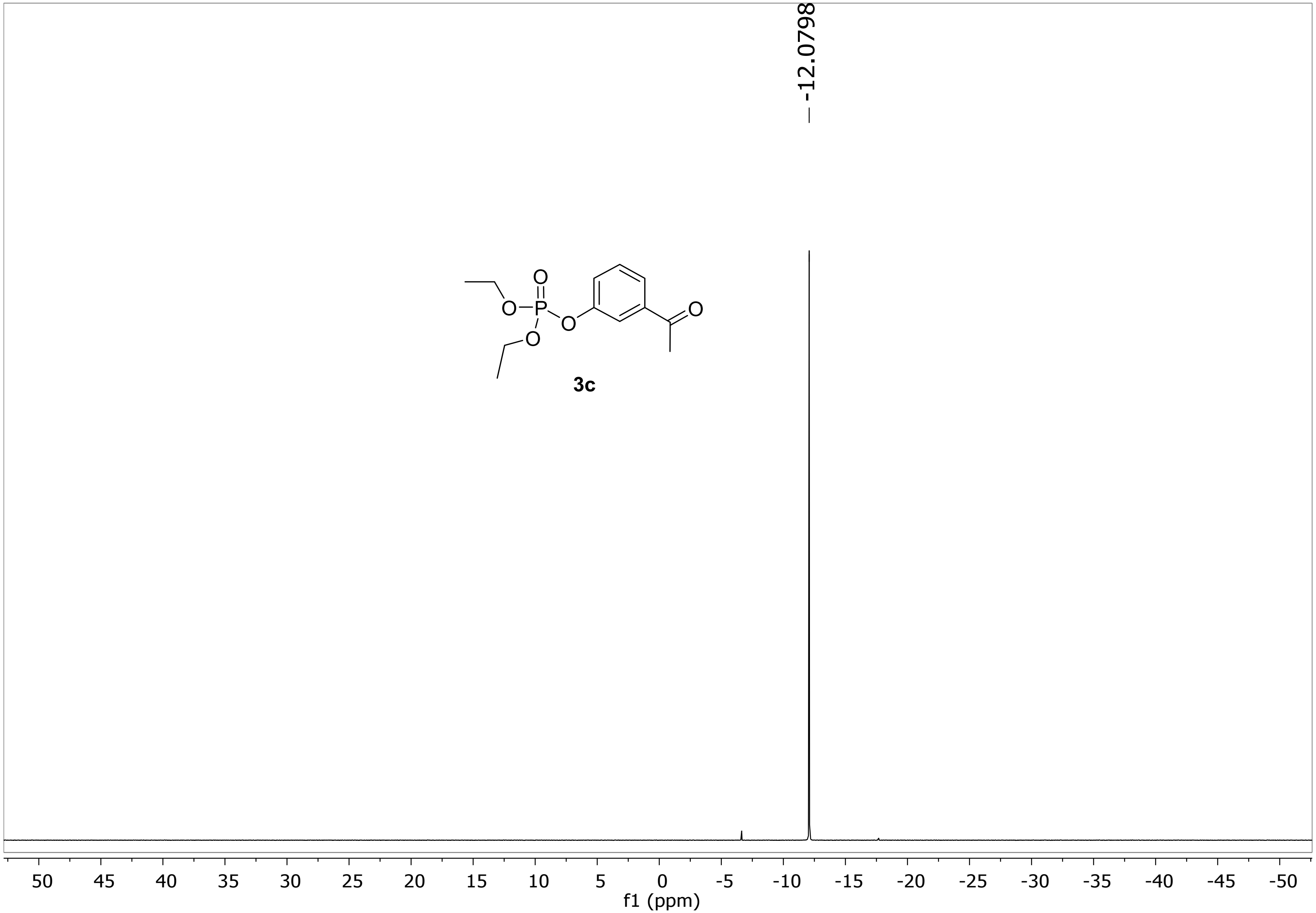

Figure S11. $^{31}P\{^{1}H\}$ NMR (162 MHz, $CDCl_3$) spectrum of compound **3c**

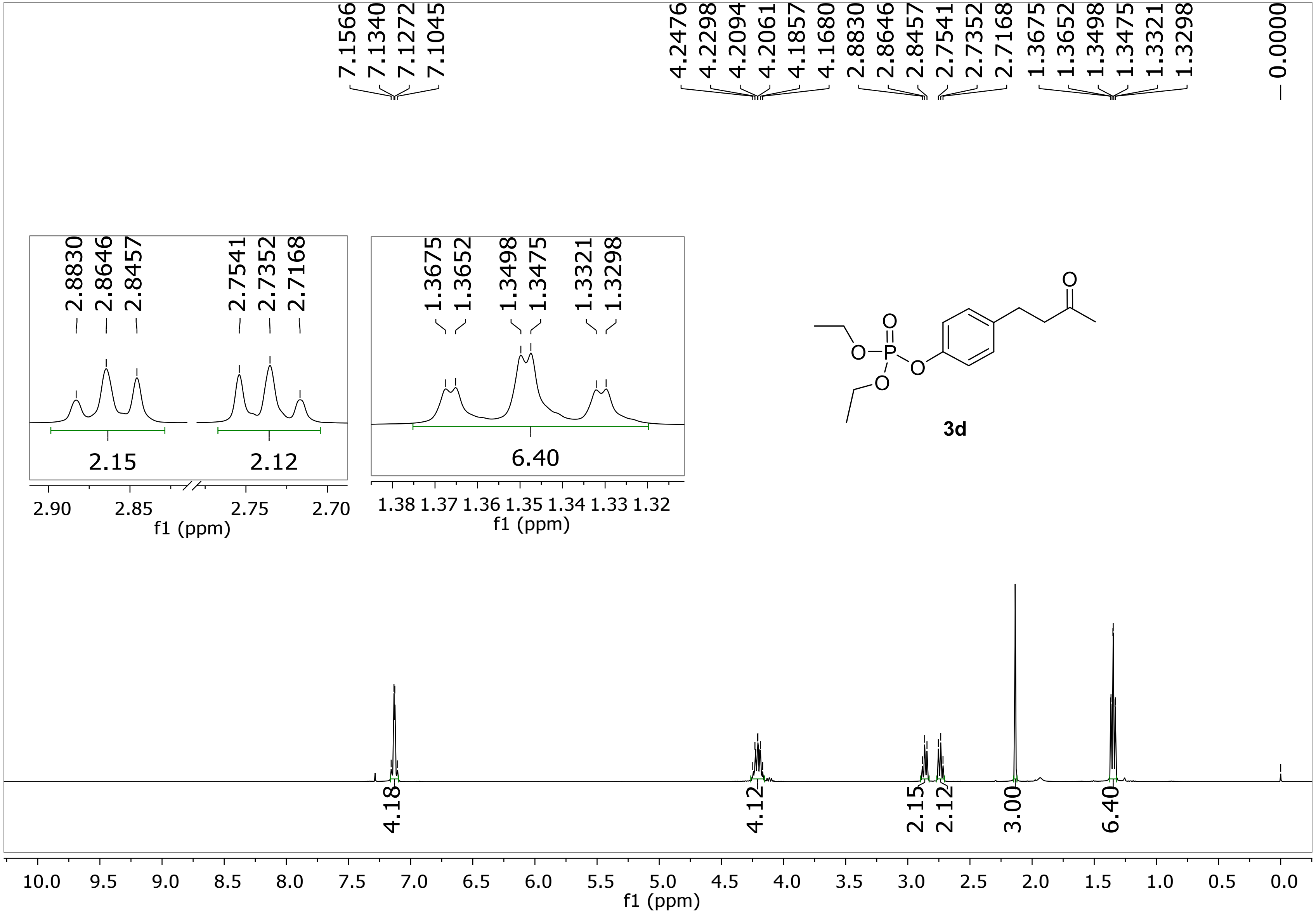

Figure S12. ^{1}H NMR (400 MHz, $CDCl_3$) spectrum of compound **3d**

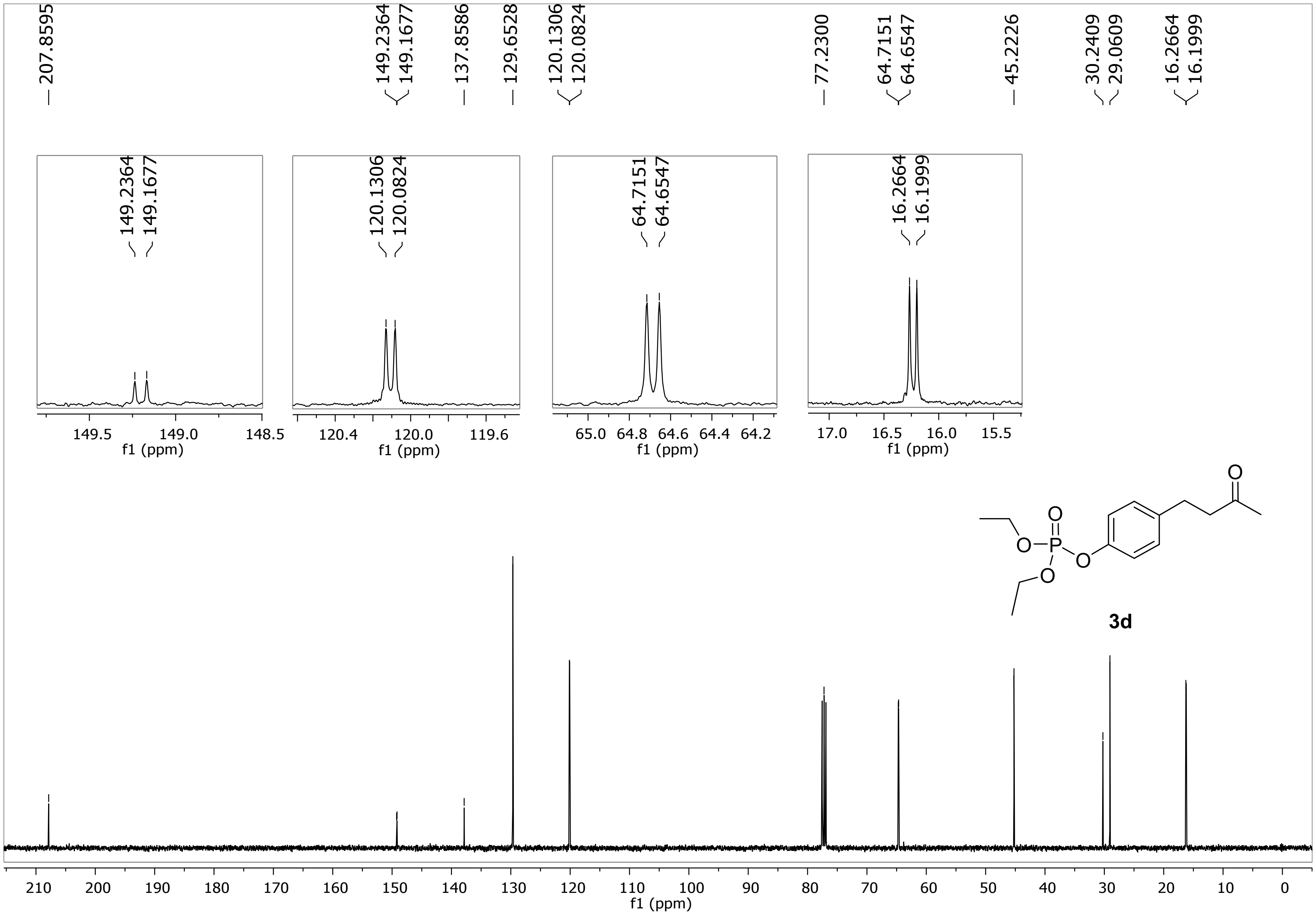

Figure S13. $^{13}C\{^{1}H\}$ NMR (100 MHz, $CDCl_3$) spectrum of compound **3d**

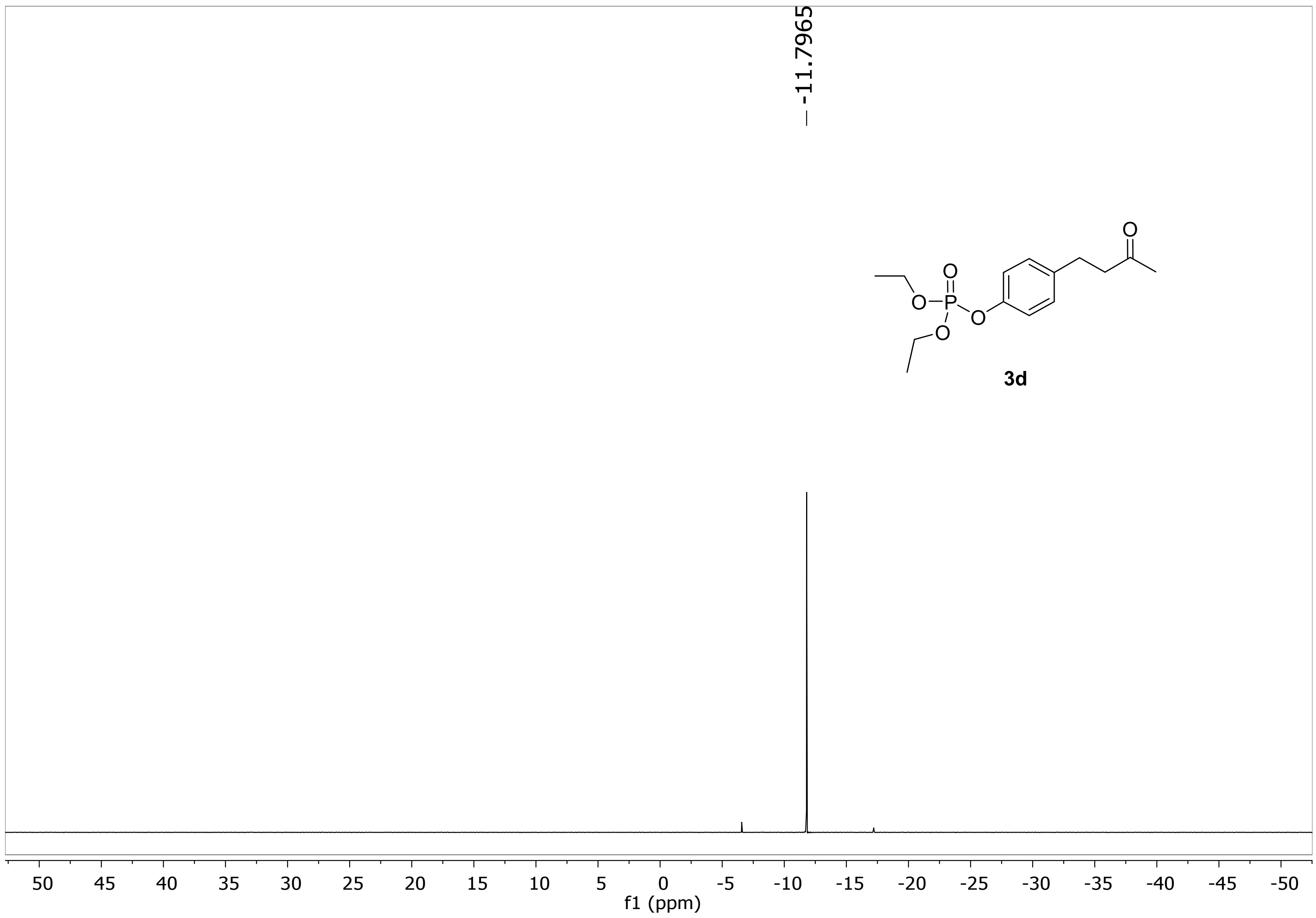

Figure S14. $^{31}P\{^{1}H\}$ NMR (162 MHz, $CDCl_3$) spectrum of compound **3d**

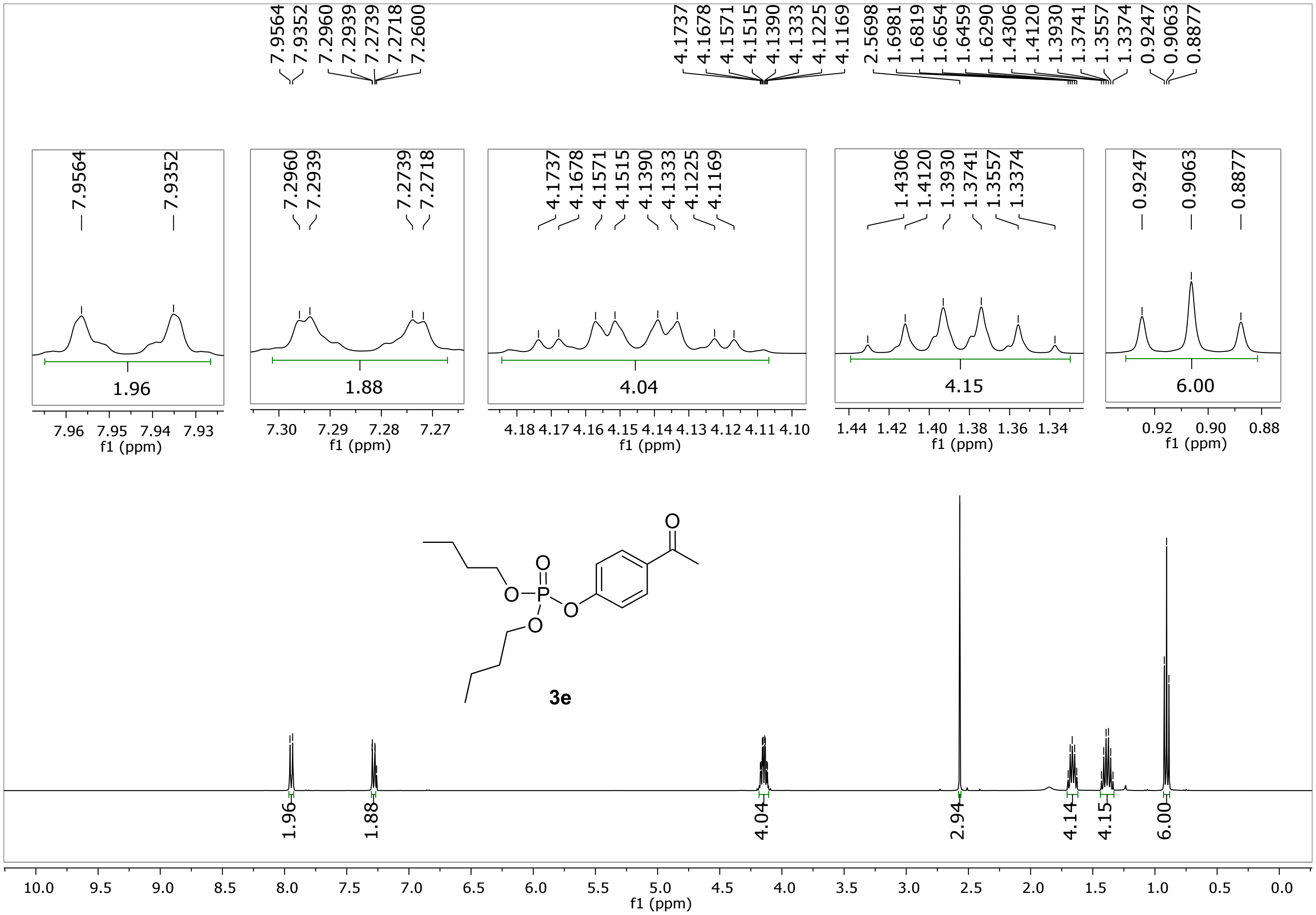

Figure S15. ^{1}H NMR (400 MHz, $CDCl_3$) spectrum of compound **3e**

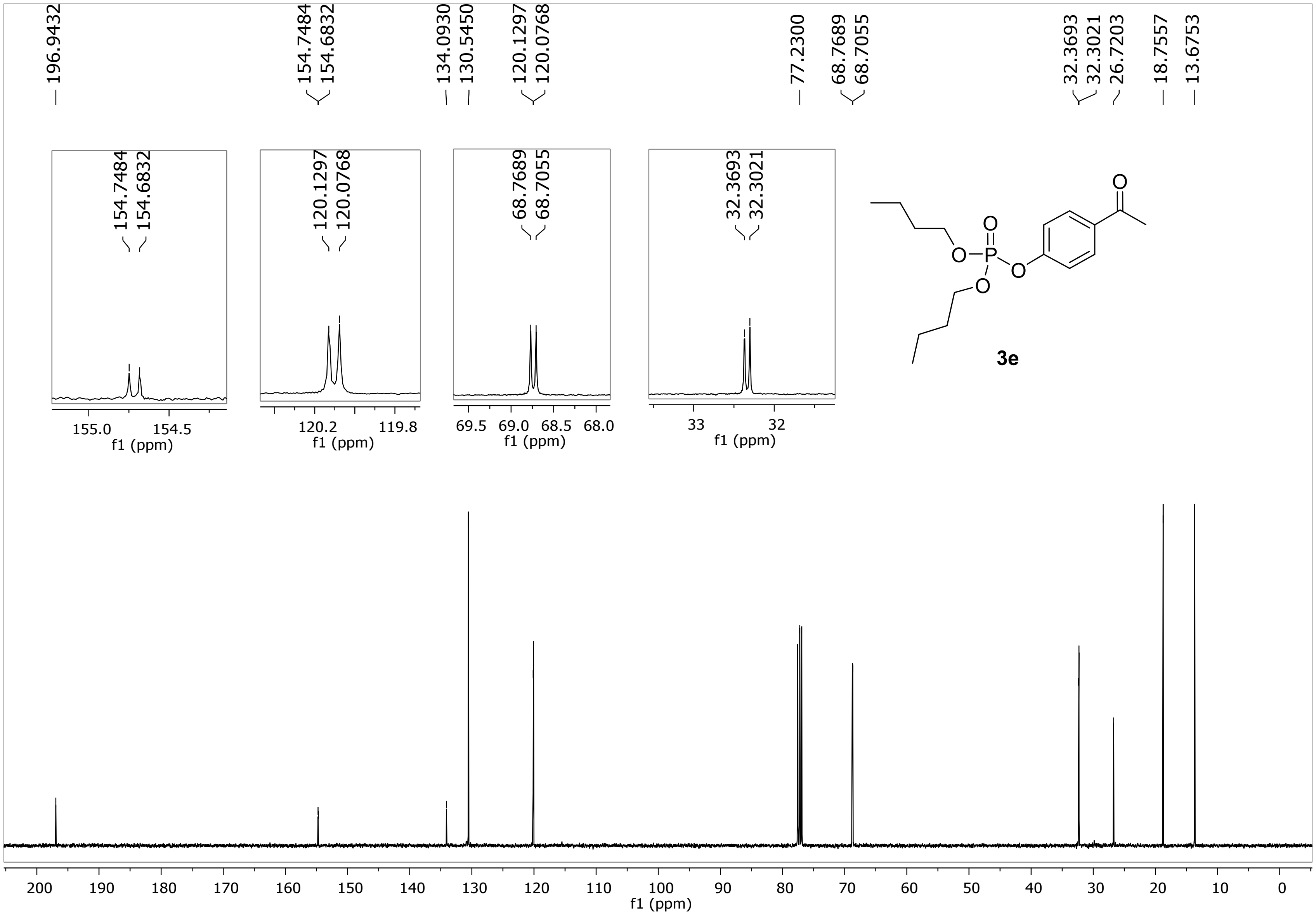

Figure S16. $^{13}C\{^{1}H\}$ NMR (100 MHz, $CDCl_3$) spectrum of compound **3e**

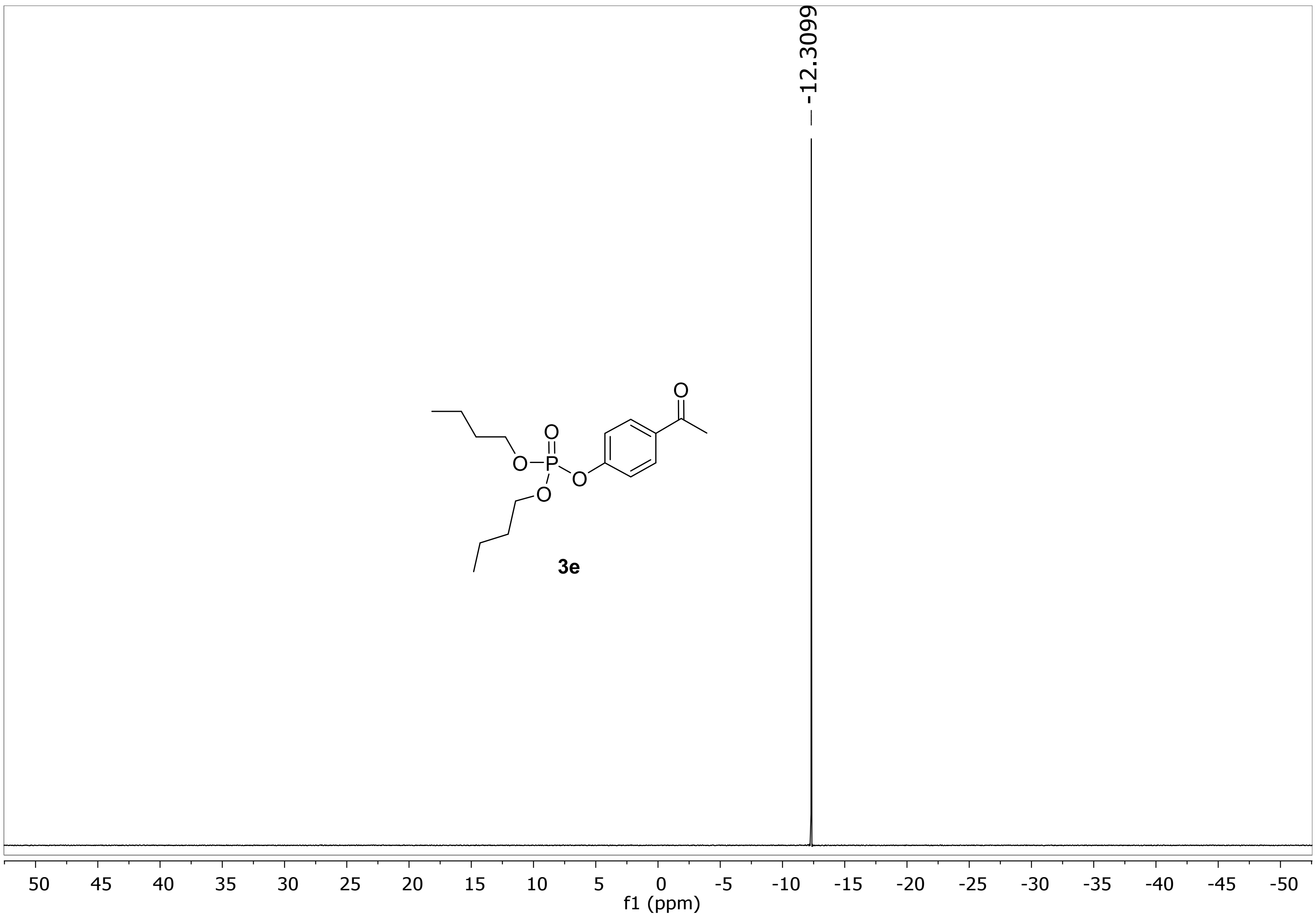

Figure S17. $^{31}P\{^{1}H\}$ NMR (162 MHz, $CDCl_3$) spectrum of compound **3e**

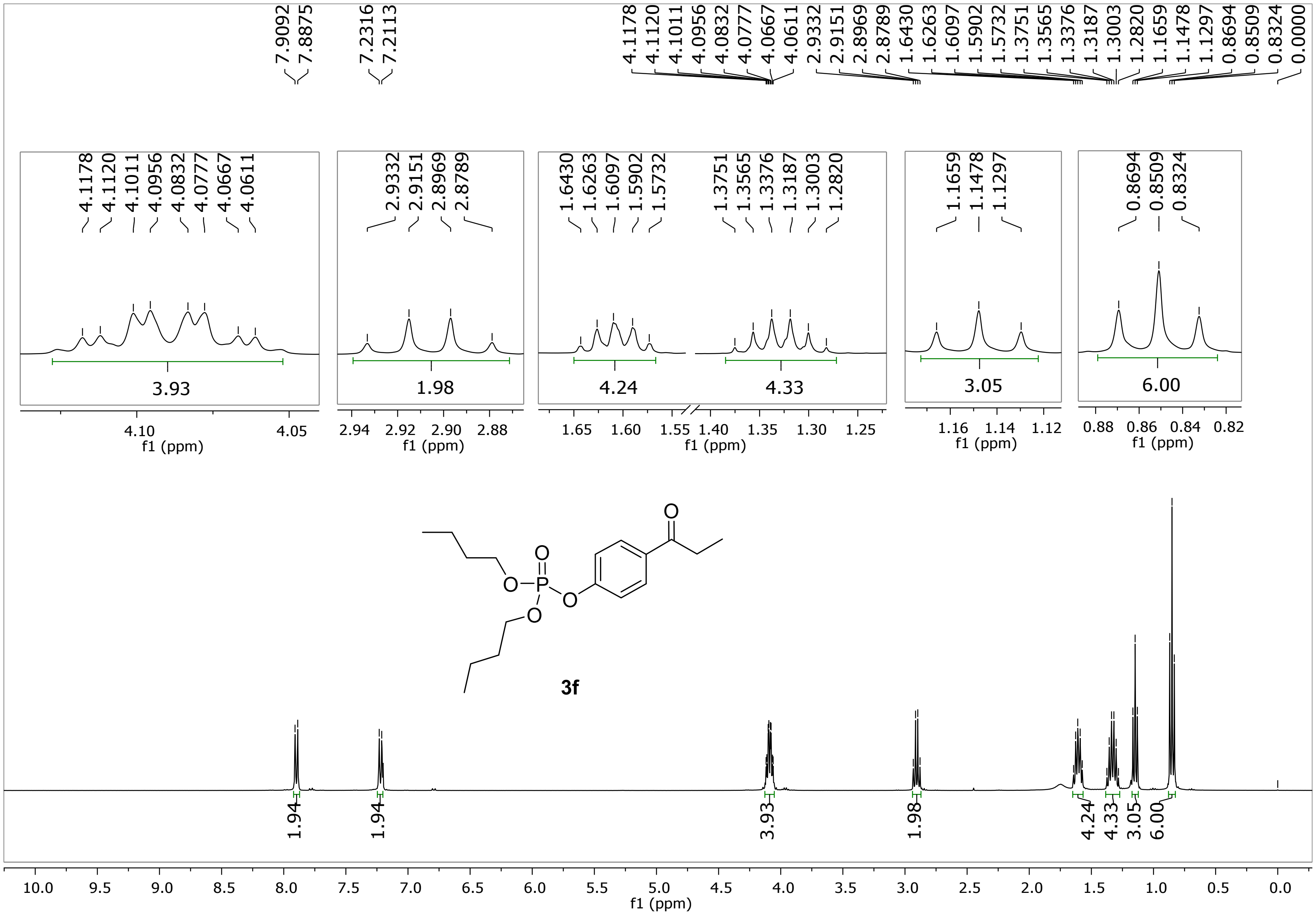

Figure S18. ^{1}H NMR (400 MHz, $CDCl_3$) spectrum of compound **3f**

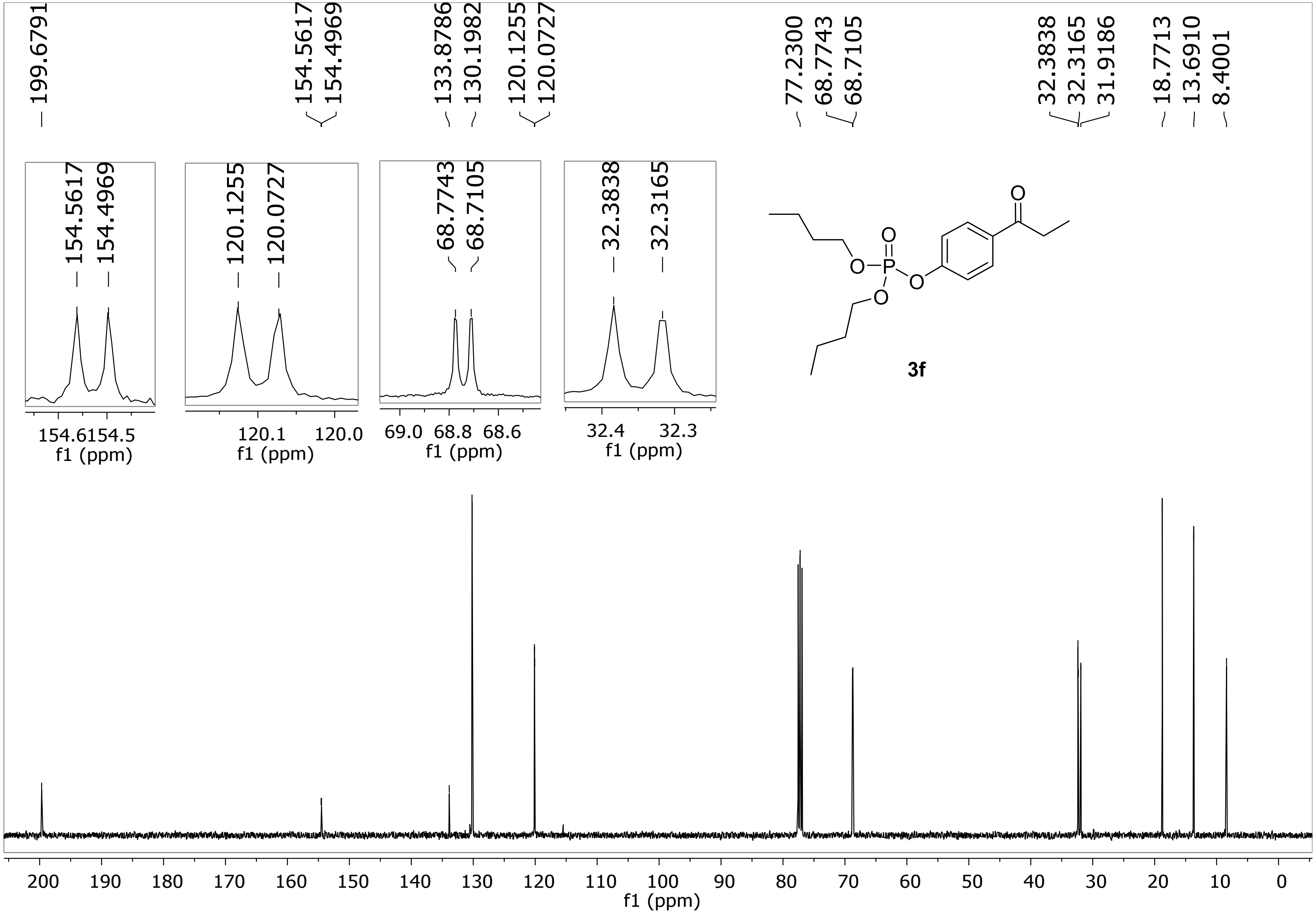

Figure S19. $^{13}C\{^{1}H\}$ NMR (100 MHz, $CDCl_3$) spectrum of compound **3f**

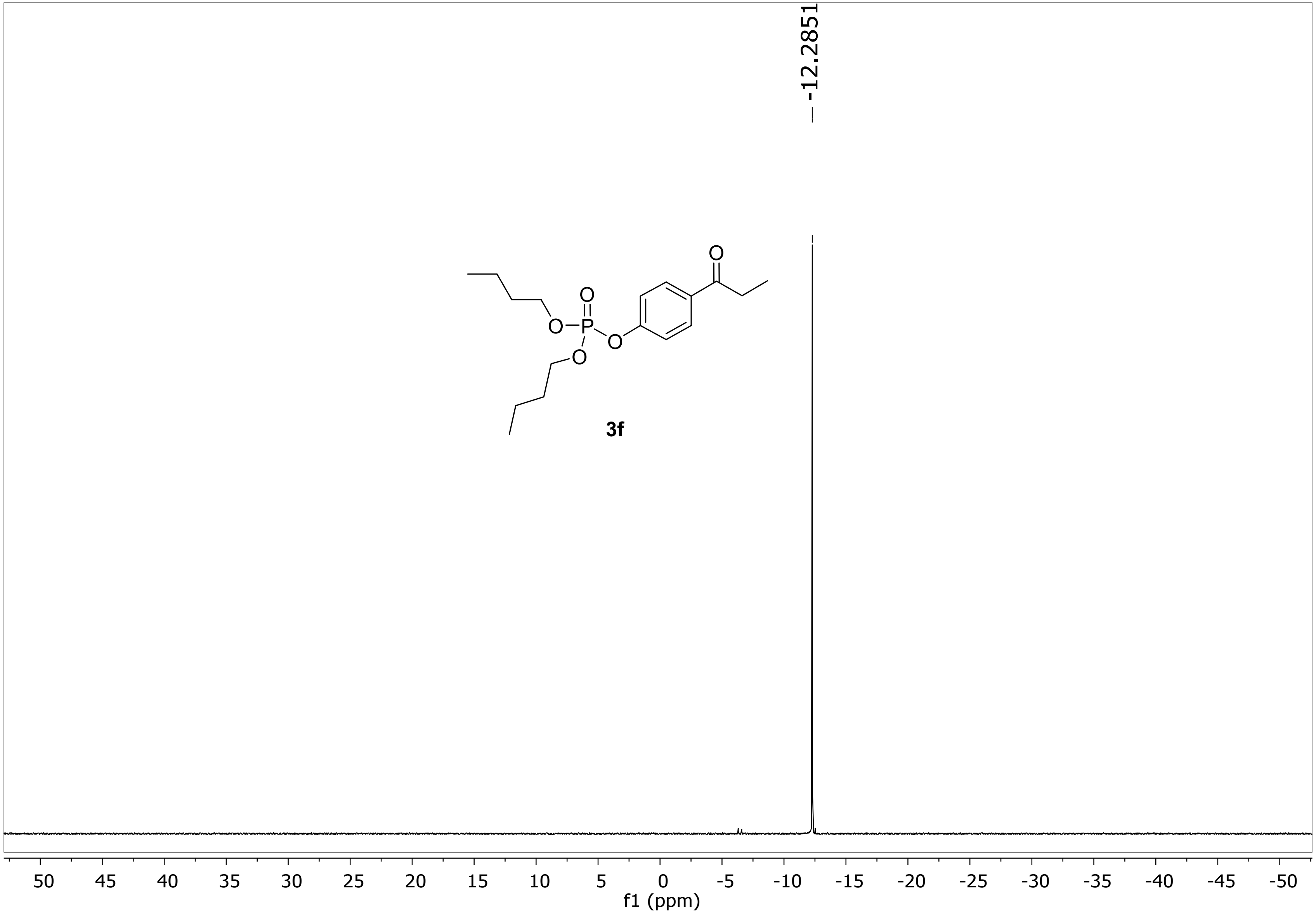

Figure S20. $^{31}P\{^{1}H\}$ NMR (162 MHz, $CDCl_3$) spectrum of compound **3f**

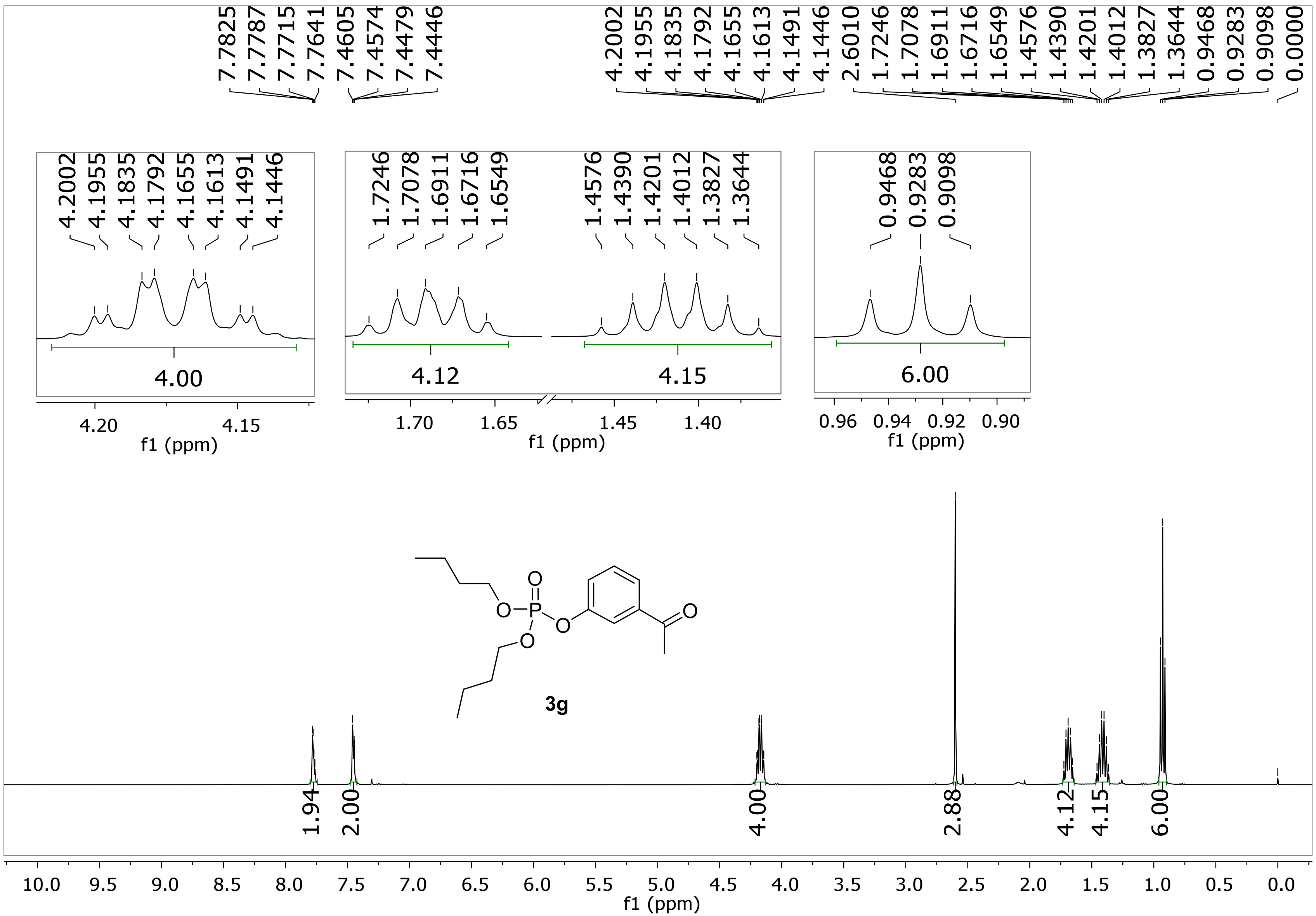

Figure S21. ^{1}H NMR (400 MHz, $CDCl_3$) spectrum of compound **3g**

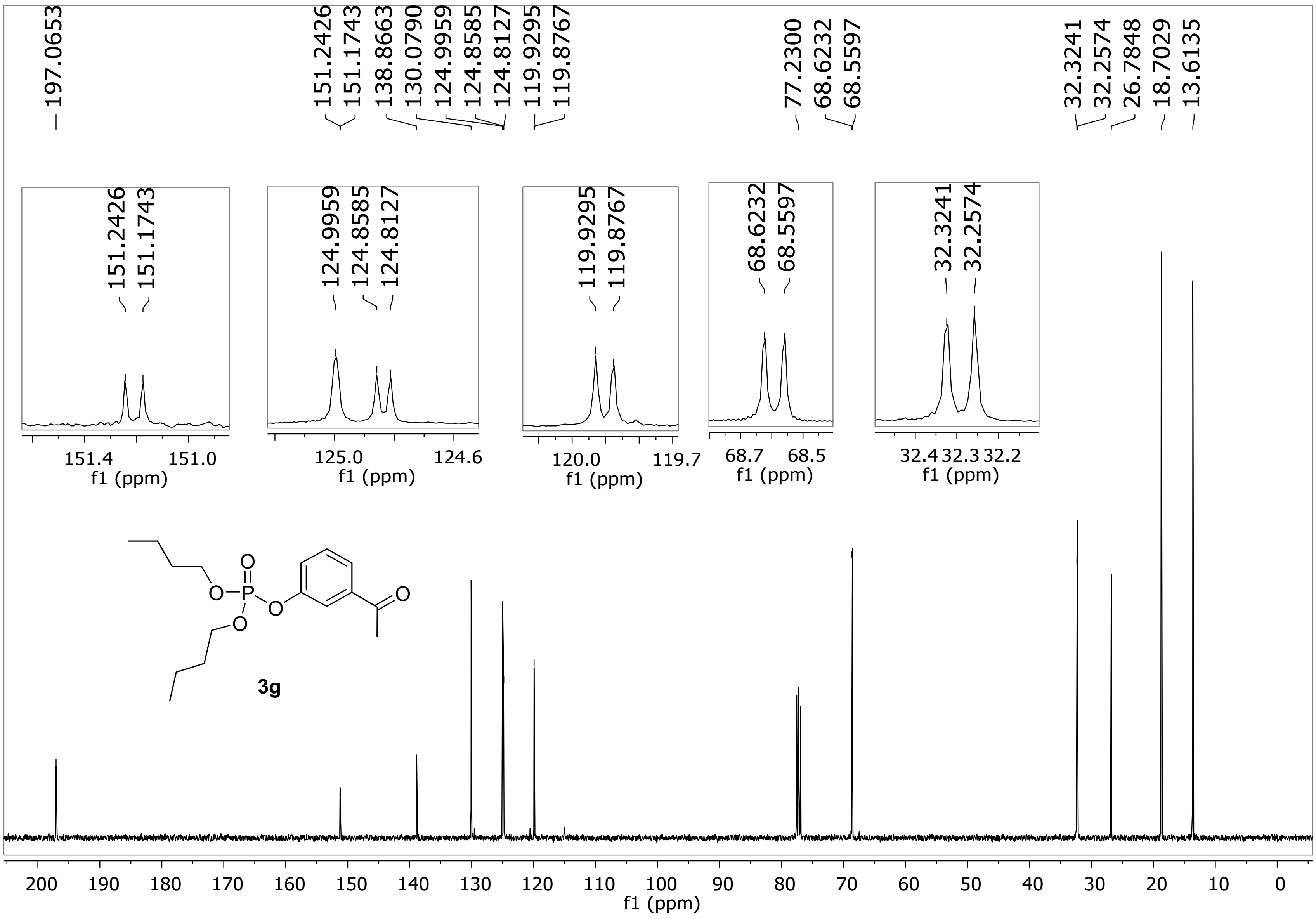

Figure S22. $^{13}C\{^{1}H\}$ NMR (100 MHz, $CDCl_3$) spectrum of compound **3g**

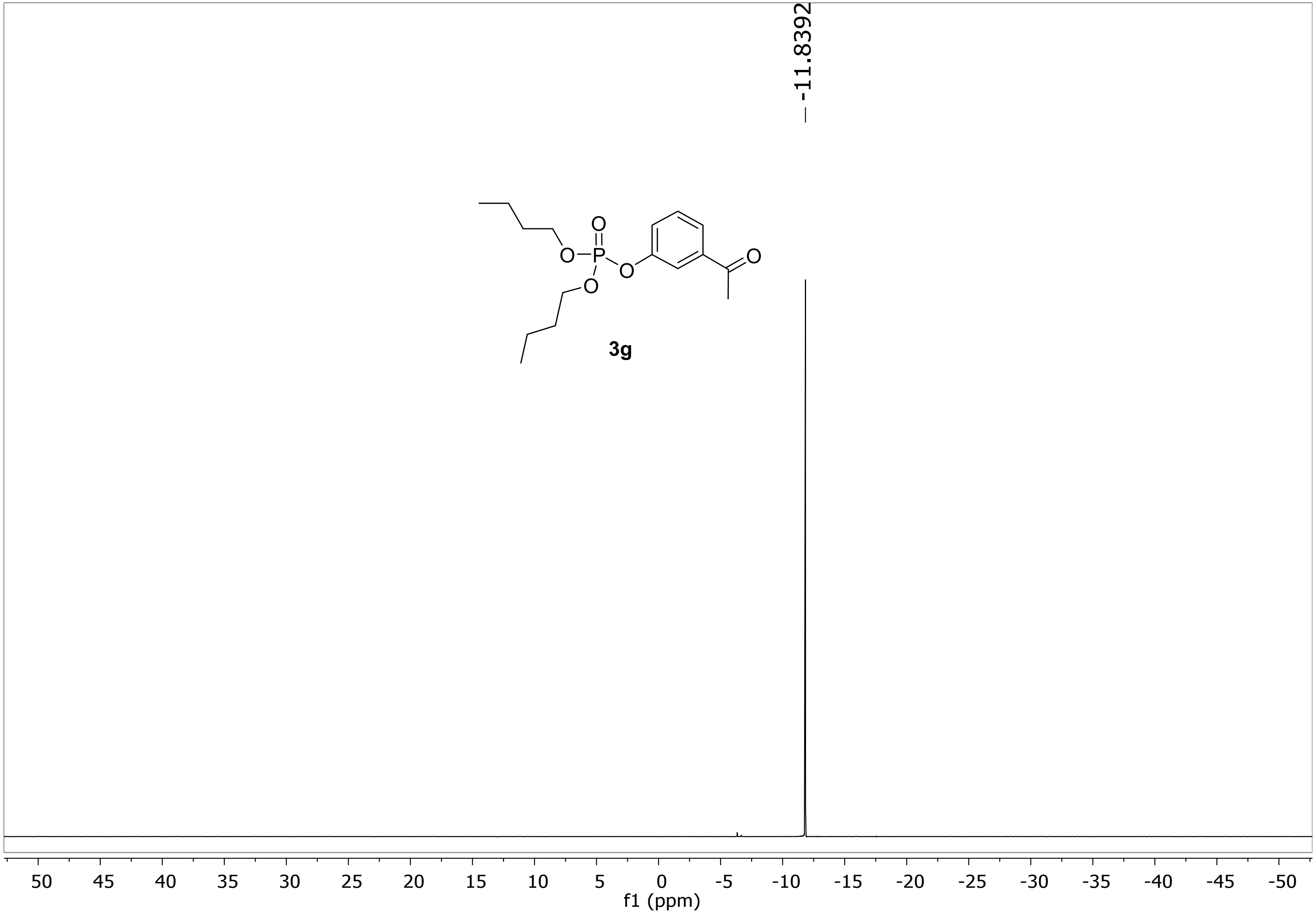

Figure S23. $^{31}P\{^{1}H\}$ NMR (162 MHz, $CDCl_3$) spectrum of compound **3g**

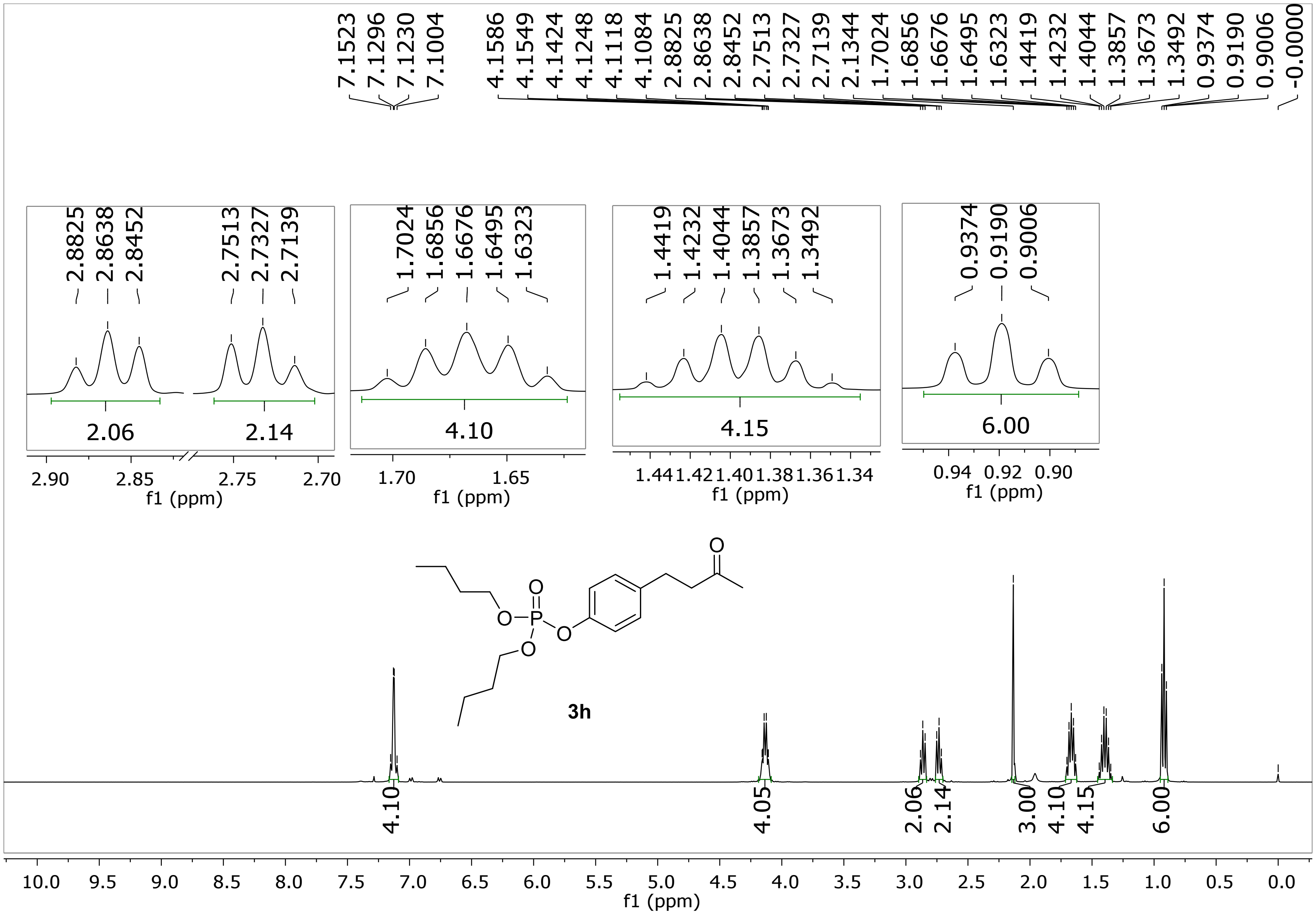

Figure S24. ^{1}H NMR (400 MHz, $CDCl_3$) spectrum of compound **3h**

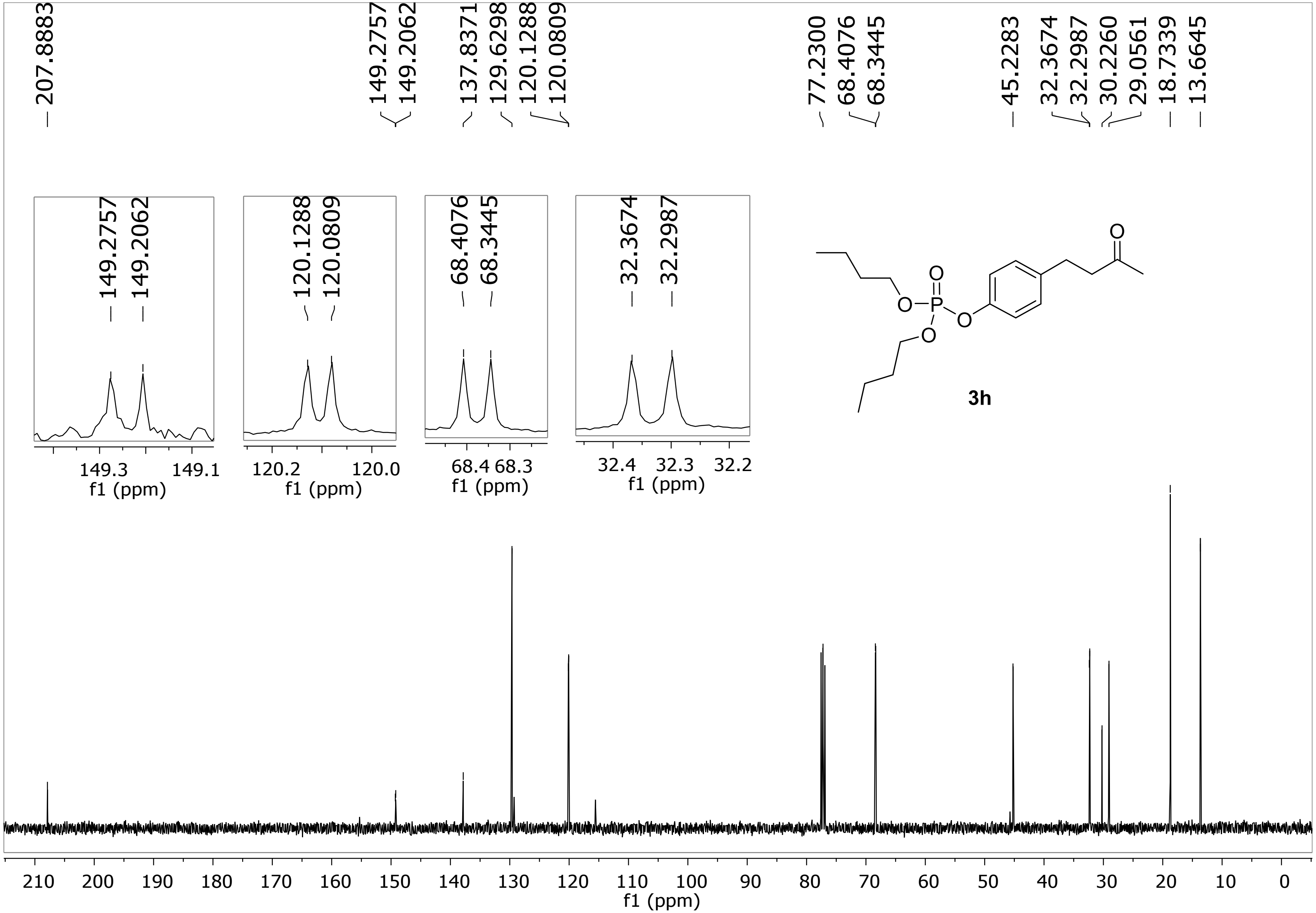

Figure S25. $^{13}C\{^1H\}$ NMR (100 MHz, $CDCl_3$) spectrum of compound **3h**

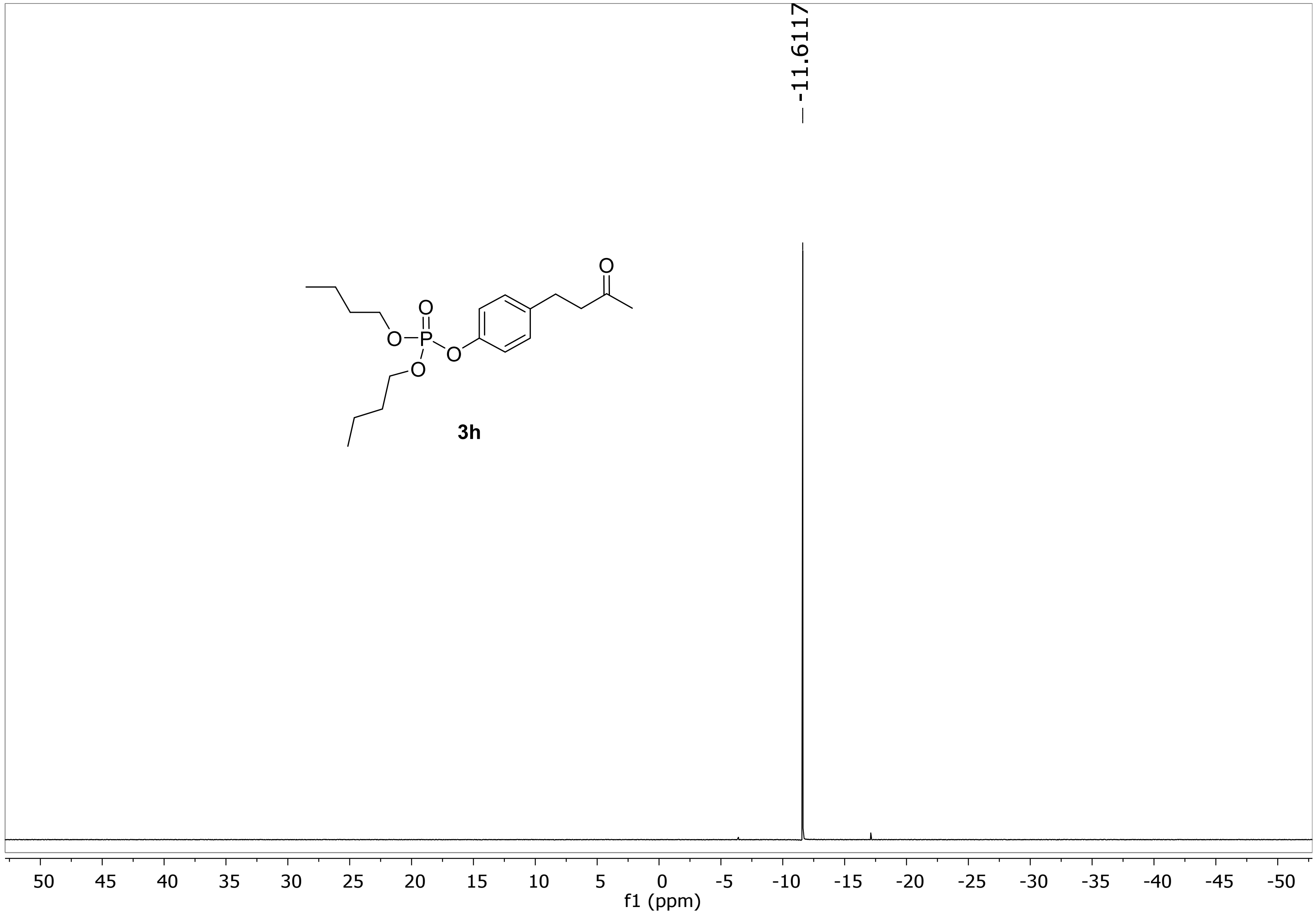

Figure S26. $^{31}P\{^{1}H\}$ NMR (162 MHz, $CDCl_3$) spectrum of compound **3h**

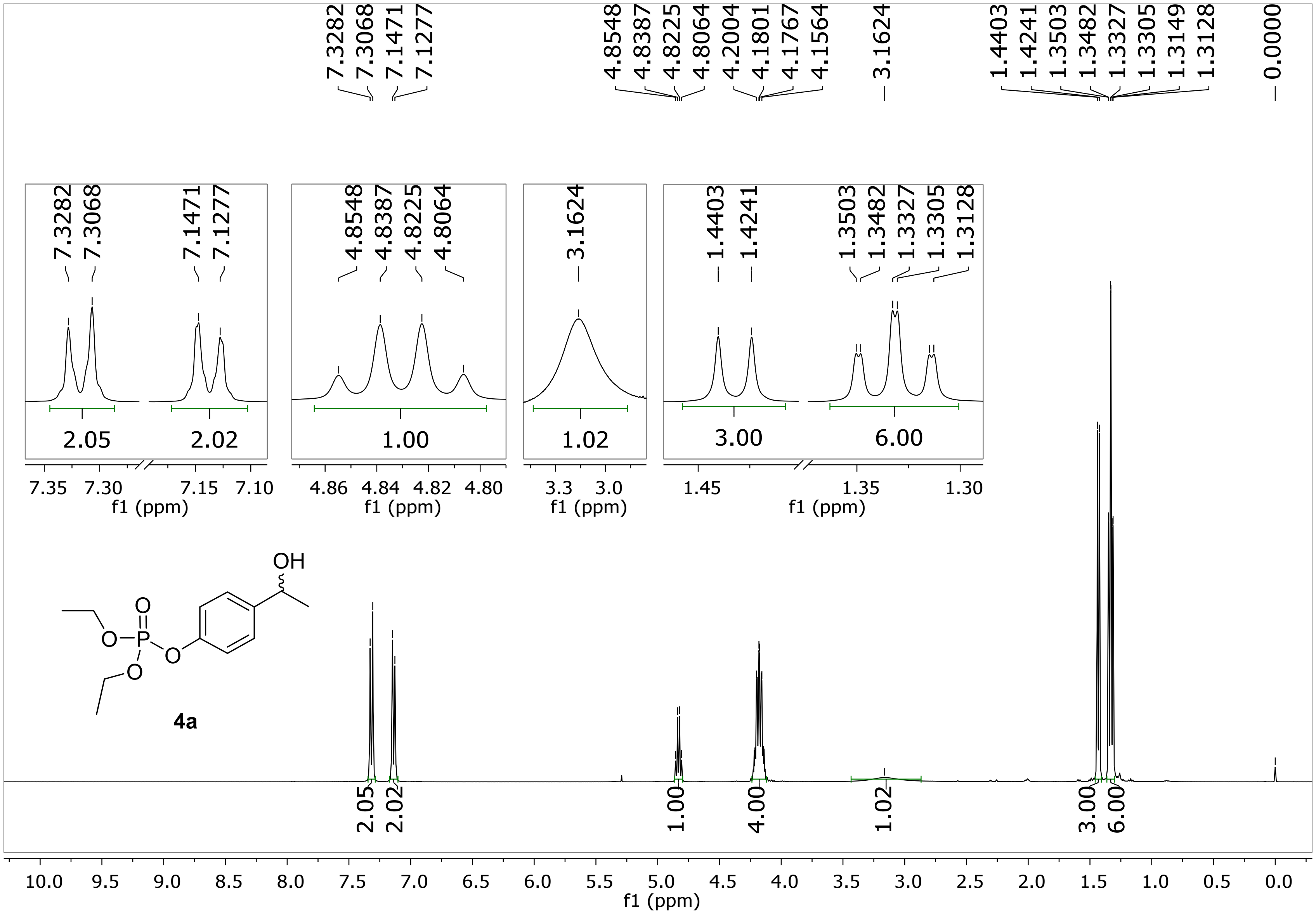

Figura S27. Espectro de RMN de ^{1}H do composto **4a** (400 MHz, $CDCl_3$)

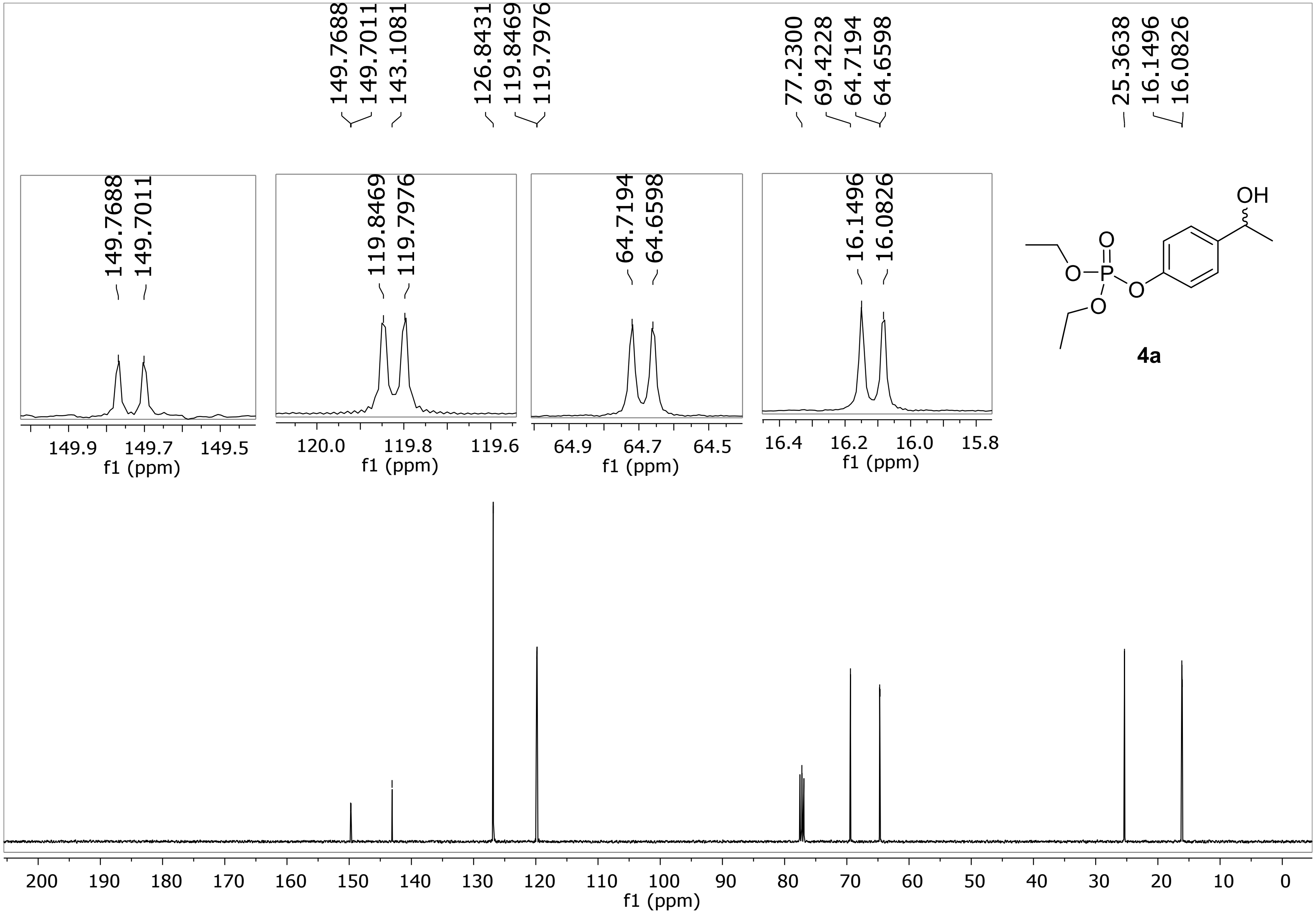

Figure S28. $^{13}C\{^1H\}$ NMR (100 MHz, $CDCl_3$) spectrum of compound **4a**

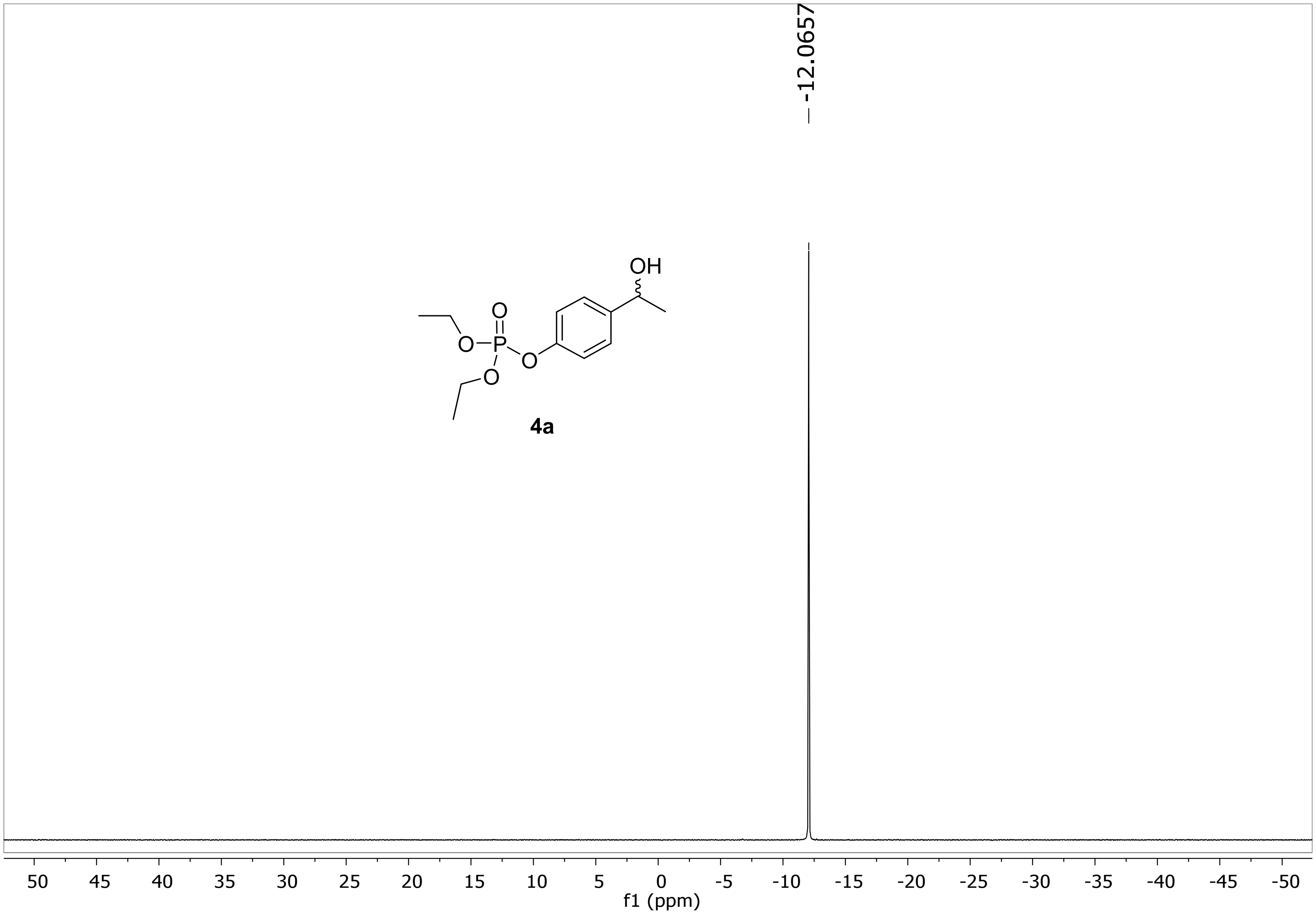

Figure S29. $^{31}P\{^{1}H\}$ NMR (162 MHz, $CDCl_3$) spectrum of compound **4a**

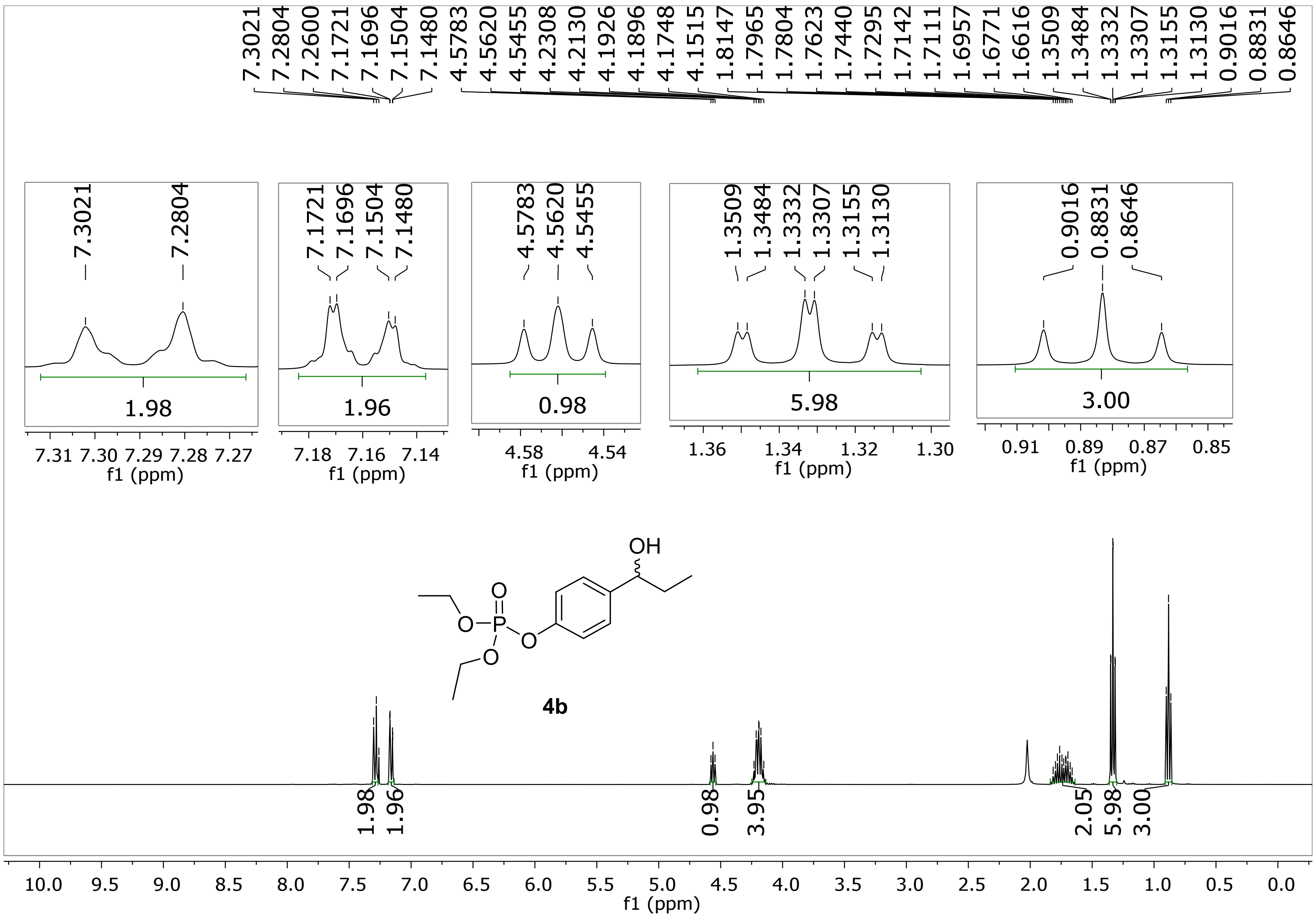

Figure S30. ^{1}H NMR (400 MHz, $CDCl_3$) spectrum of compound **4b**

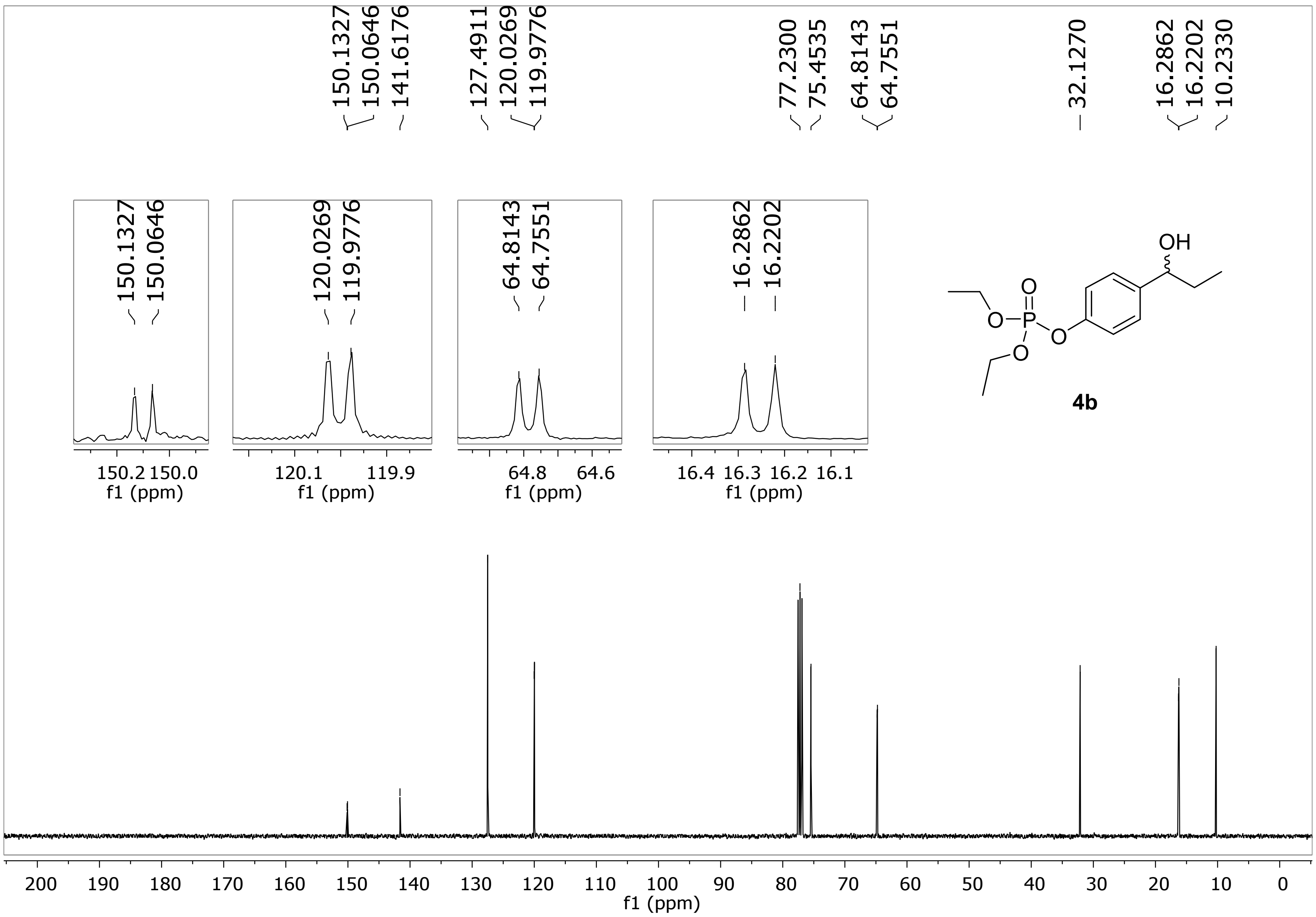

Figure S31. $^{13}C\{^{1}H\}$ NMR (100 MHz, $CDCl_3$) spectrum of compound **4b**

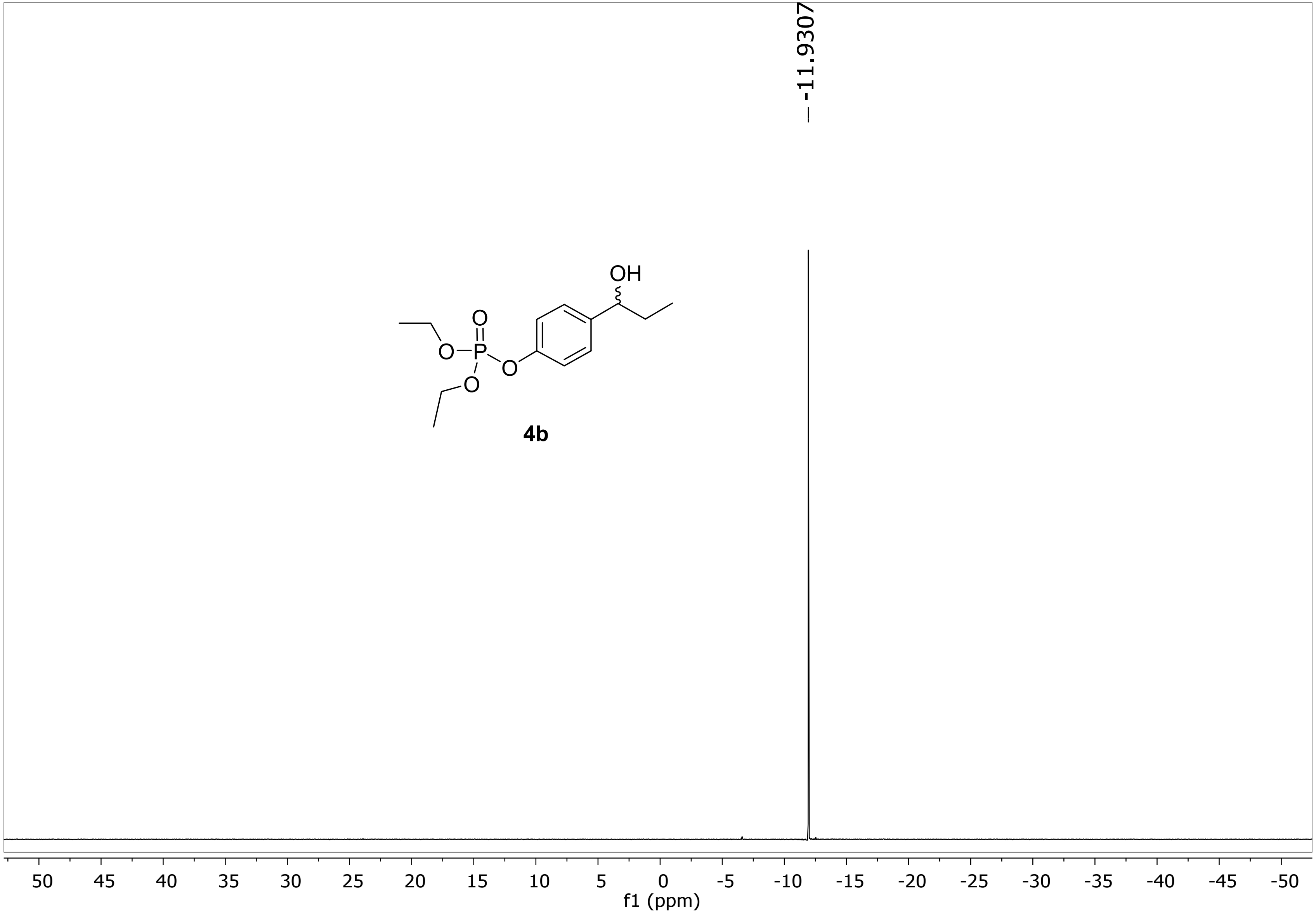

Figure S32. $^{31}P\{^{1}H\}$ NMR (162 MHz, $CDCl_3$) spectrum of compound **4b**

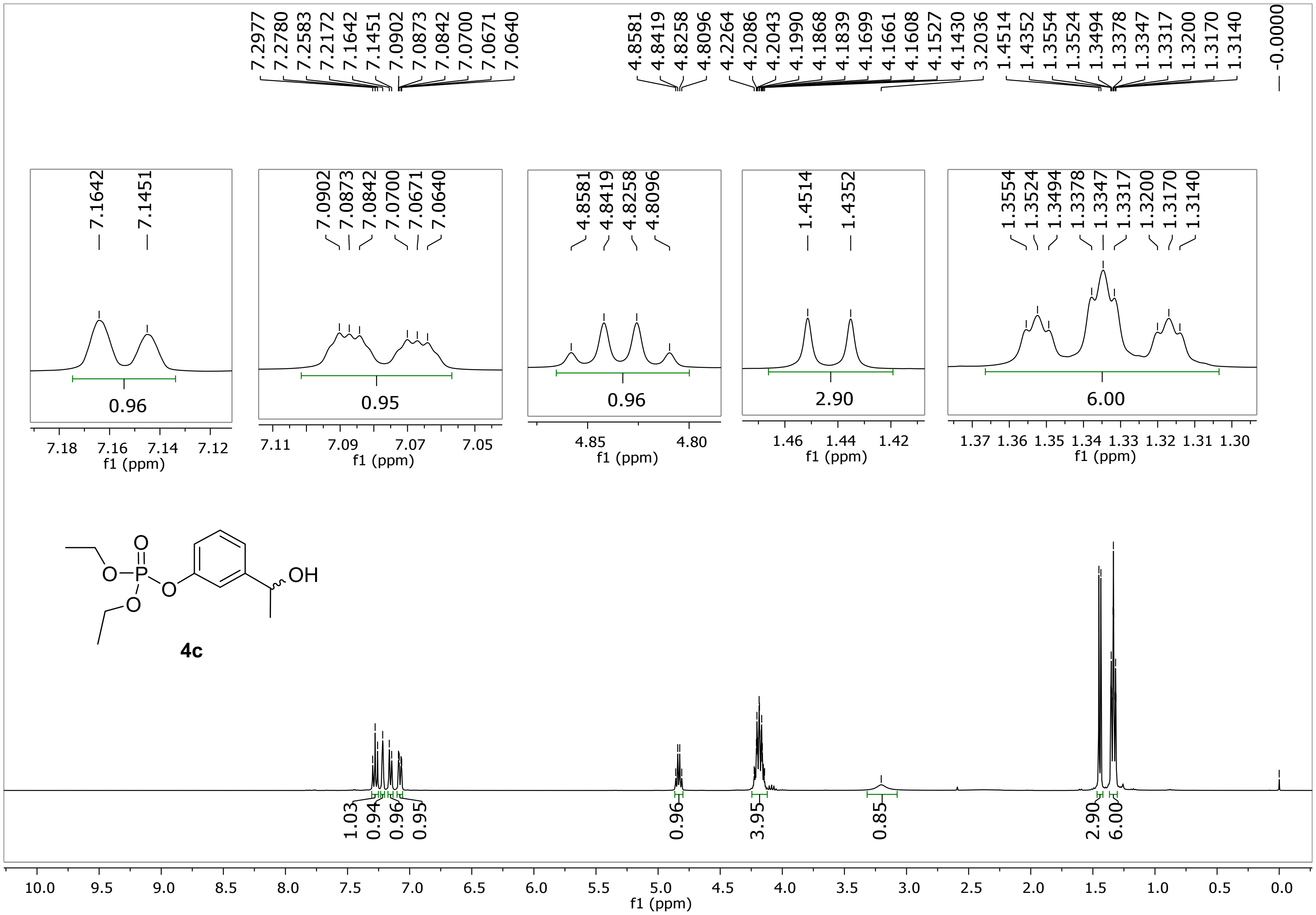

Figure S33. ^{1}H NMR (400 MHz, $CDCl_3$) spectrum of compound **4c**

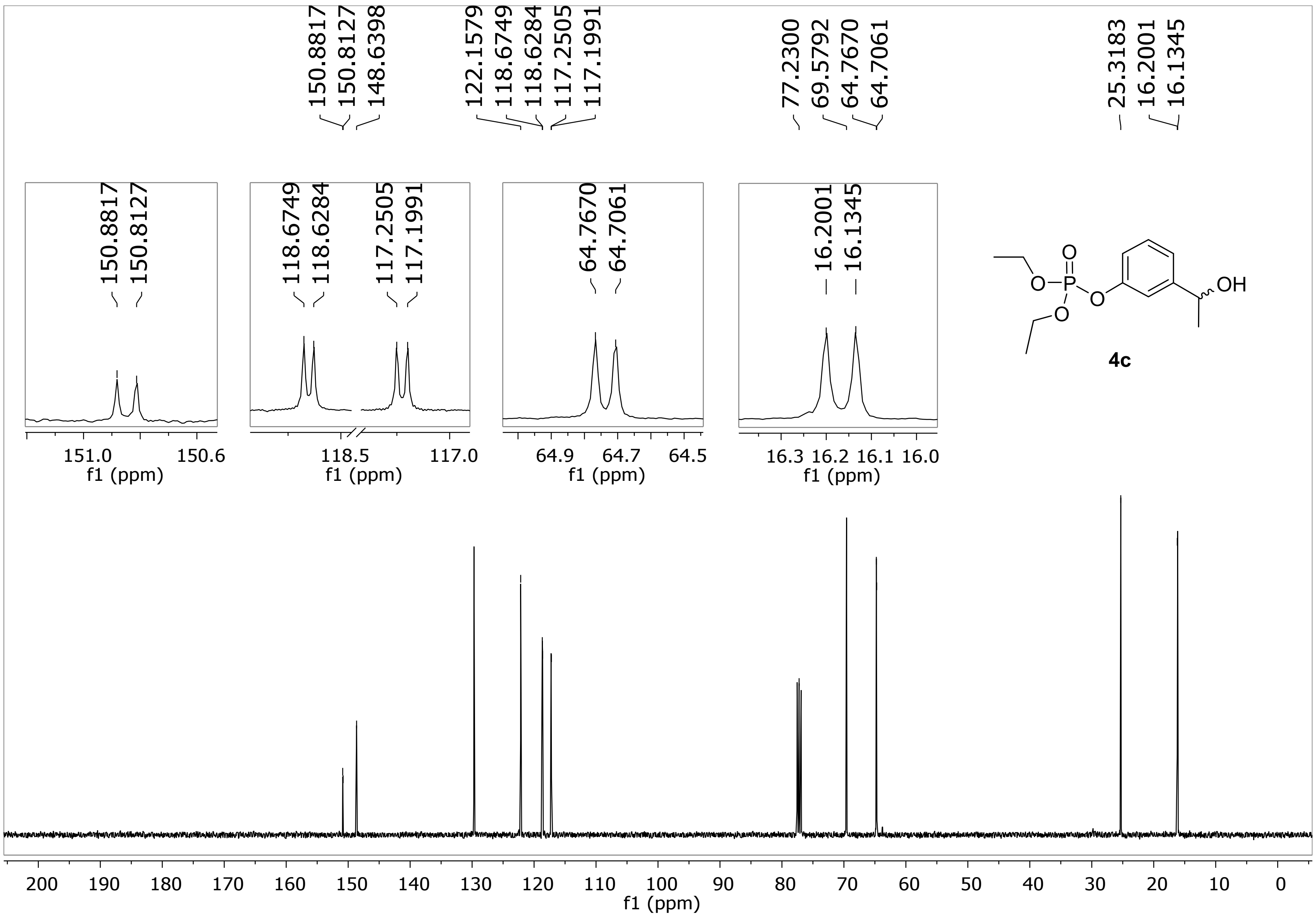

Figure S34. $^{13}C\{^{1}H\}$ NMR (100 MHz, $CDCl_3$) spectrum of compound **4c**

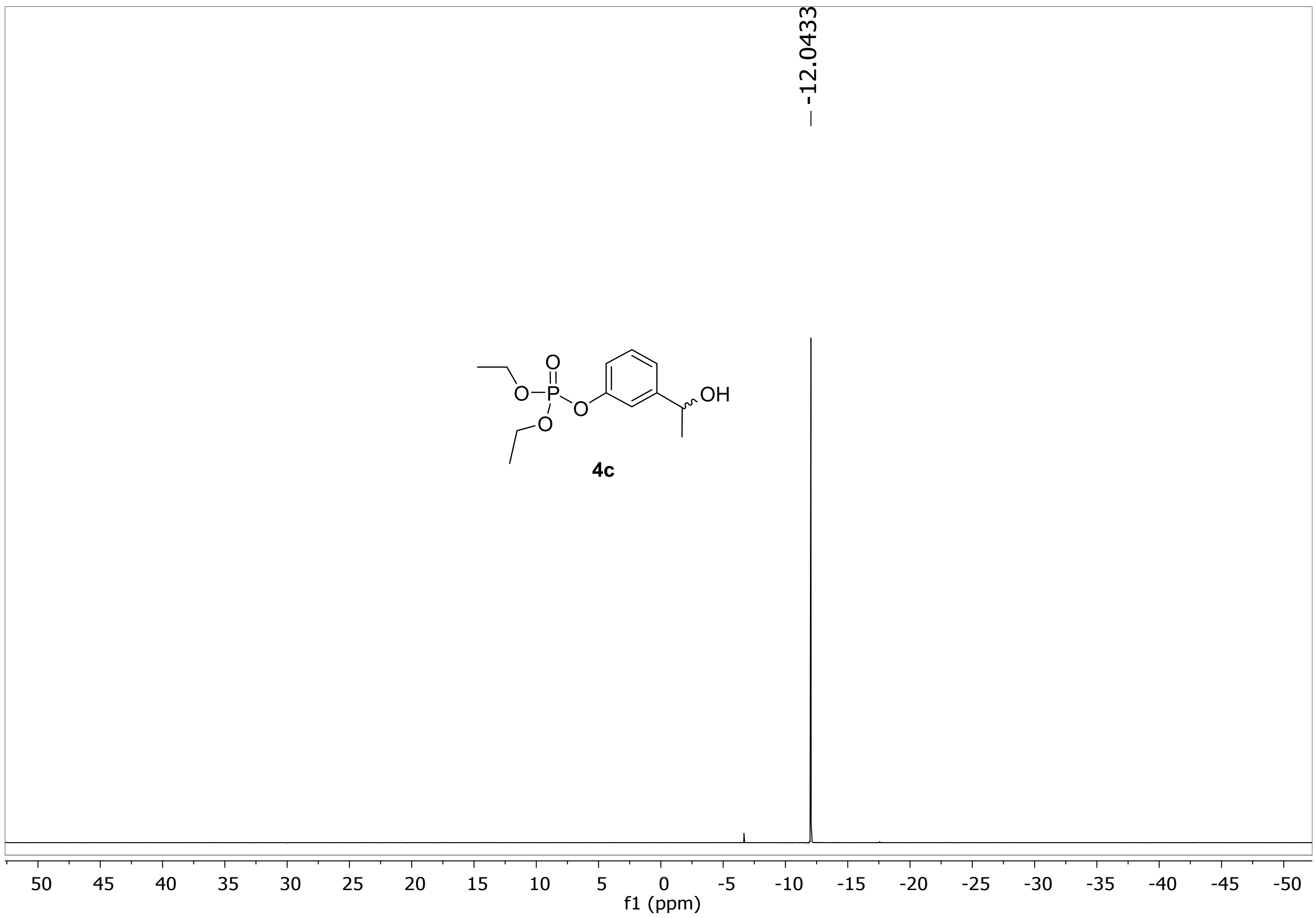

Figure S35. $^{31}P\{^1H\}$ NMR (162 MHz, $CDCl_3$) spectrum of compound **4c**

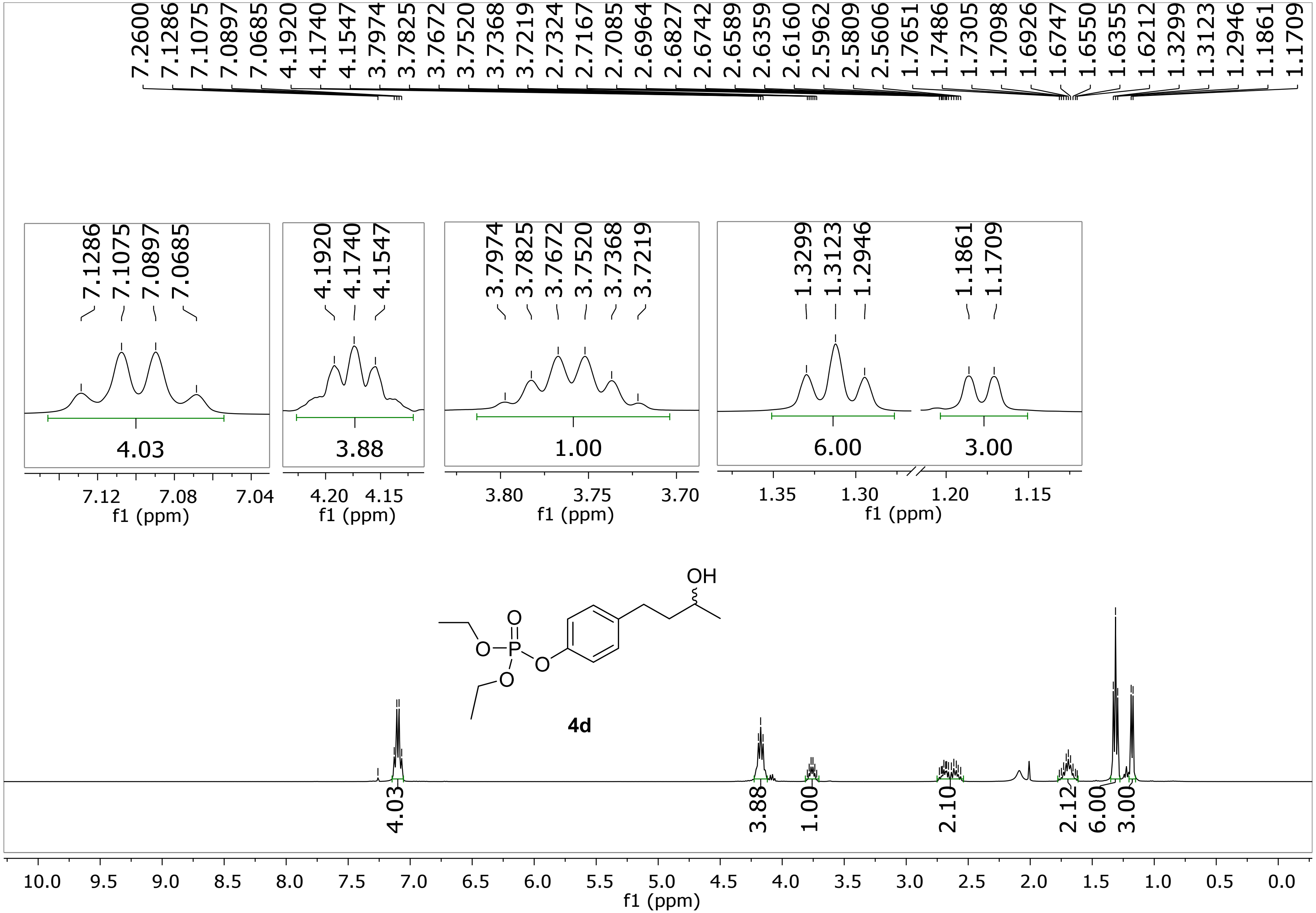

Figure S36 ^{1}H NMR (400 MHz, $CDCl_3$) spectrum of compound **4d**

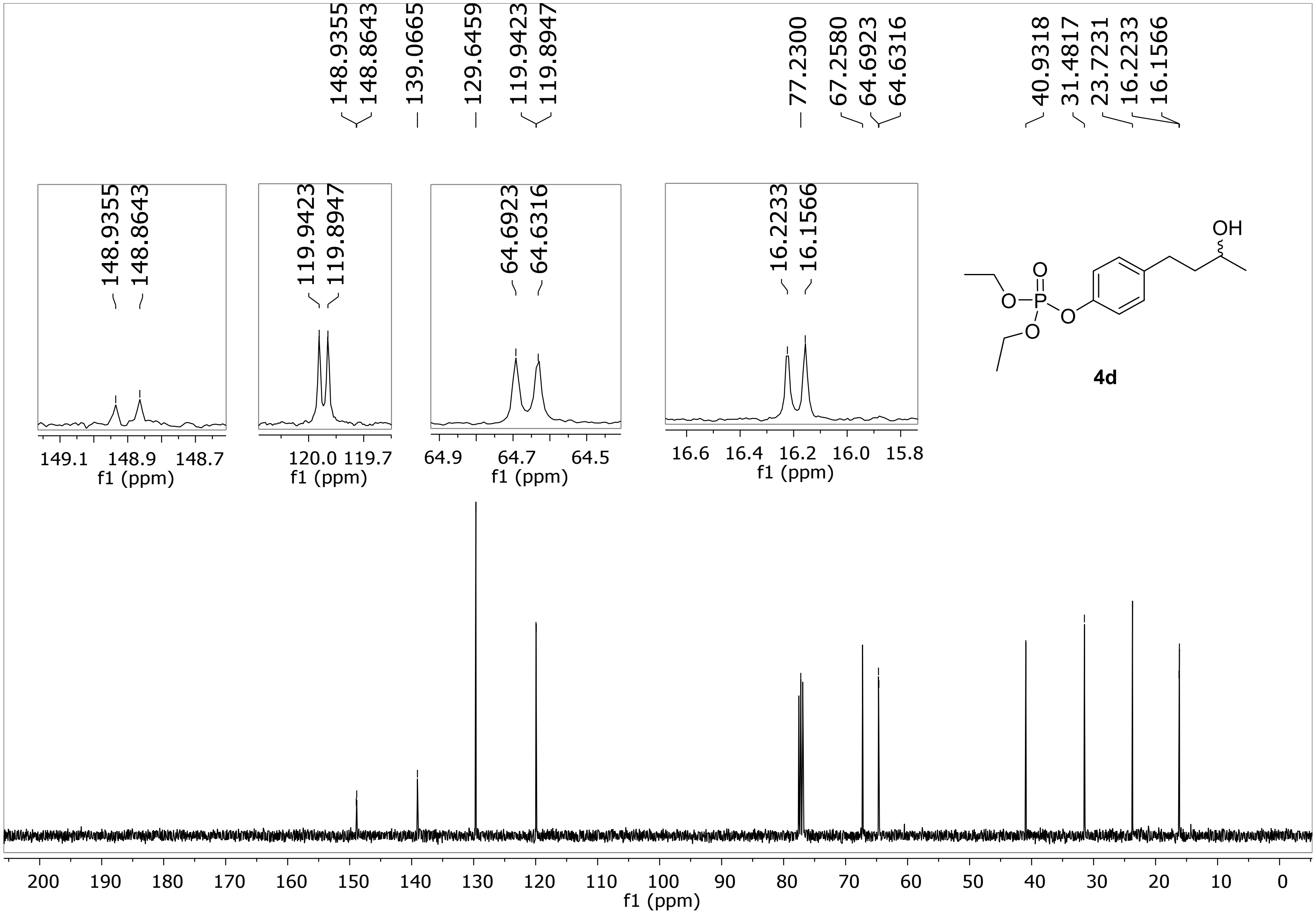

Figure S37. $^{13}C\{^{1}H\}$ NMR (100 MHz, $CDCl_3$) spectrum of compound **4d**

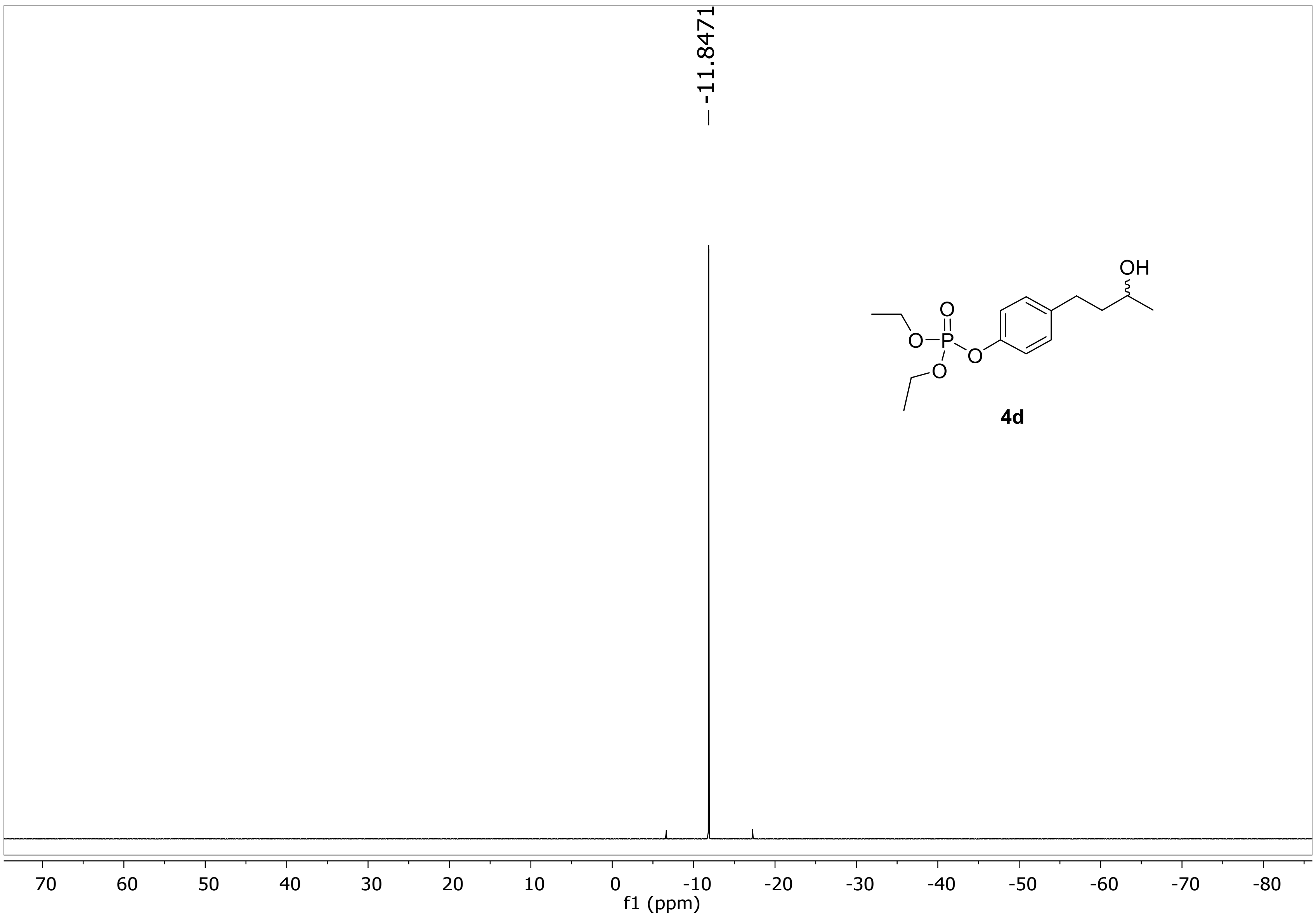

Figure S38. $^{31}P\{^{1}H\}$ NMR (162 MHz, $CDCl_3$) spectrum of compound **4d**

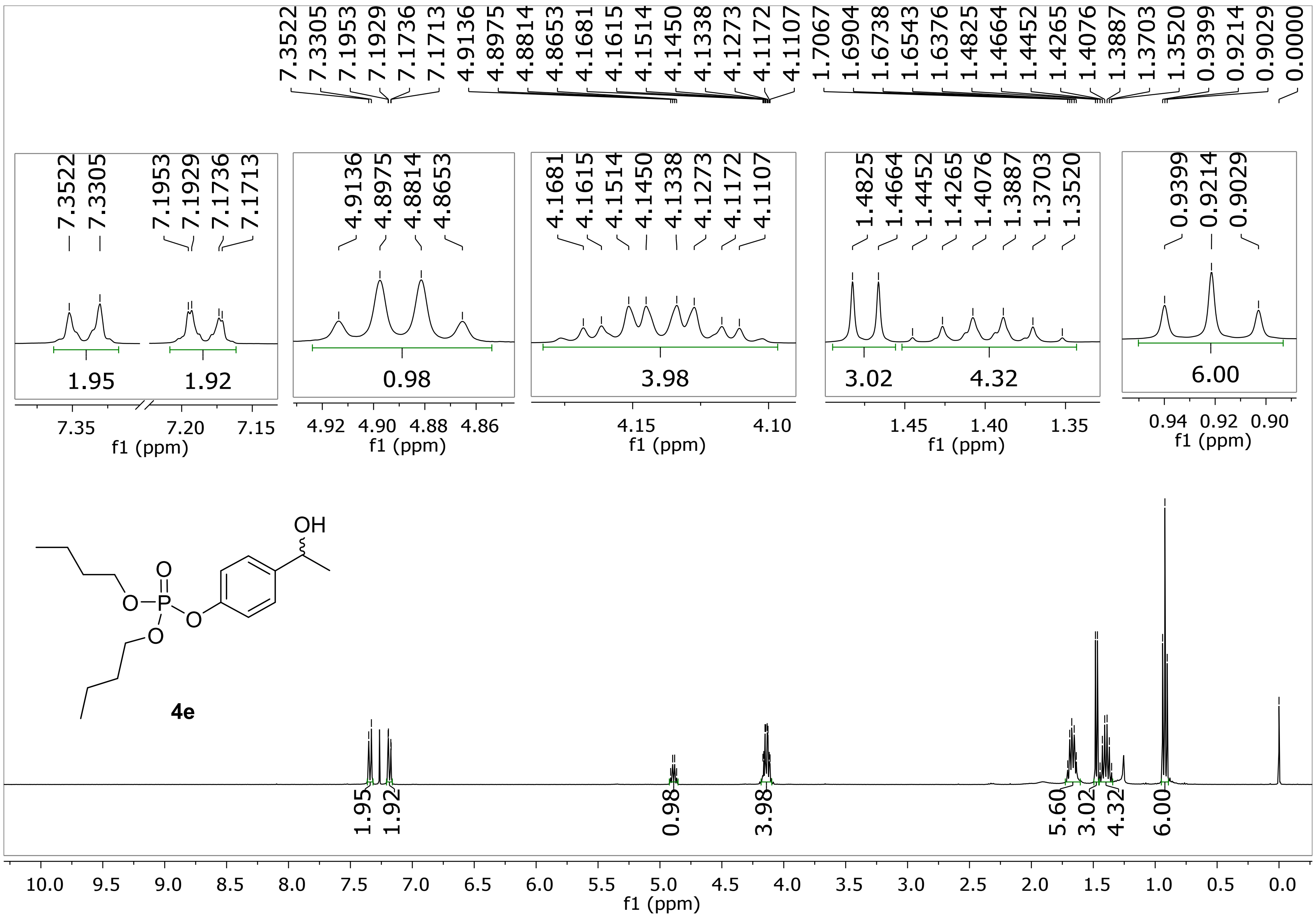

Figure S39. ^{1}H NMR (400 MHz, $CDCl_3$) spectrum of compound **4e**

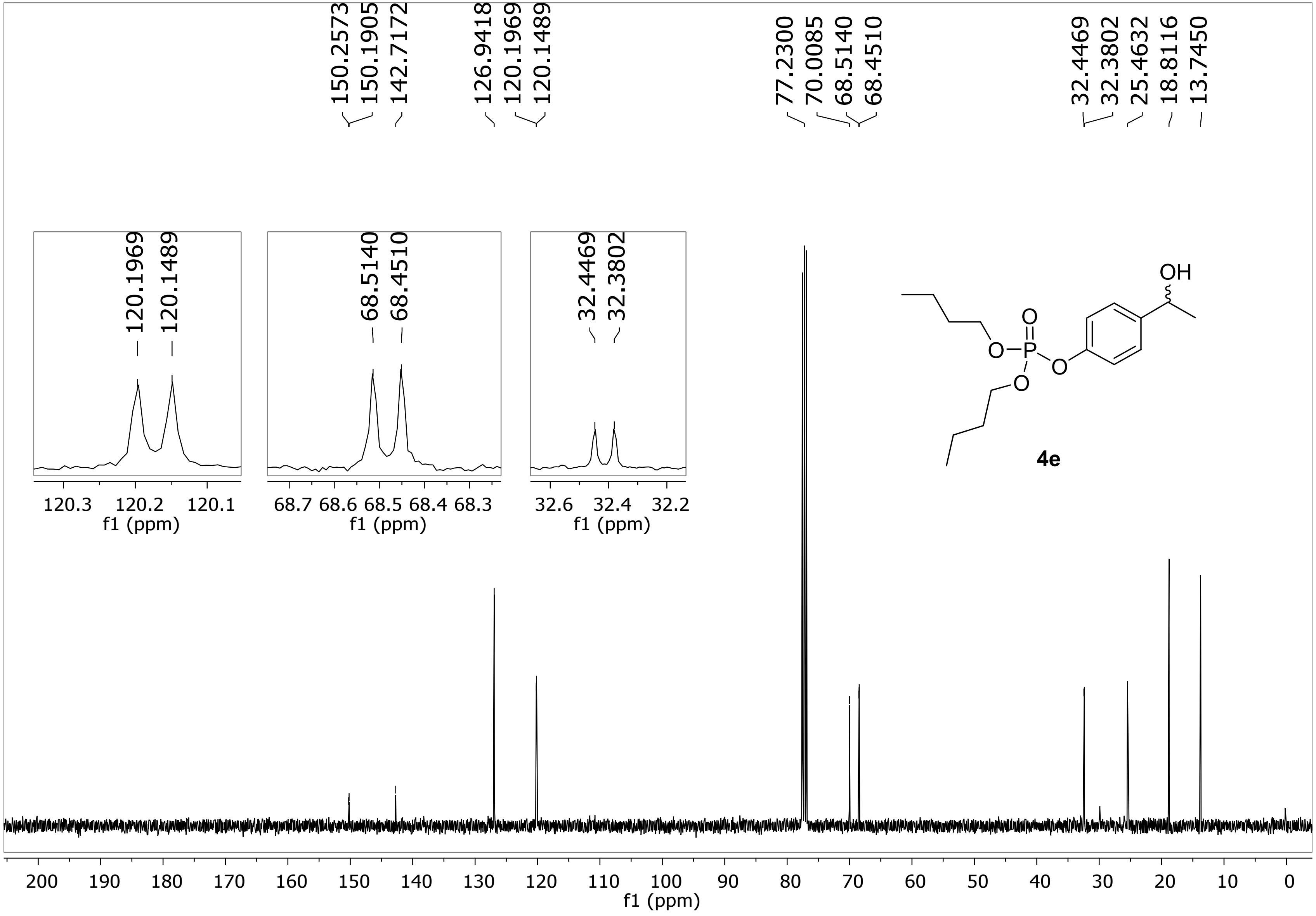

Figure S40. $^{13}C\{^1H\}$ NMR (100 MHz, $CDCl_3$) spectrum of compound **4e**

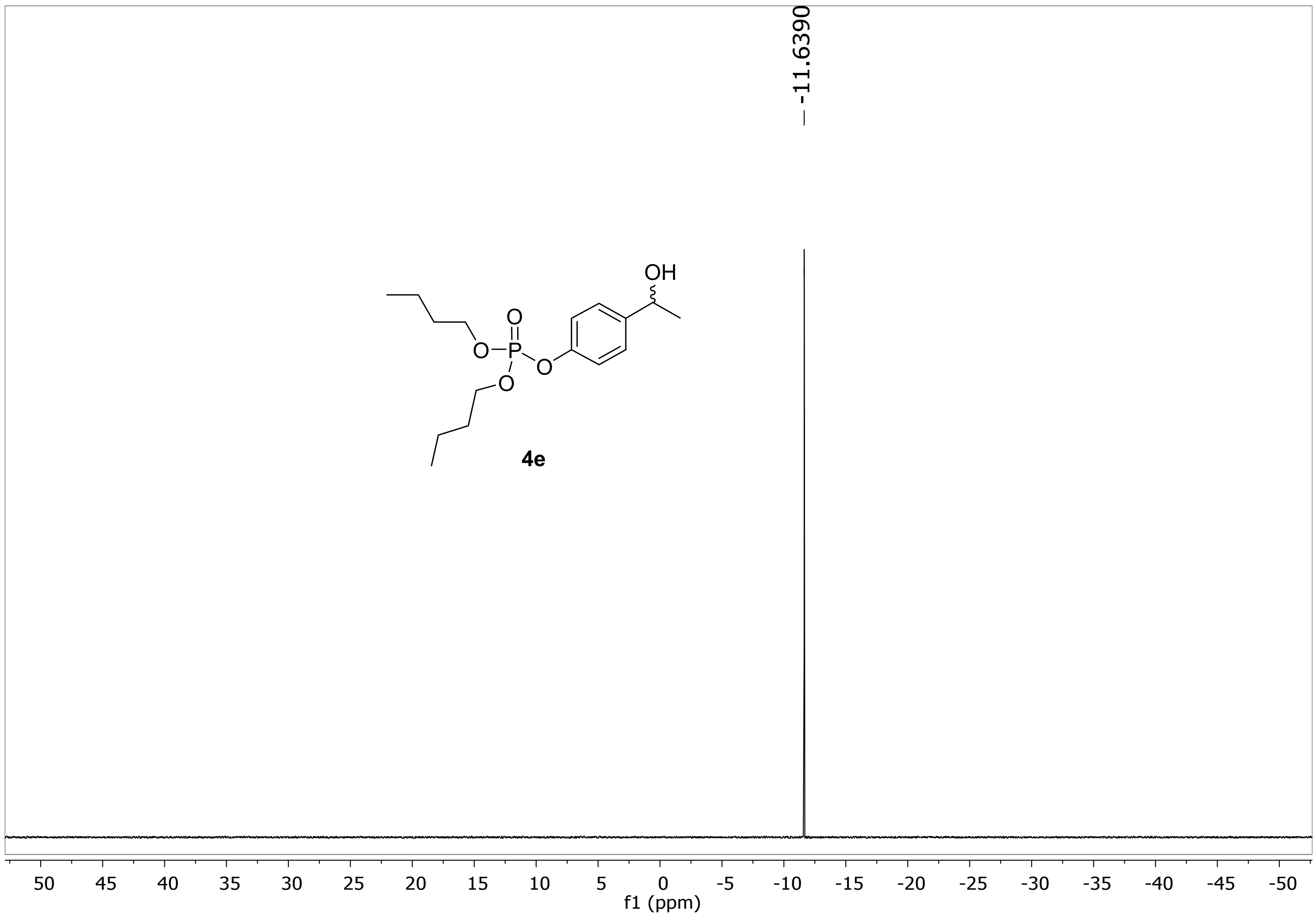

Figure S41. $^{31}P\{^{1}H\}$ NMR (162 MHz, $CDCl_3$) spectrum of compound **4e**

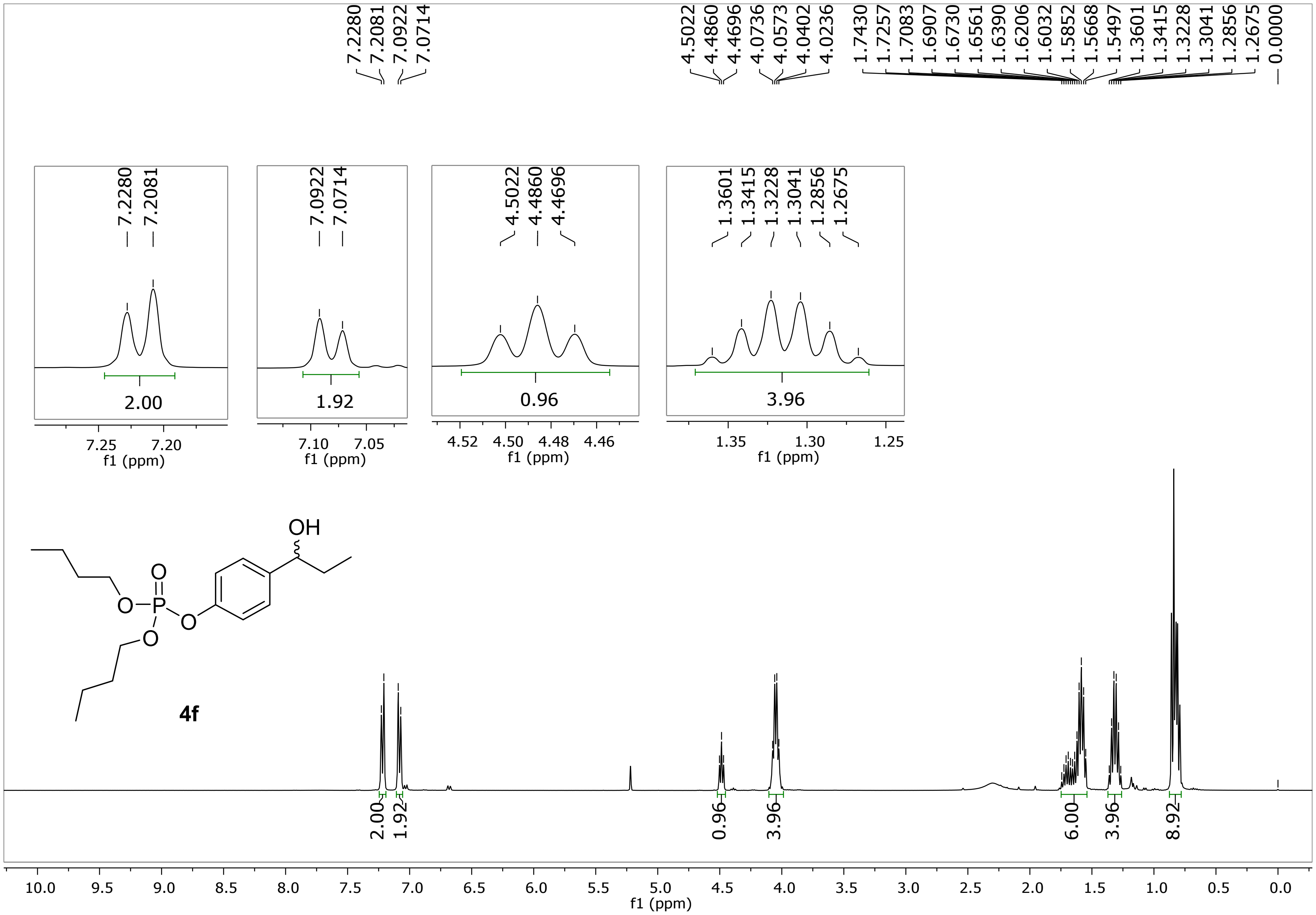

Figure S42. ^{1}H NMR (400 MHz, $CDCl_3$) spectrum of compound **4f**

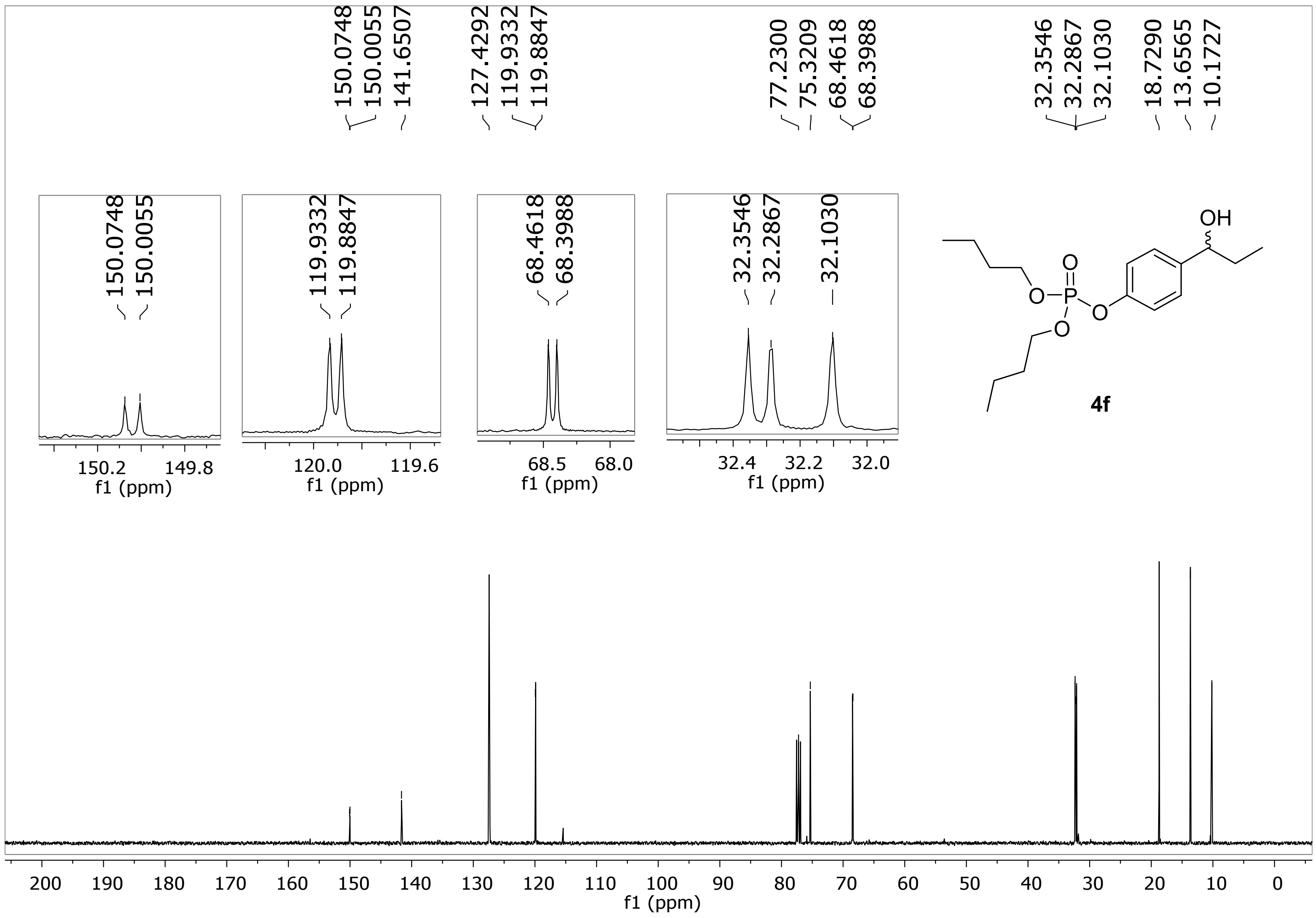

Figure S43. $^{13}C\{^{1}H\}$ NMR (100 MHz, $CDCl_3$) spectrum of compound **4f**

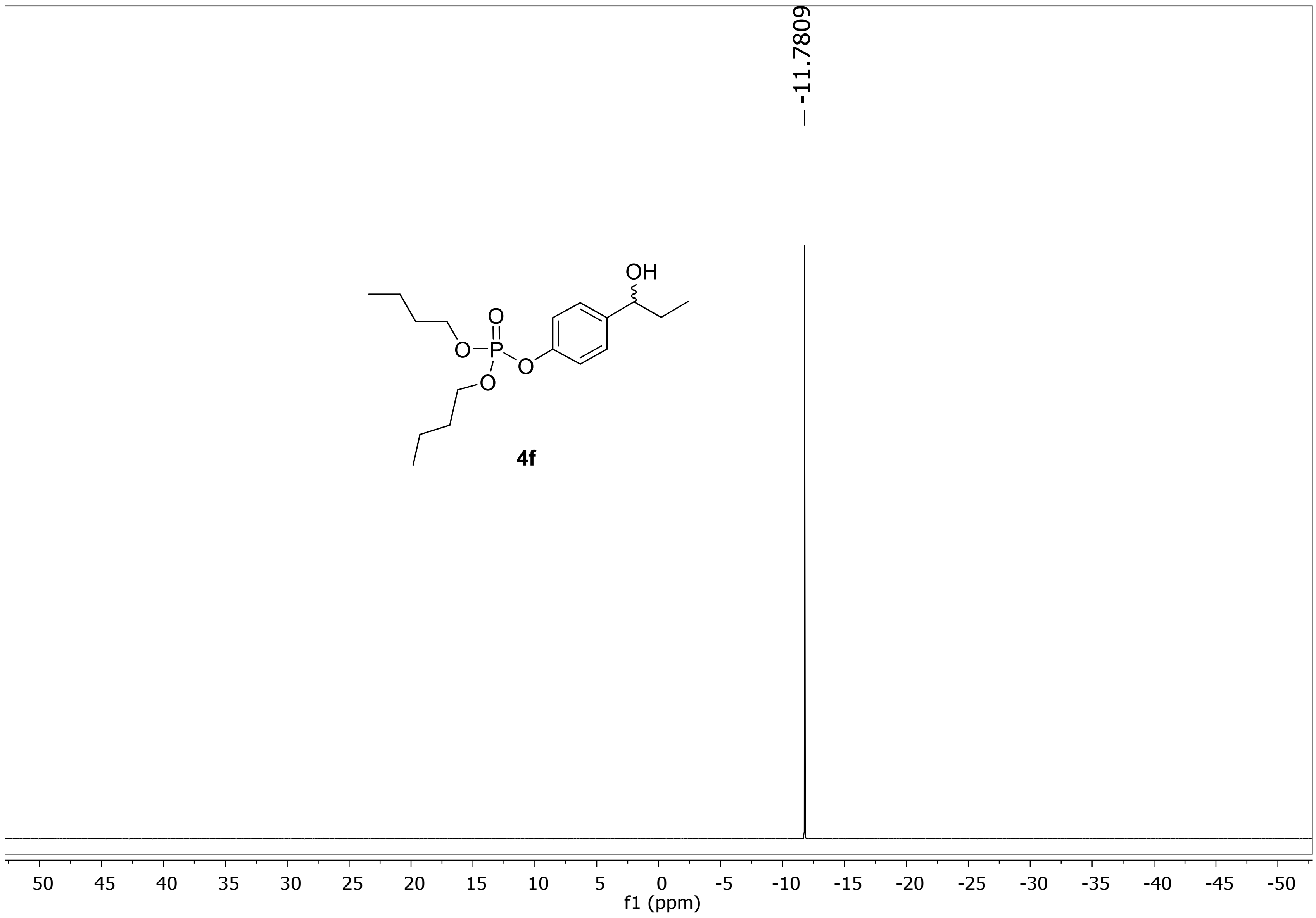

Figure S44. $^{31}P\{^{1}H\}$ NMR (162 MHz, $CDCl_3$) spectrum of compound **4f**

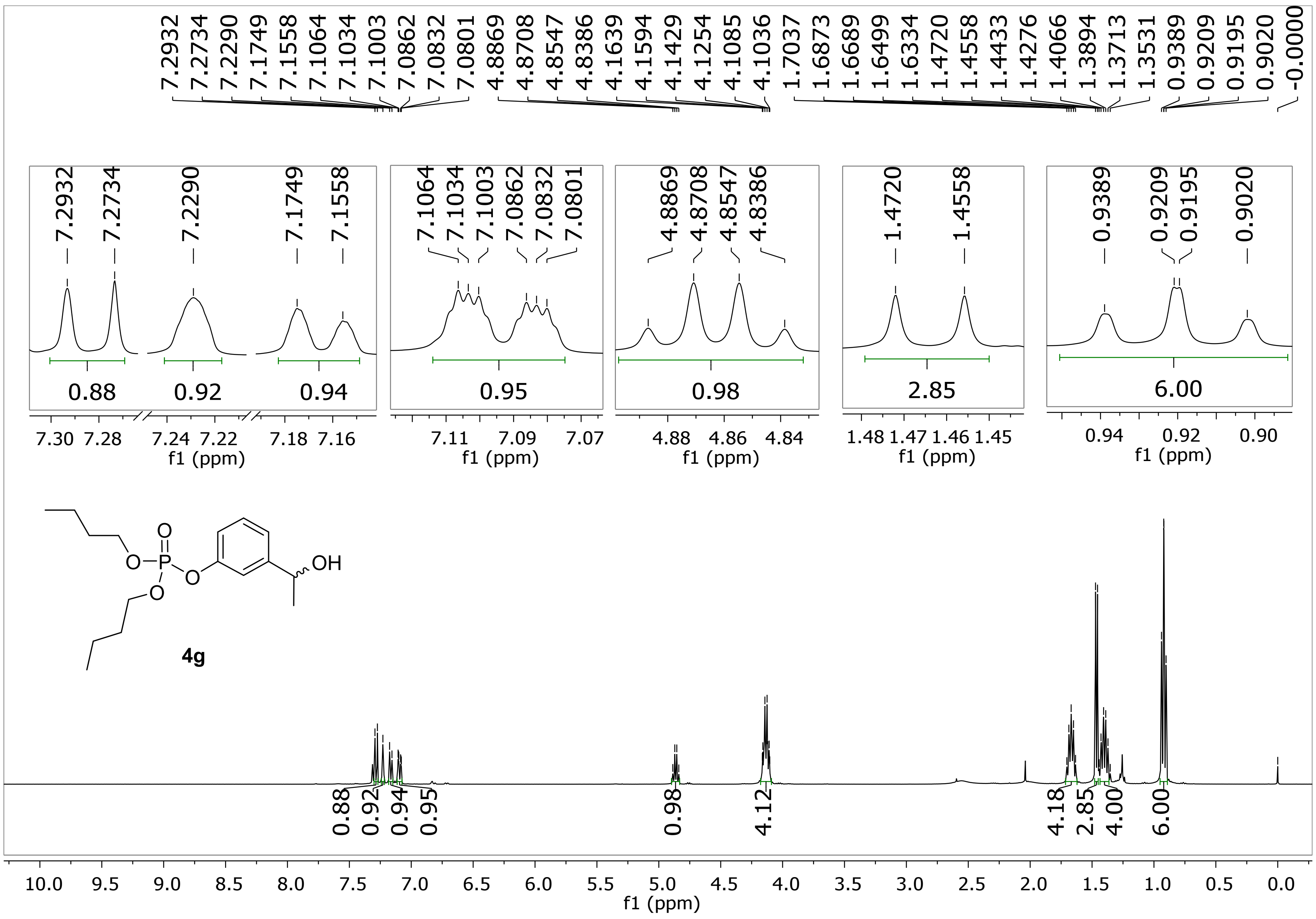

Figure S45. ^{1}H NMR (400 MHz, $CDCl_3$) spectrum of compound **4g**

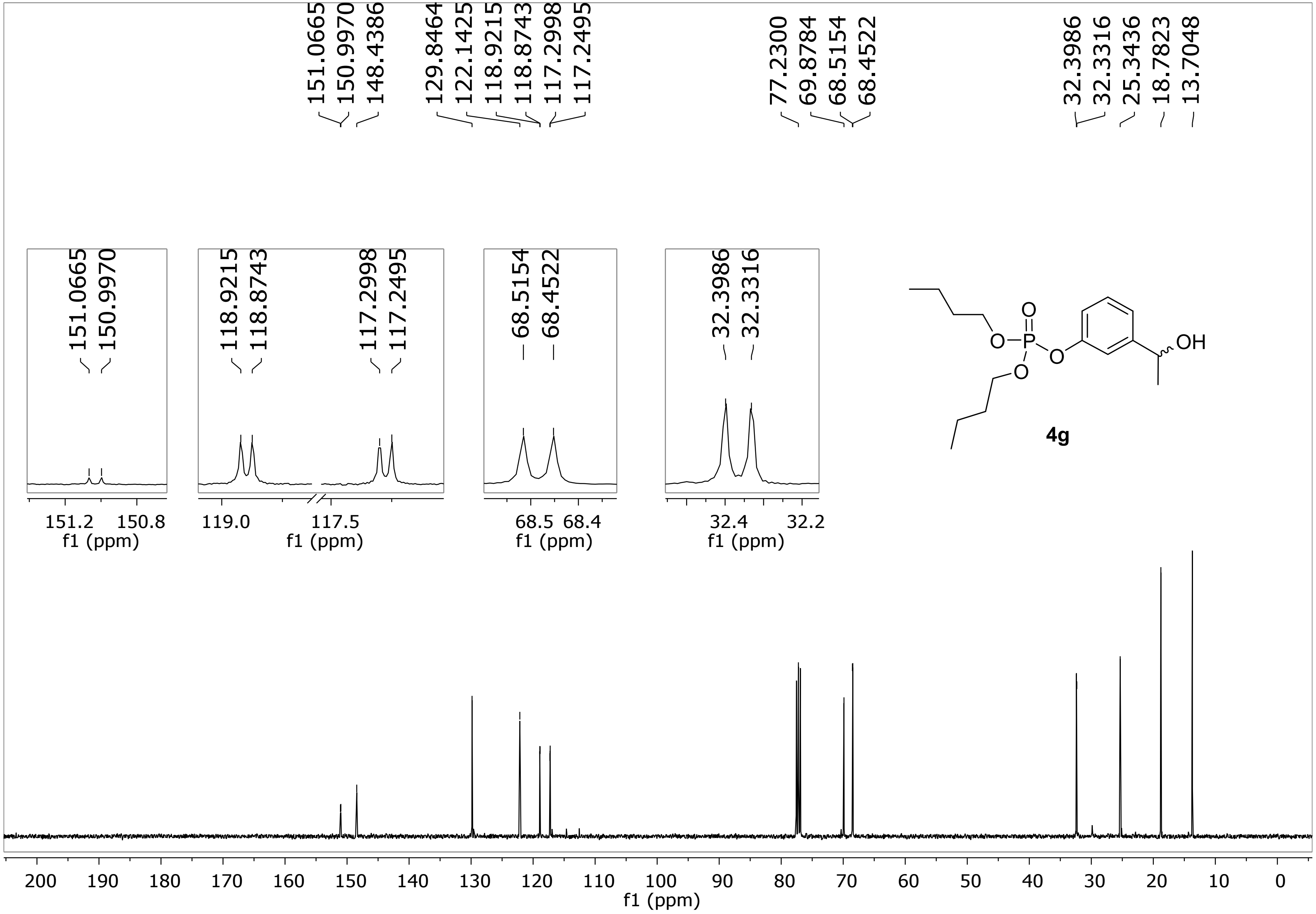

Figure S46. $^{13}C\{^1H\}$ NMR (100 MHz, $CDCl_3$) spectrum of compound **4g**

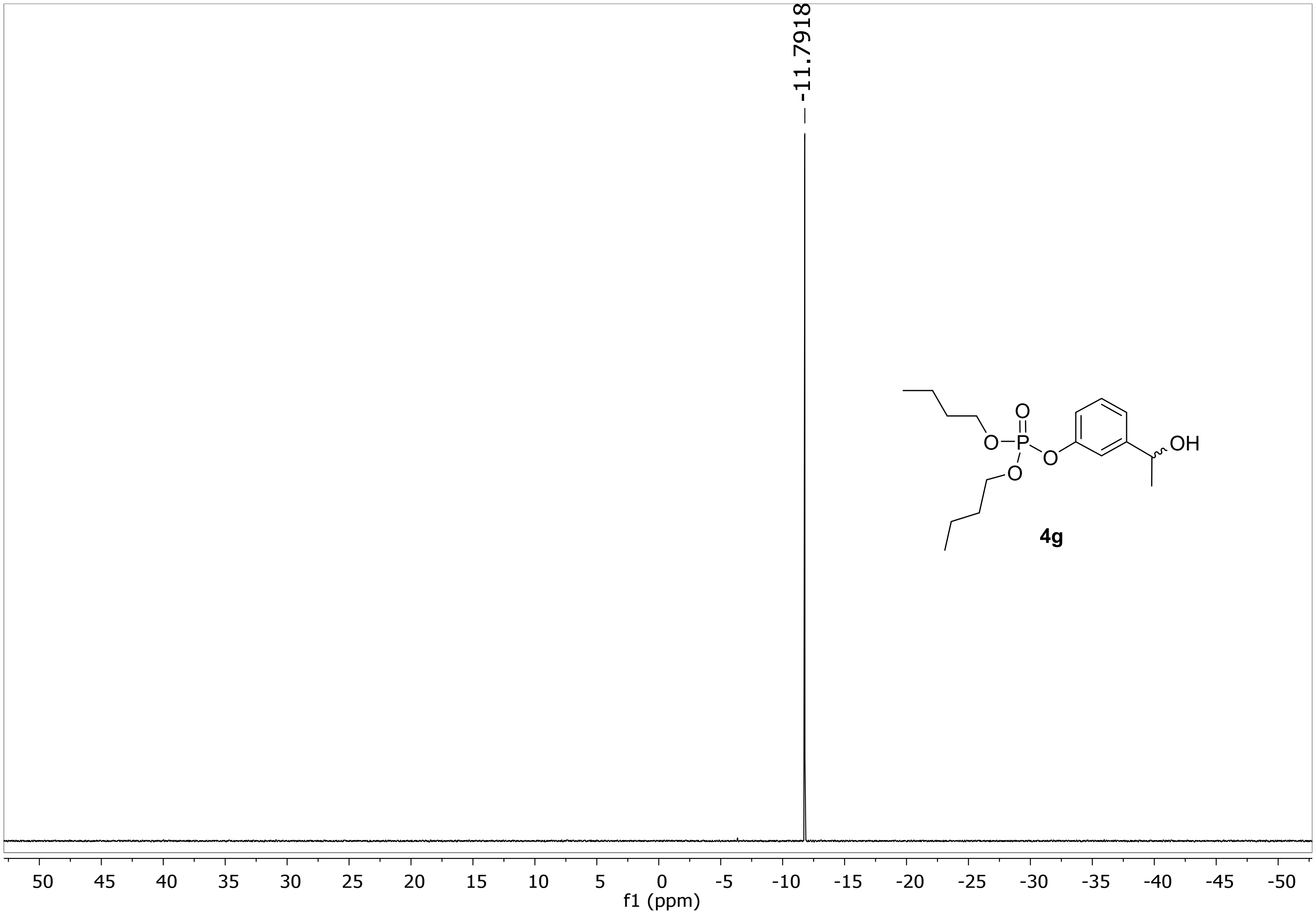

Figure S47. $^{31}P\{^{1}H\}$ NMR (162 MHz, $CDCl_3$) spectrum of compound **4g**

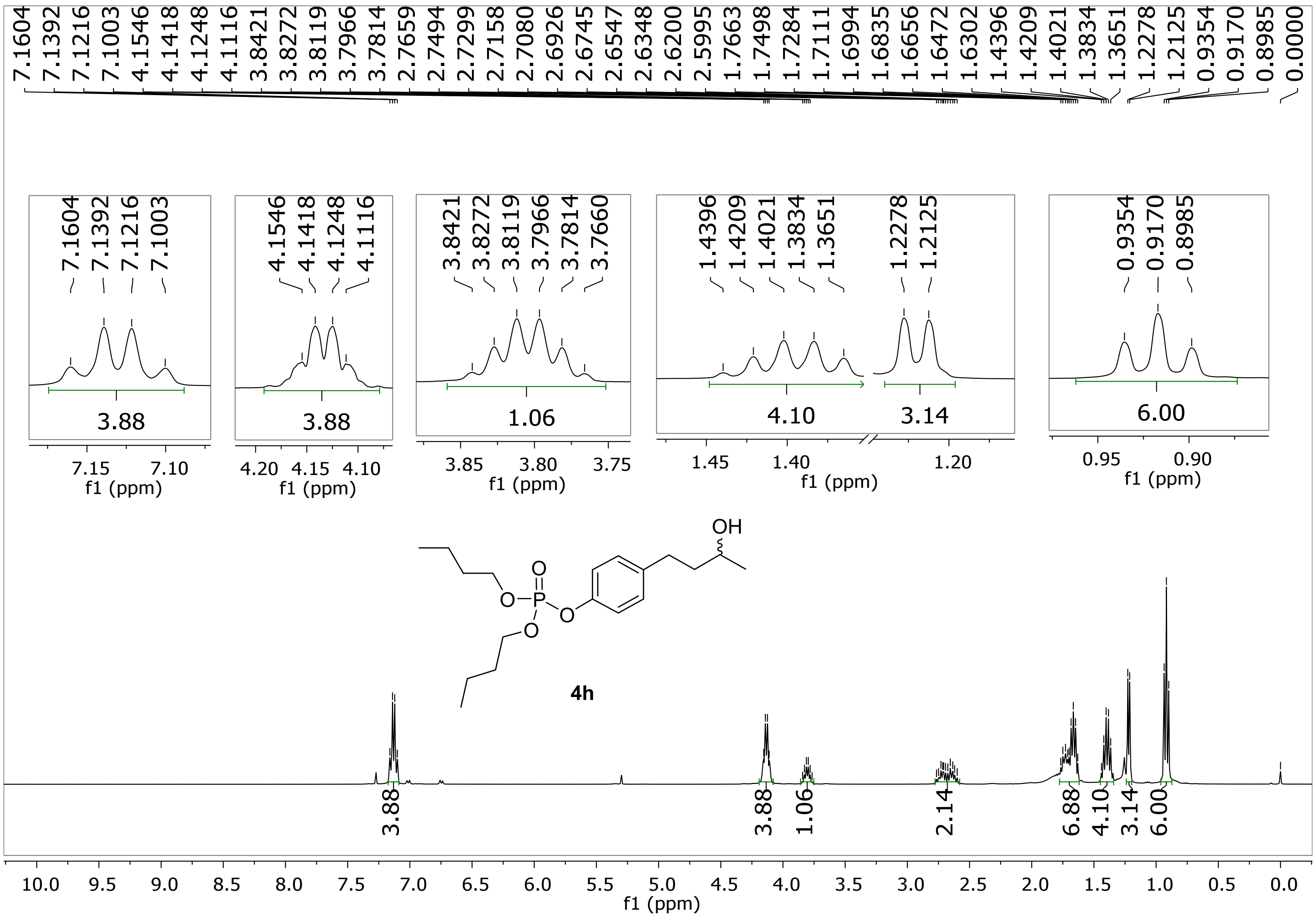

Figure S48. ^{1}H NMR (400 MHz, $CDCl_3$) spectrum of compound **4h**

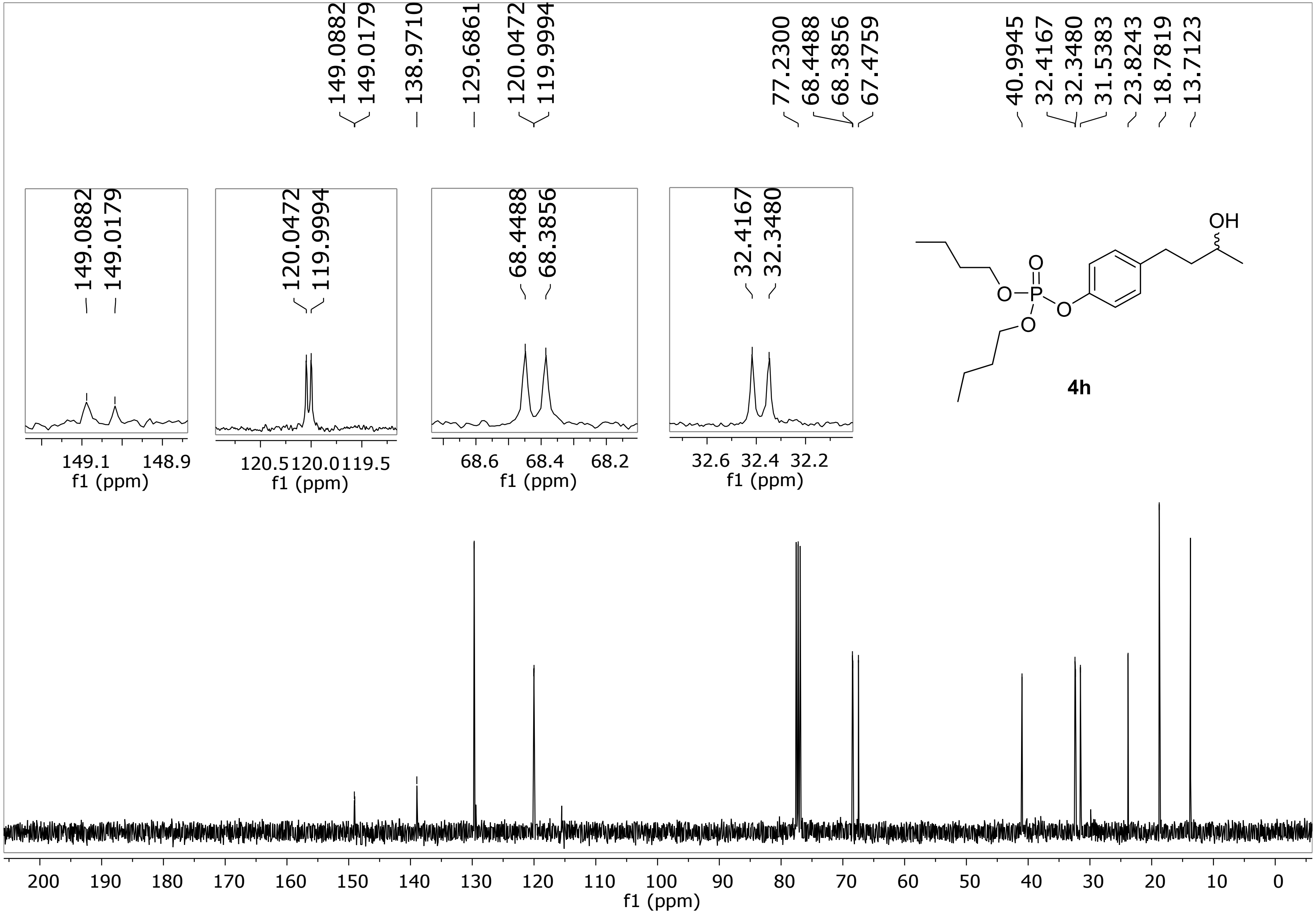

Figure S49. $^{13}C\{^{1}H\}$ NMR (100 MHz, $CDCl_3$) spectrum of compound **4h**

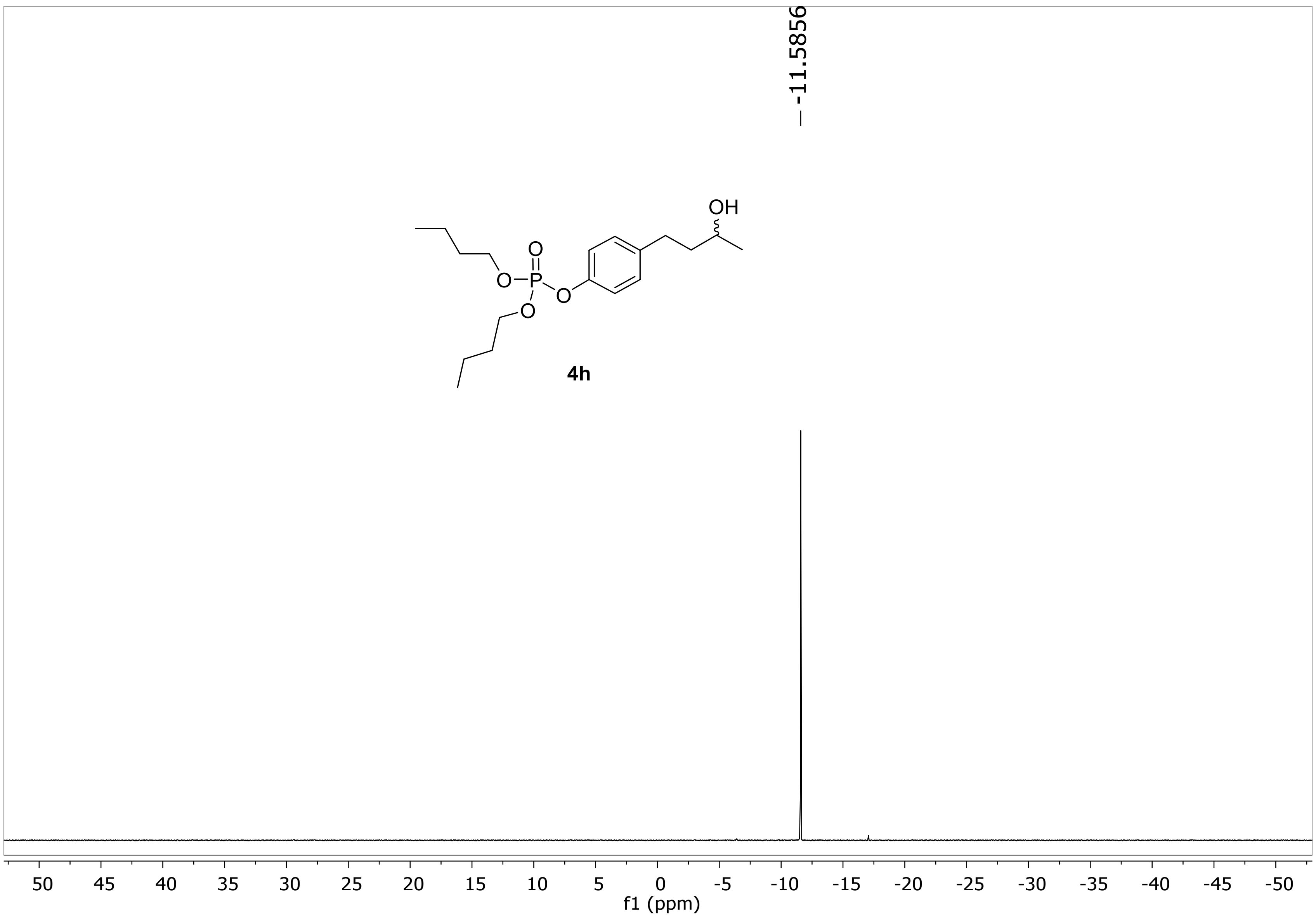

Figure S50. $^{31}P\{^{1}H\}$ NMR (162 MHz, $CDCl_3$) spectrum of compound **4h**

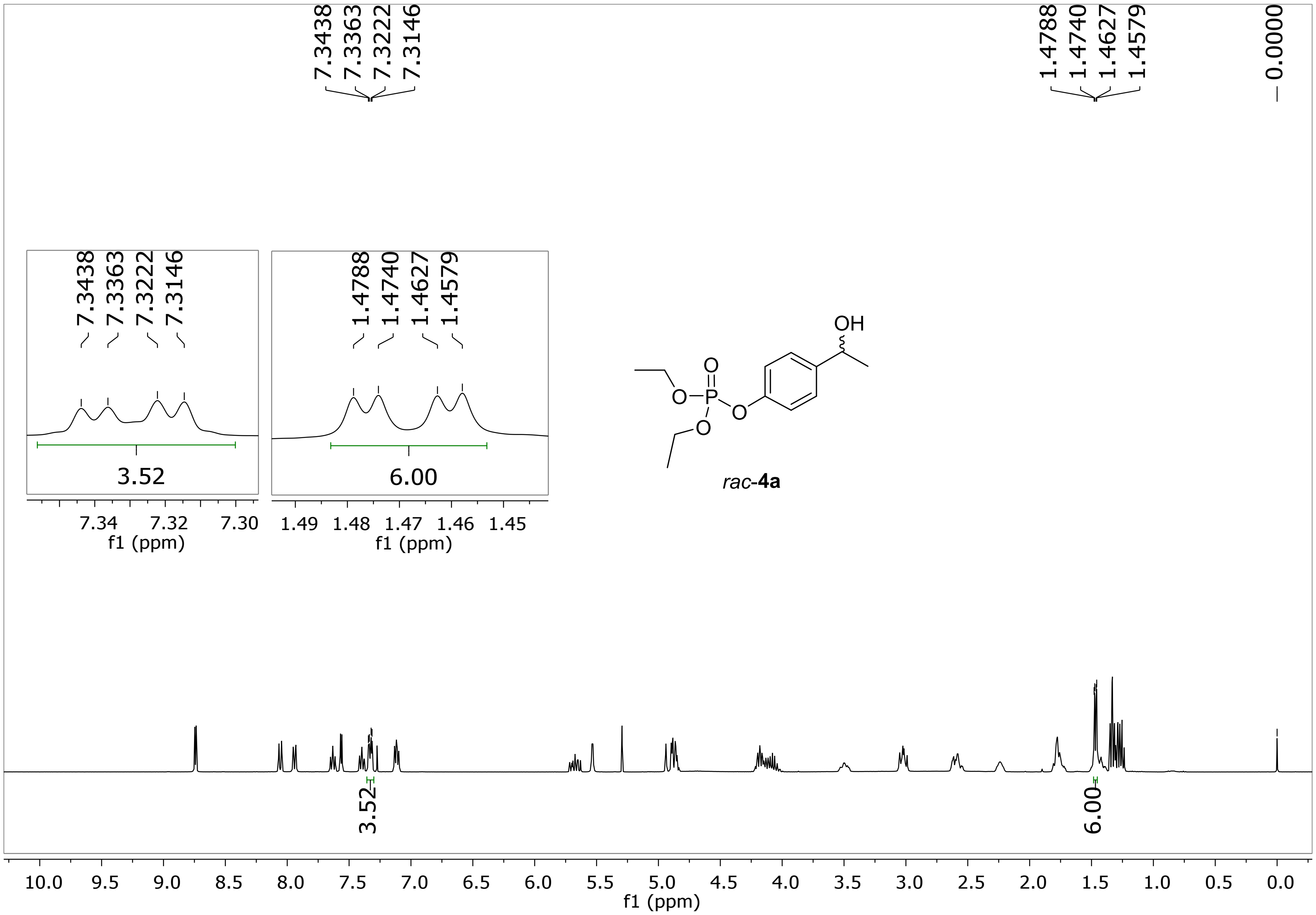

Figure S51. Chiral discrimination spectrum of racemic alcohol **4a** by ^{1}H NMR (400 MHz, $CDCl_3$) in cinchonidine

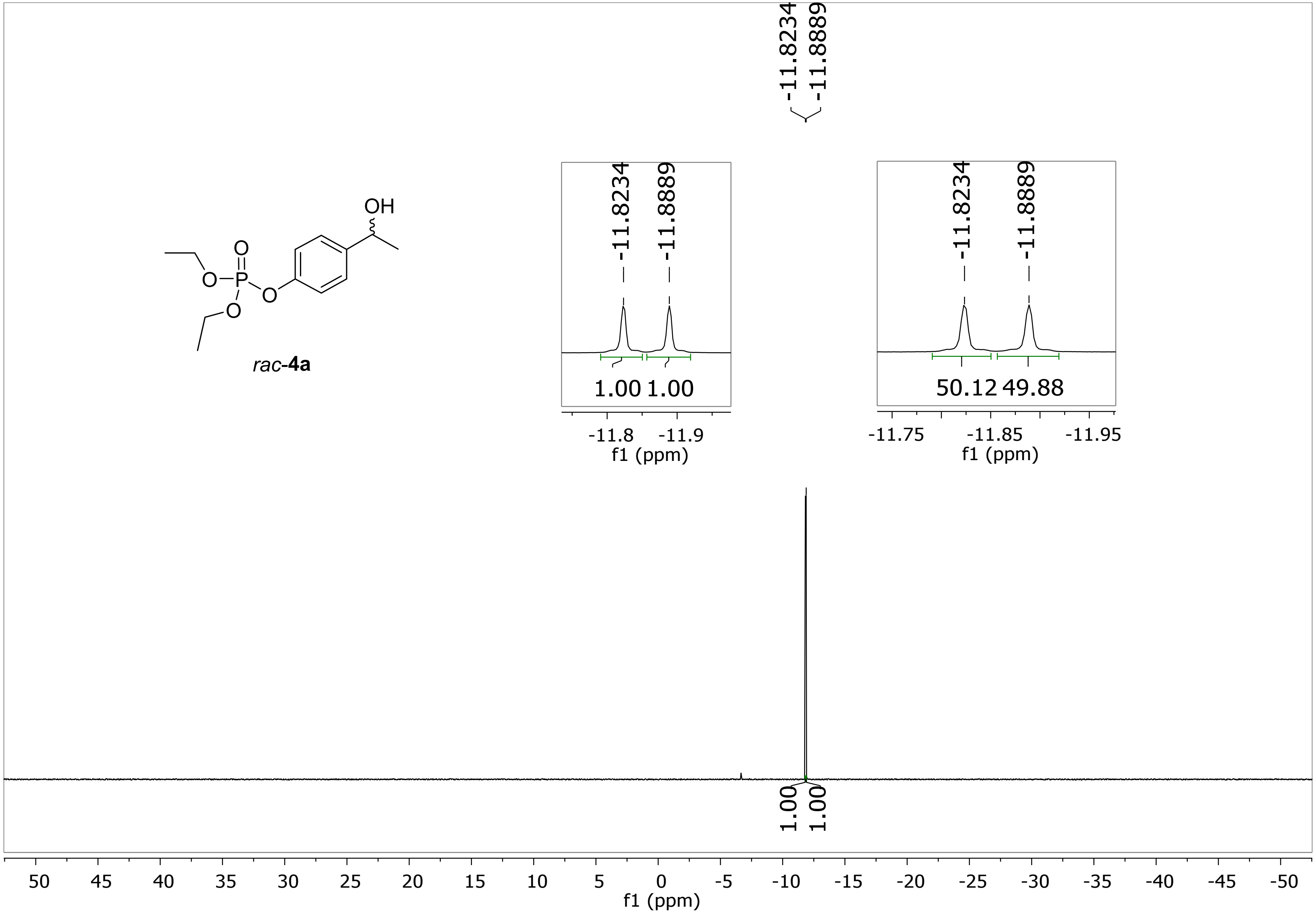

Figure S52. Chiral discrimination spectrum of racemic alcohol **4a** by $^{31}P\{^1H\}$ NMR (162 MHz, $CDCl_3$) in cinchonidine

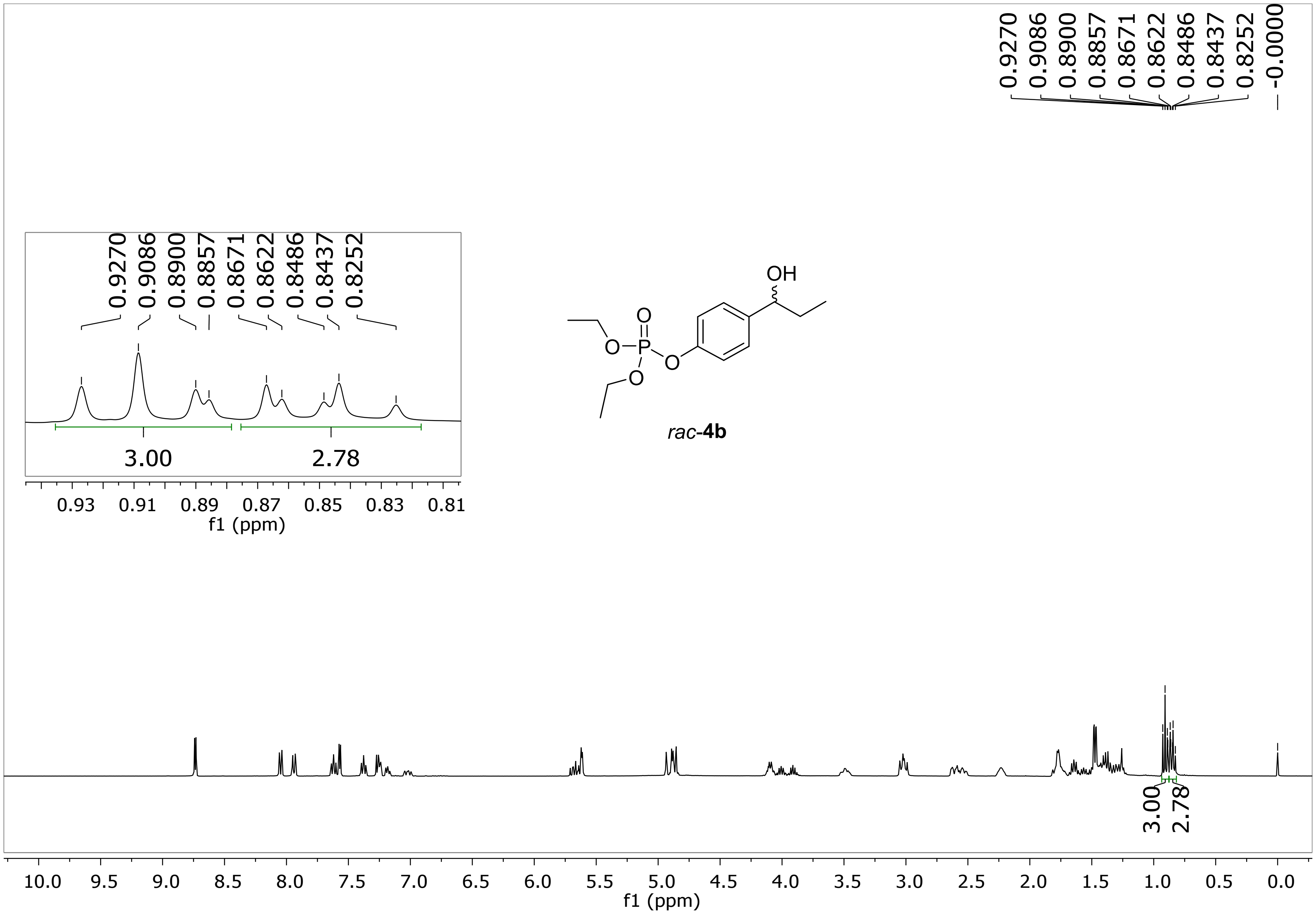

Figure S53. Chiral discrimination spectrum of racemic alcohol **4b** by ^{1}H NMR (400 MHz, $CDCl_3$) in cinchonidine

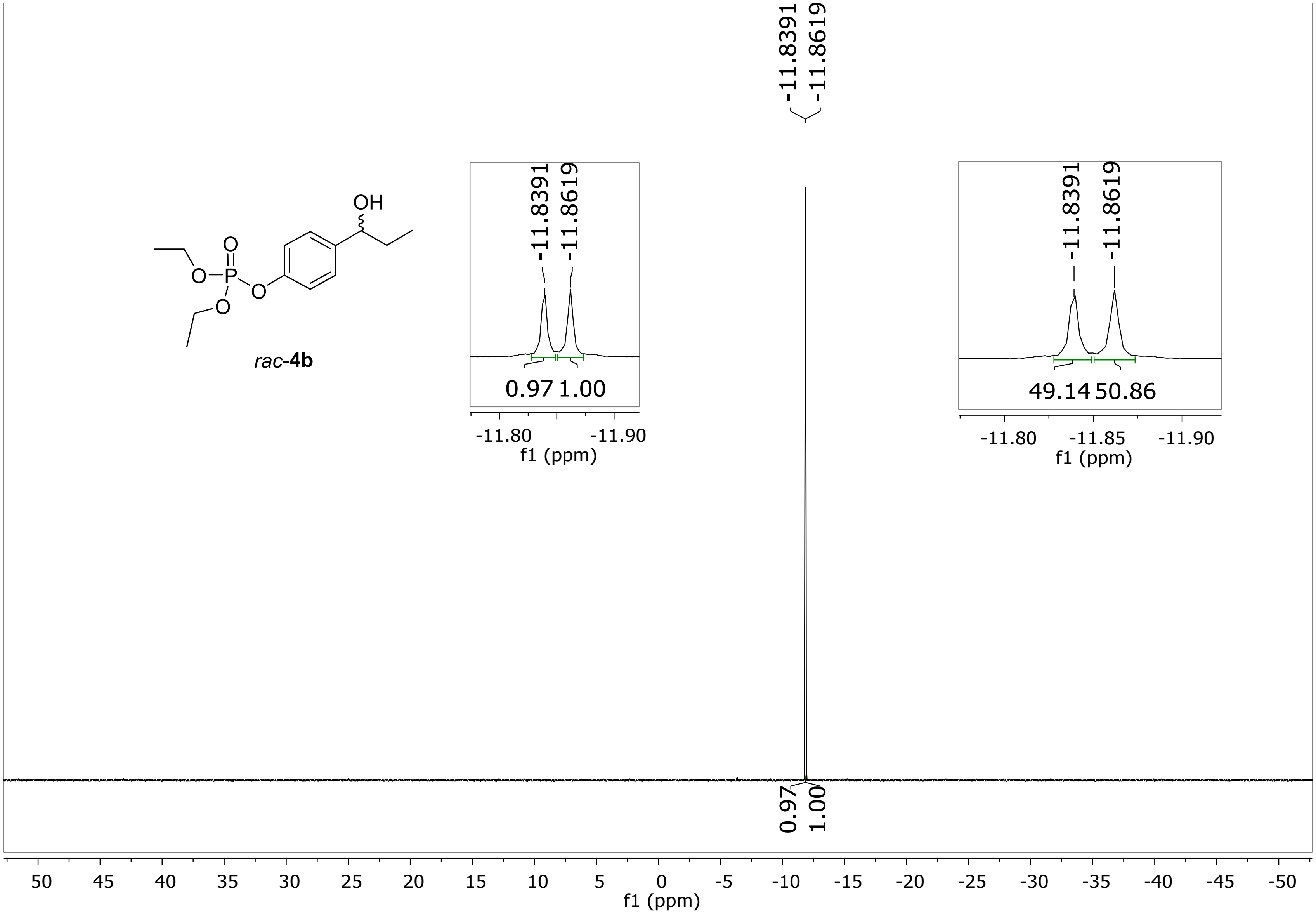

Figure S54. Chiral discrimination spectrum of racemic alcohol **4b** by $^{31}P\{^{1}H\}$ NMR (162 MHz, $CDCl_3$) in cinchonidine

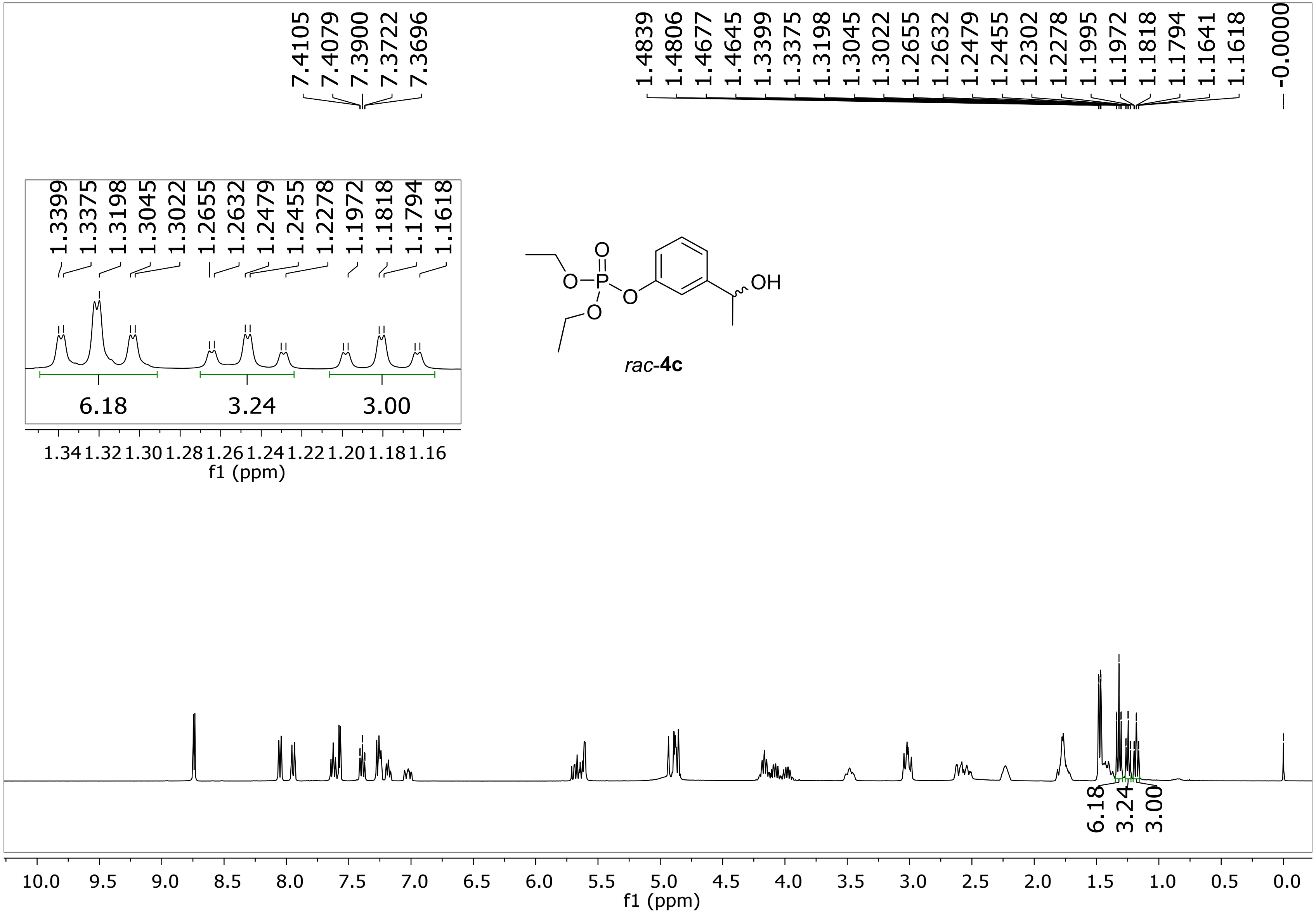

Figure S55. Chiral discrimination spectrum of racemic alcohol **4c** by ^{1}H NMR (400 MHz, $CDCl_3$) in cinchonidine

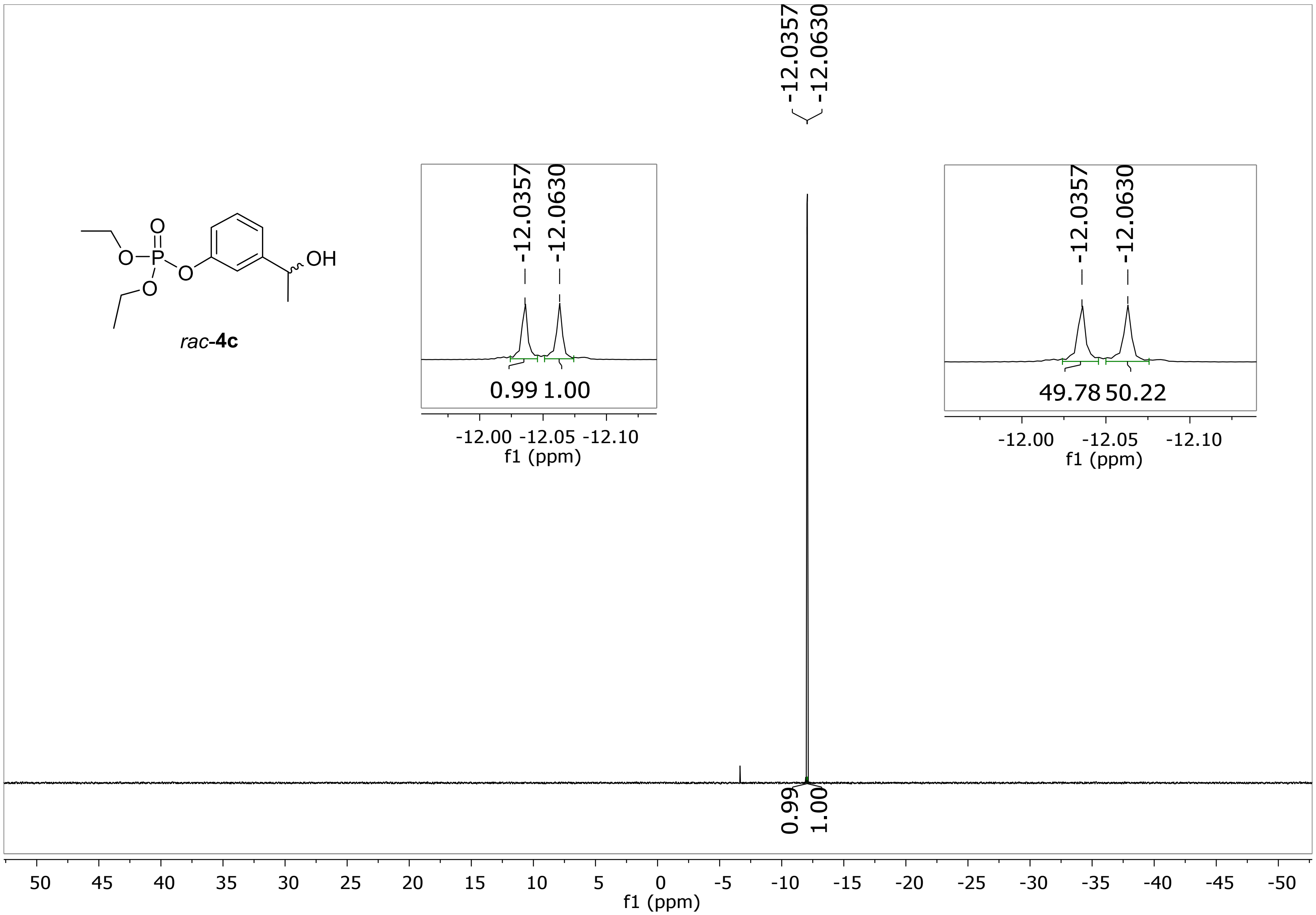

Figure S56. Chiral discrimination spectrum of racemic alcohol **4c** by $^{31}P\{^{1}H\}$ NMR (162 MHz, $CDCl_3$) in cinchonidine

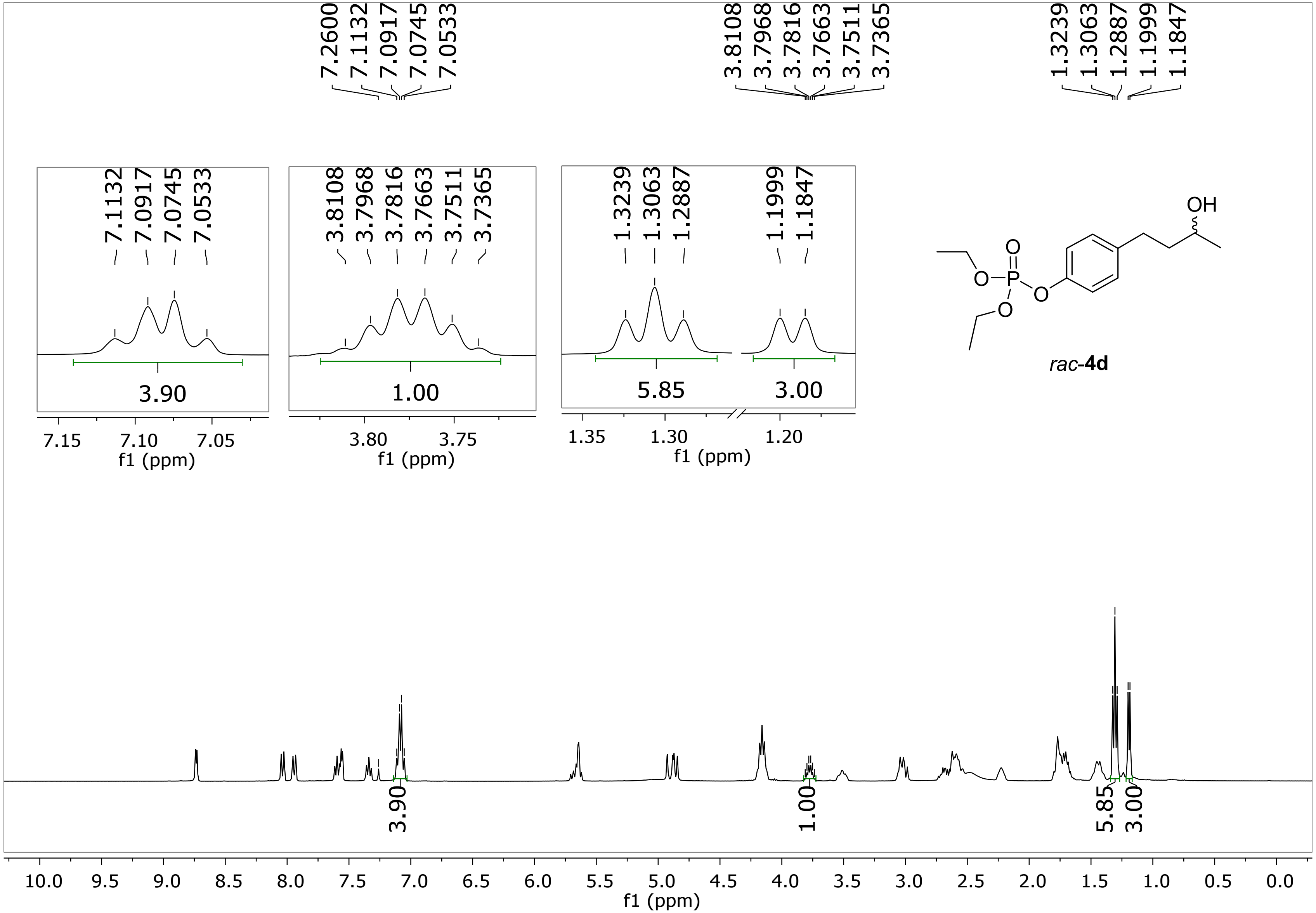

Figure S57. Chiral discrimination spectrum of racemic alcohol **4d** by ^{1}H NMR (400 MHz, $CDCl_3$) in cinchonidine

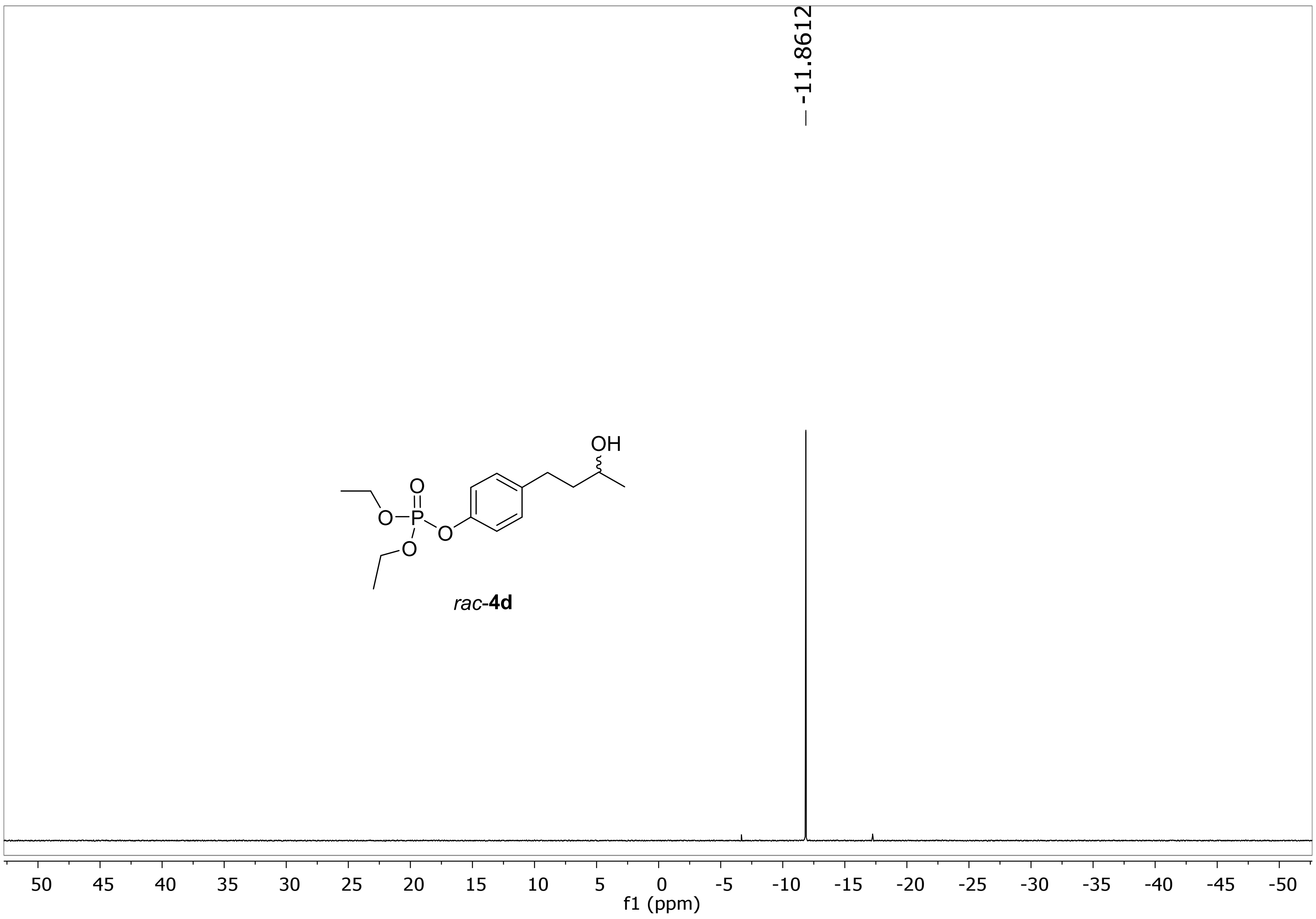

Figure S58. Chiral discrimination spectrum of racemic alcohol **4d** by $^{31}P\{^{1}H\}$ NMR (162 MHz, $CDCl_3$) in cinchonidine

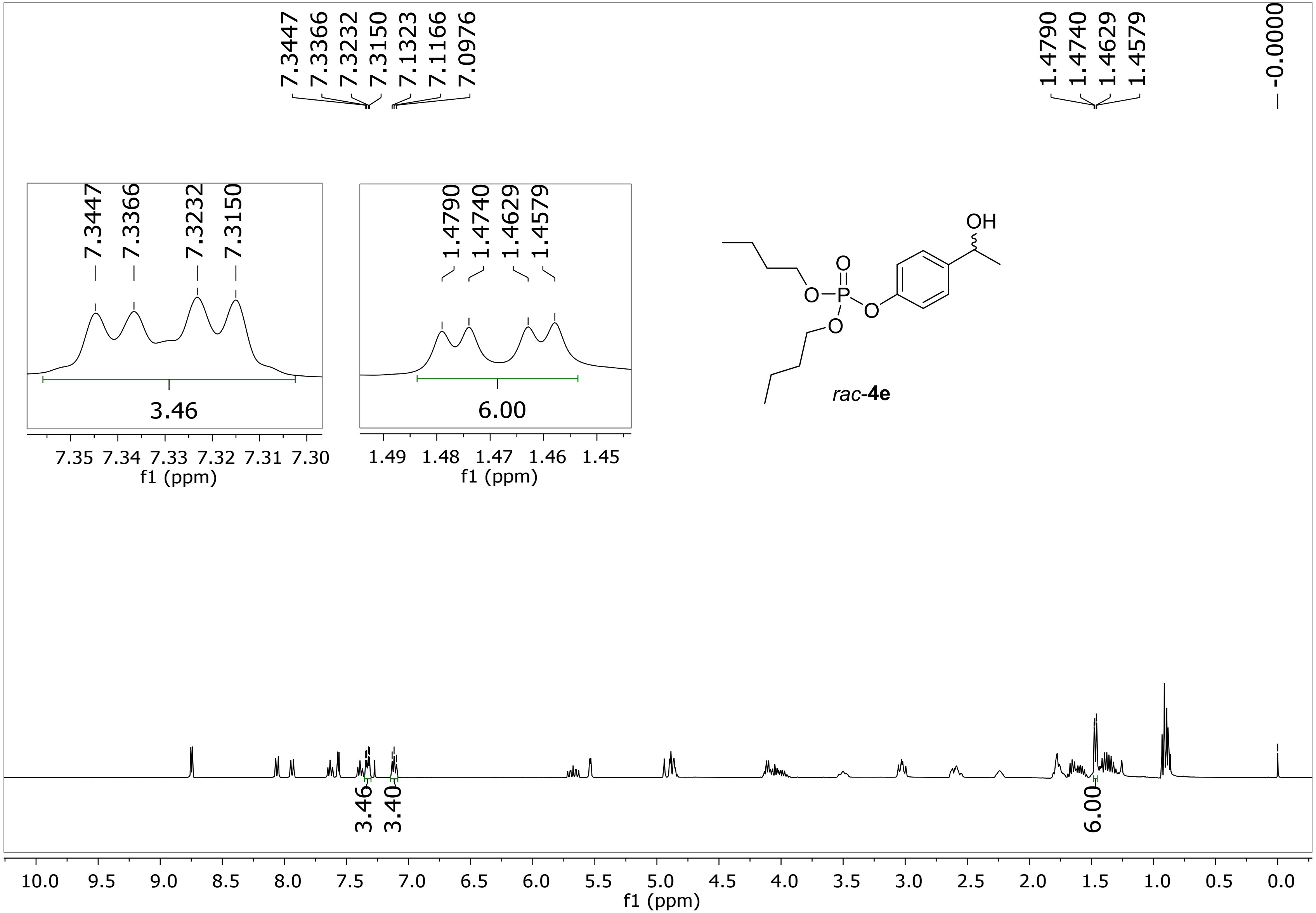

Figure S59. Chiral discrimination spectrum of racemic alcohol **4e** by ^{1}H NMR (400 MHz, $CDCl_3$) in cinchonidine

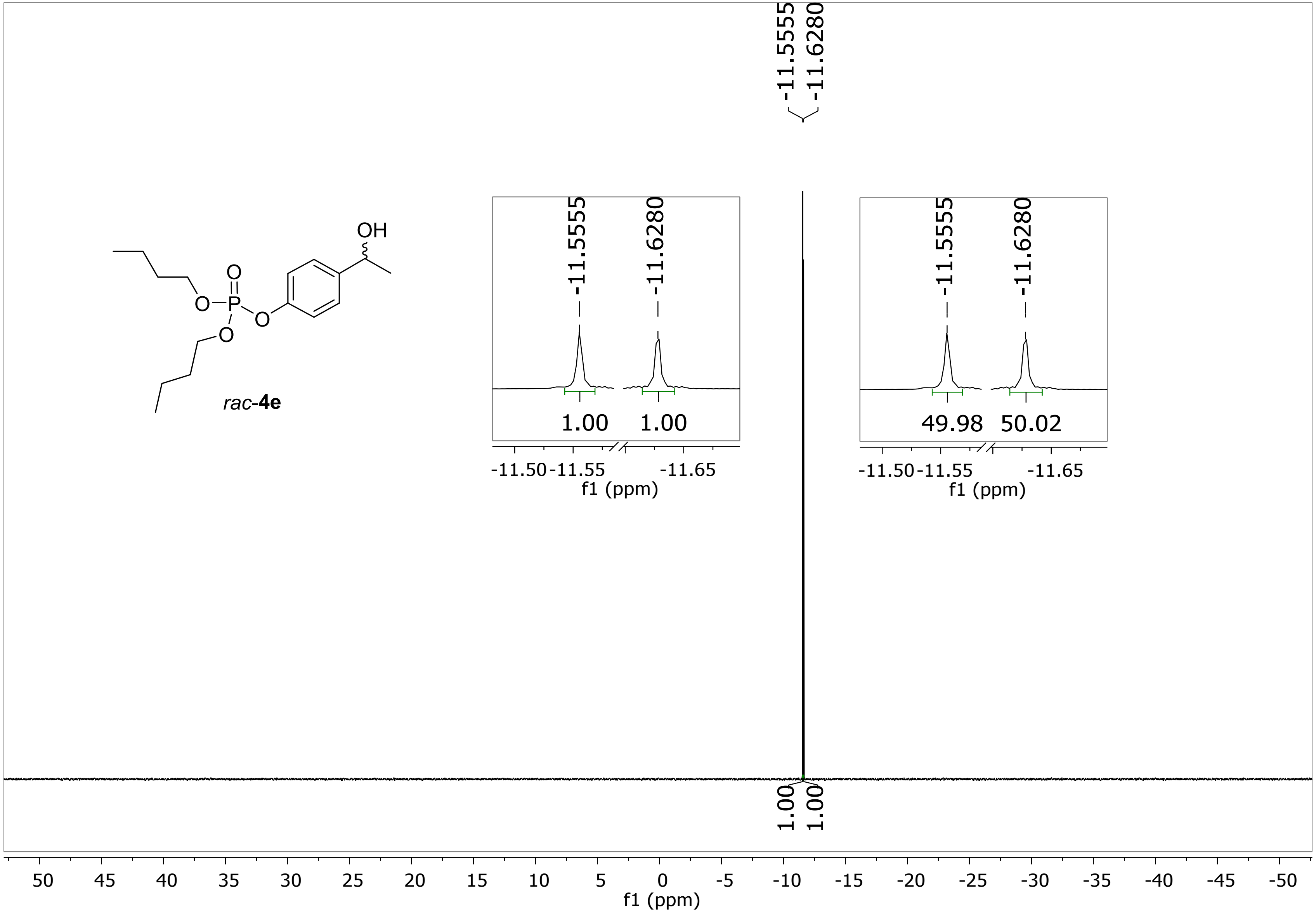

Figure S60. Chiral discrimination spectrum of racemic alcohol **4e** by $^{31}P\{^{1}H\}$ NMR (162 MHz, $CDCl_3$) in cinchonidine

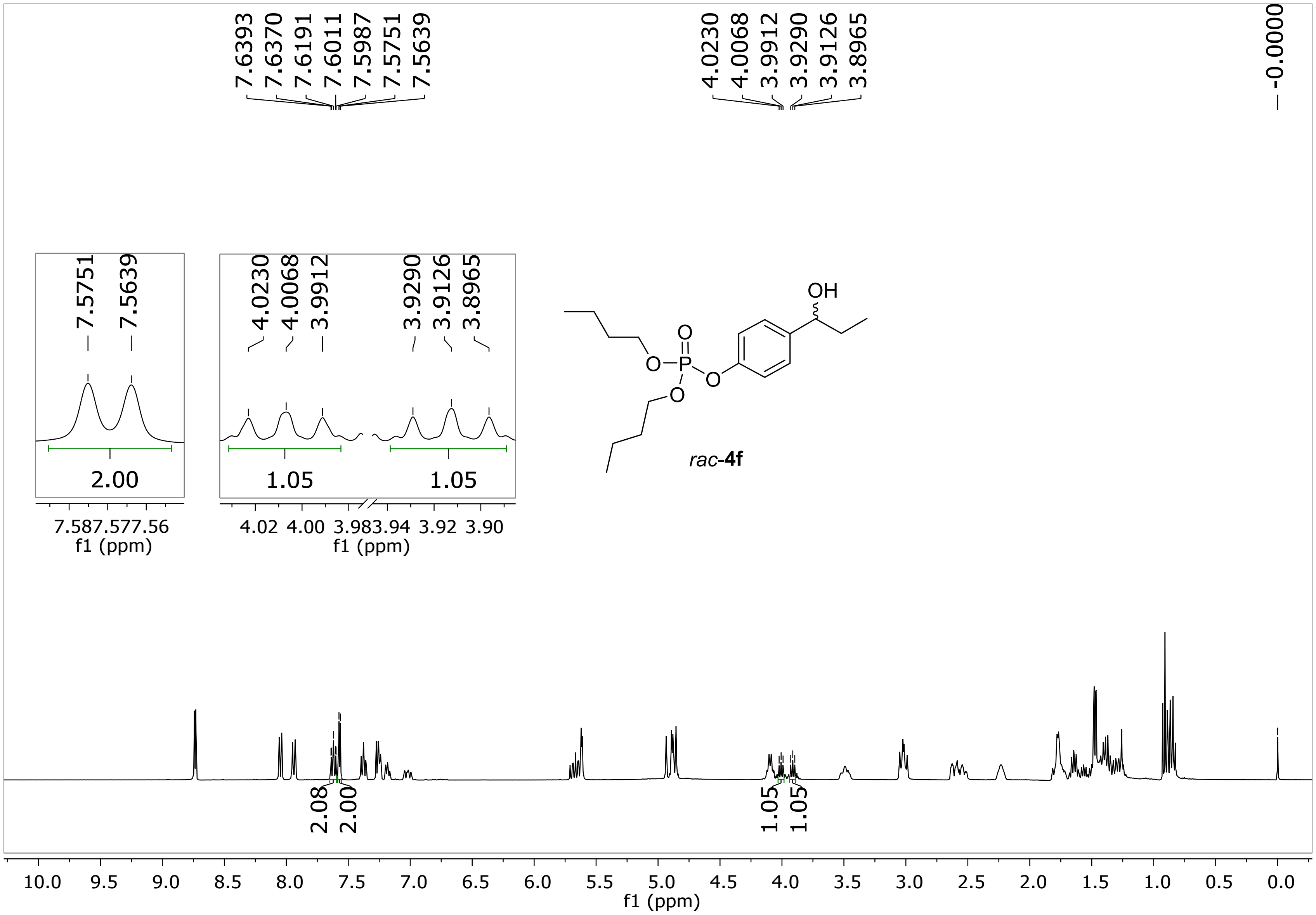

Figure S61. Chiral discrimination spectrum of racemic alcohol **4f** by ^{1}H NMR (400 MHz, $CDCl_3$) in cinchonidine

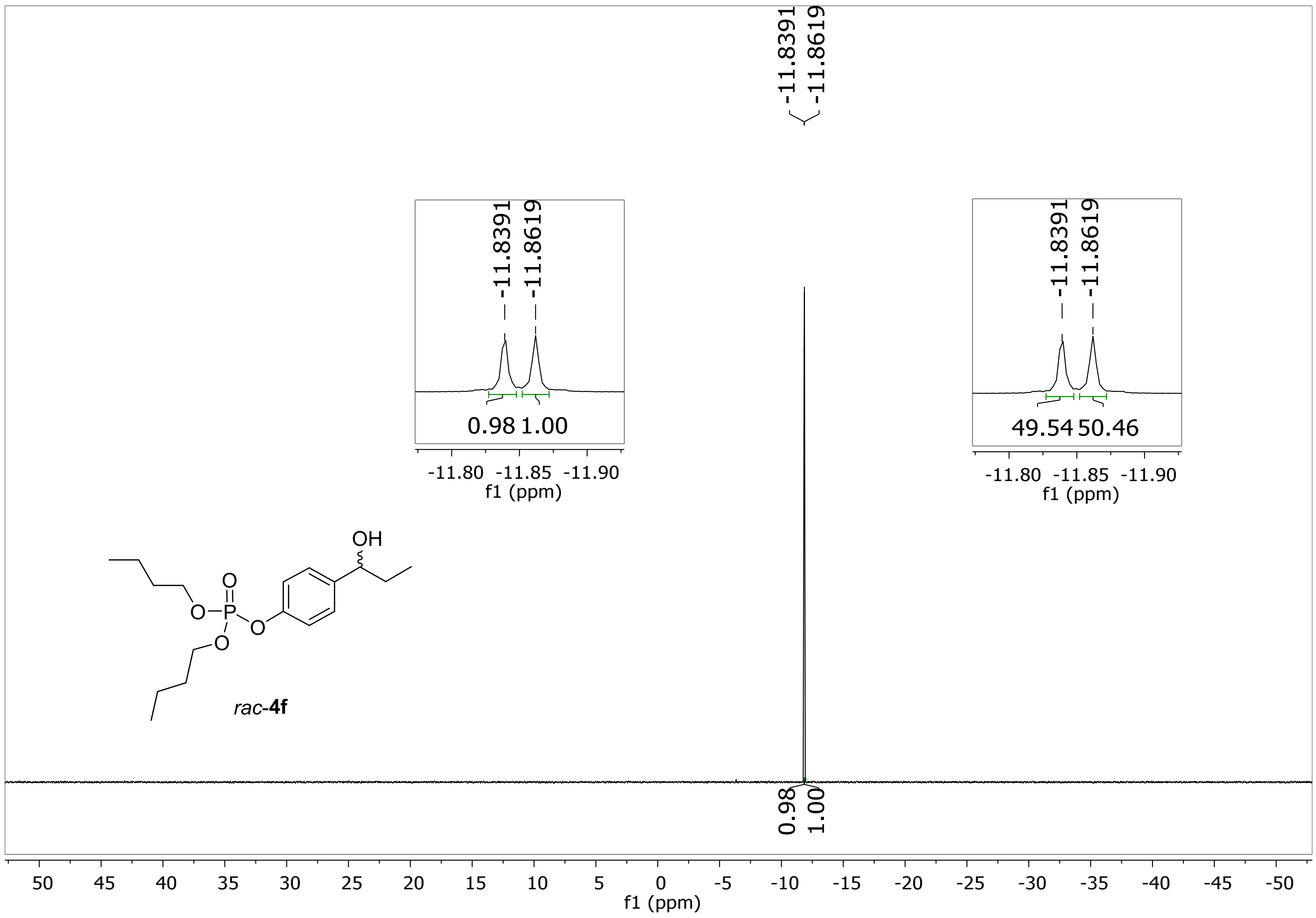

Figure S62. Chiral discrimination spectrum of racemic alcohol **4f** by $^{31}P\{^{1}H\}$ NMR (162 MHz, $CDCl_3$) in cinchonidine

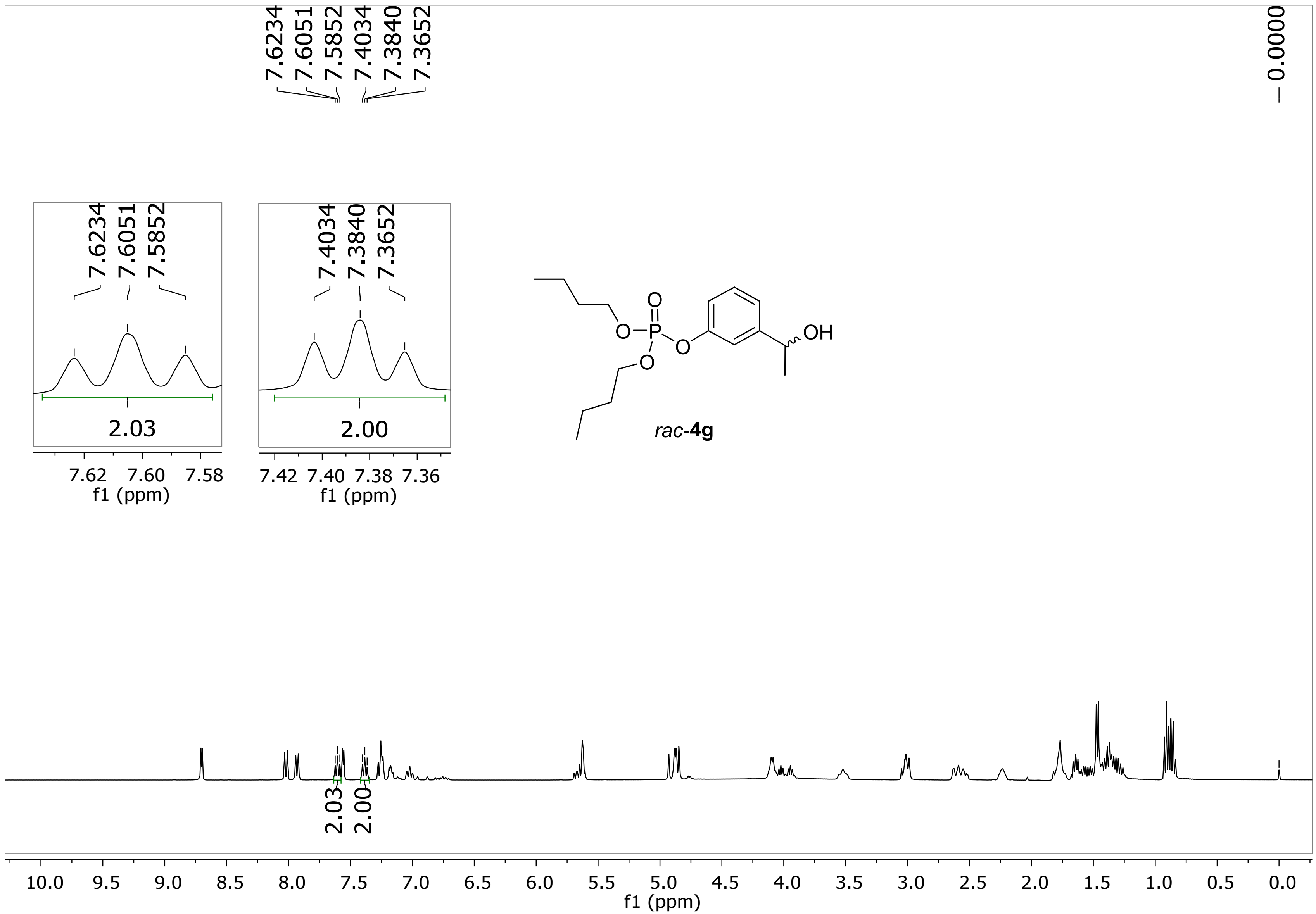

Figure S63. Chiral discrimination spectrum of racemic alcohol **4g** by ^{1}H NMR (400 MHz, $CDCl_3$) in cinchonidine

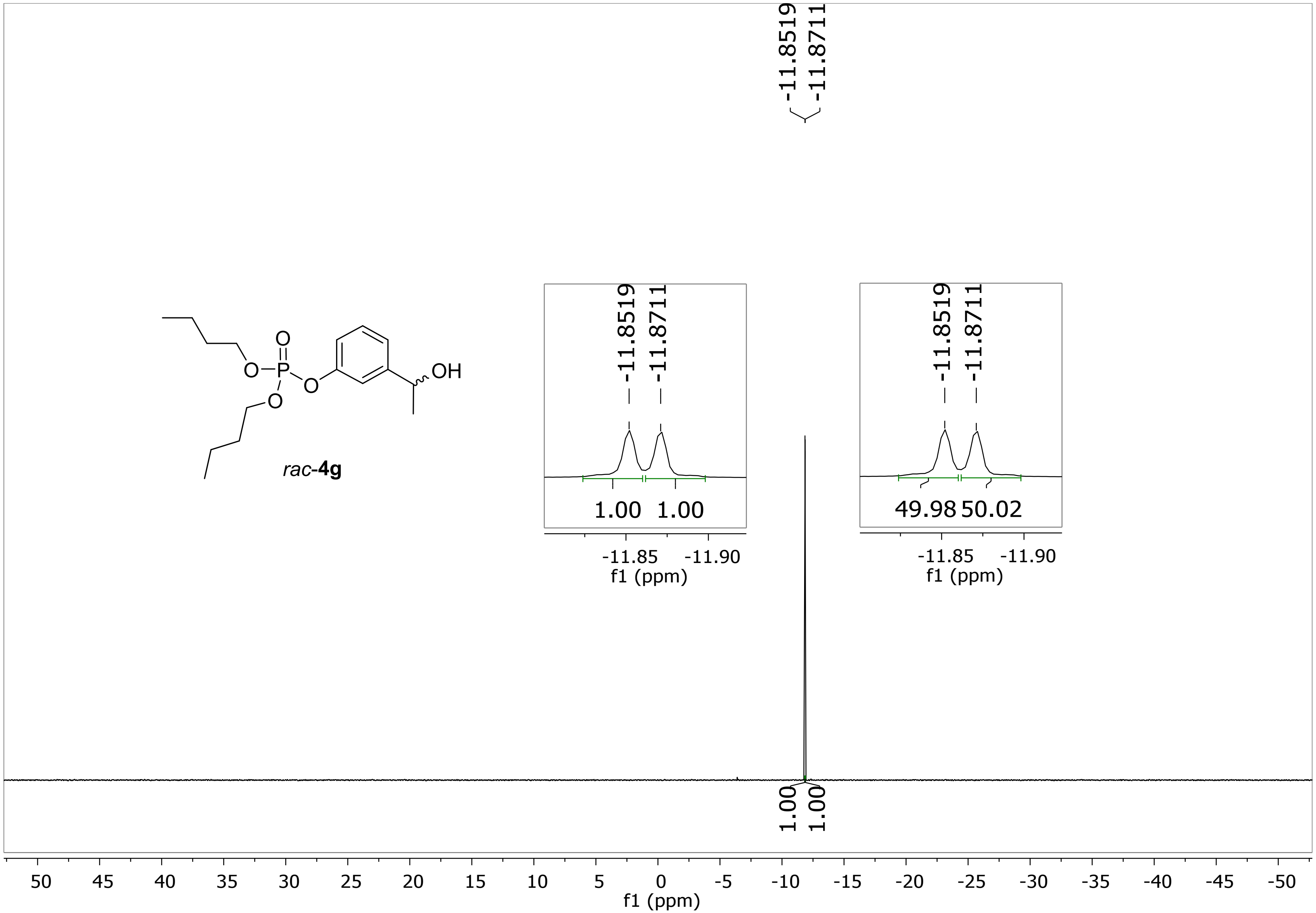

Figure S64. Chiral discrimination spectrum of racemic alcohol **4g** by $^{31}P\{^1H\}$ NMR (162 MHz, $CDCl_3$) in cinchonidine

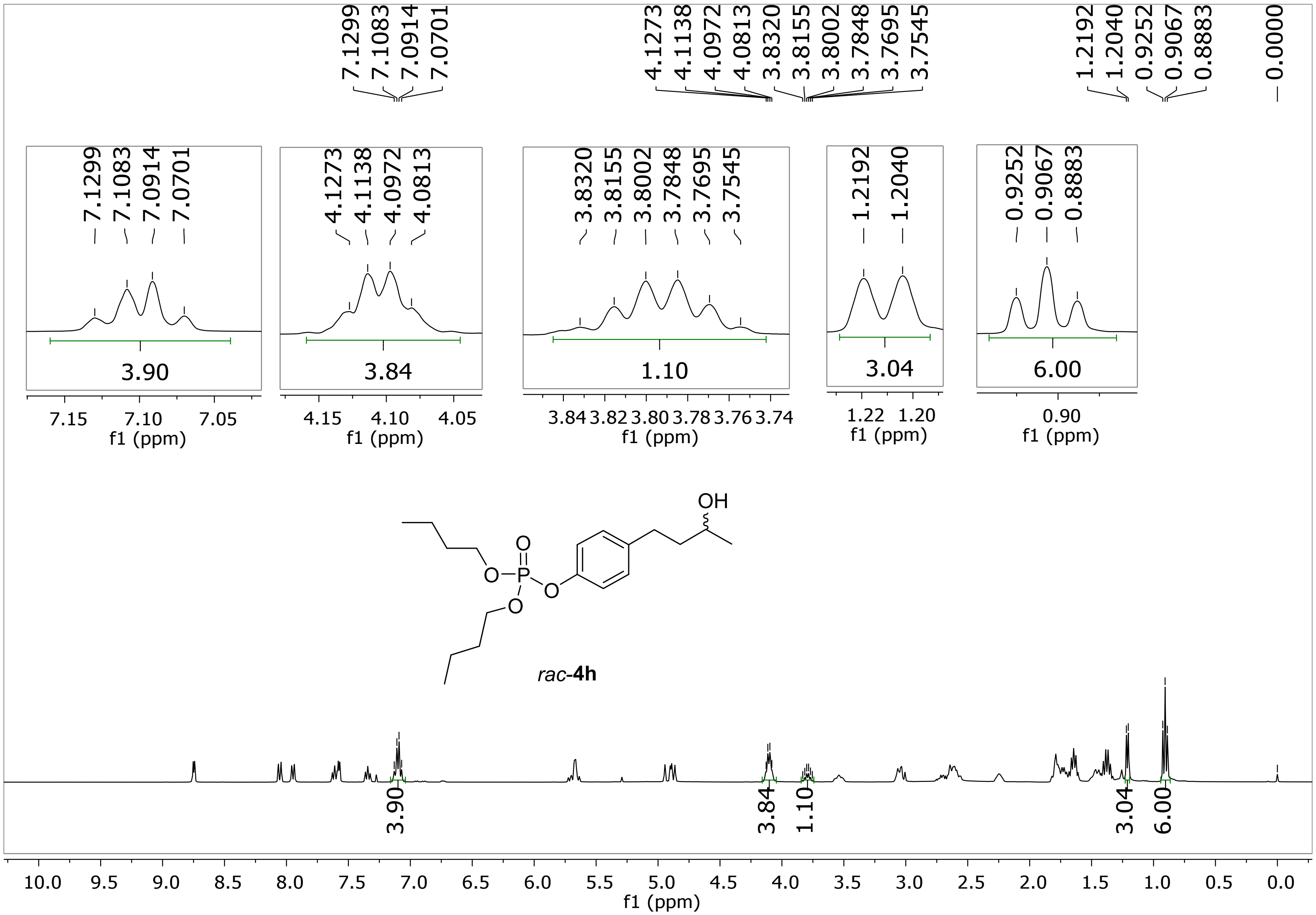

Figure S65. Chiral discrimination spectrum of racemic alcohol **4h** by ^{1}H NMR (400 MHz, $CDCl_3$) in cinchonidine

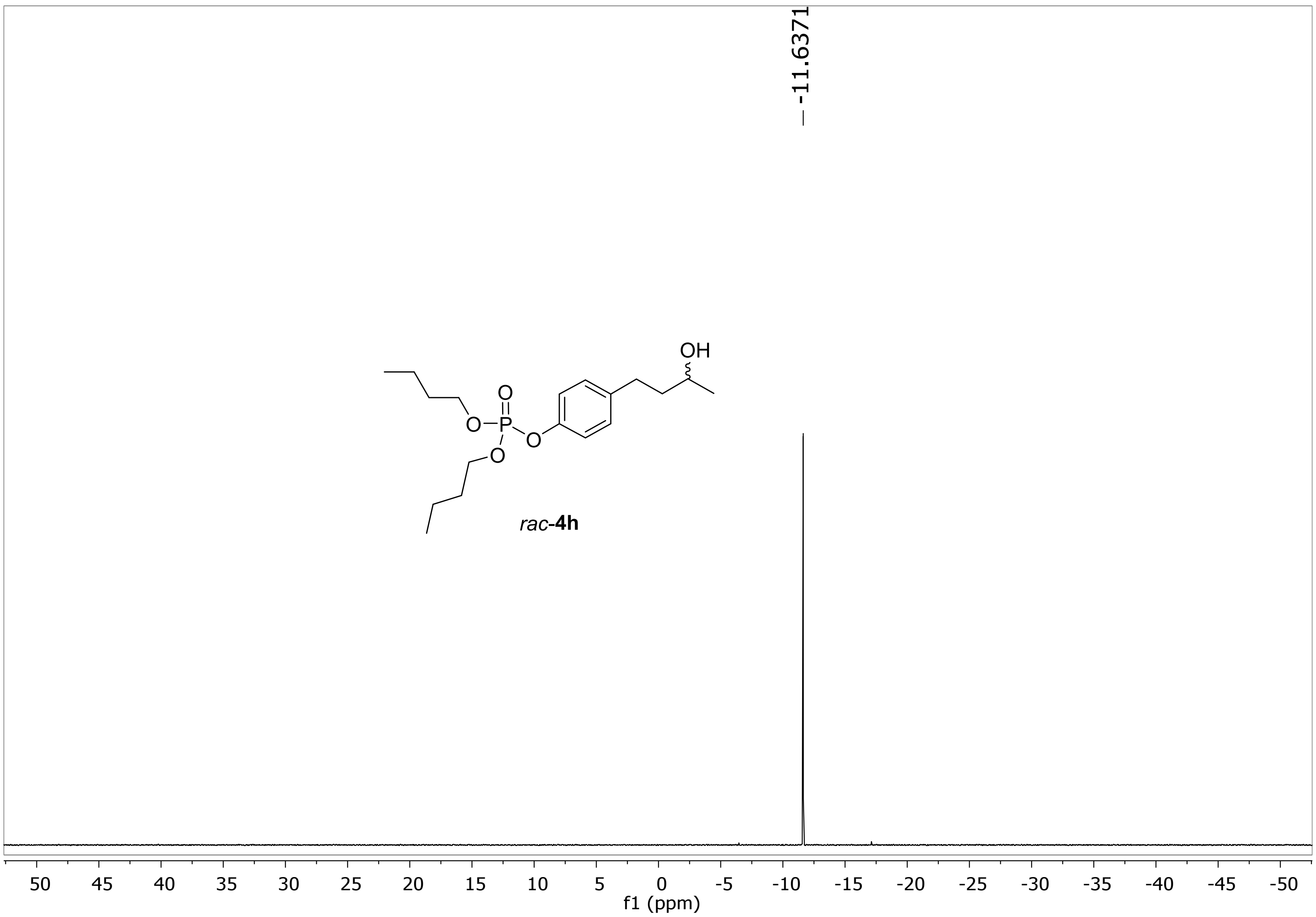

Figure S66. Chiral discrimination spectrum of racemic alcohol **4h** by $^{31}P\{^{1}H\}$ NMR (162 MHz, $CDCl_3$) in cinchonidine

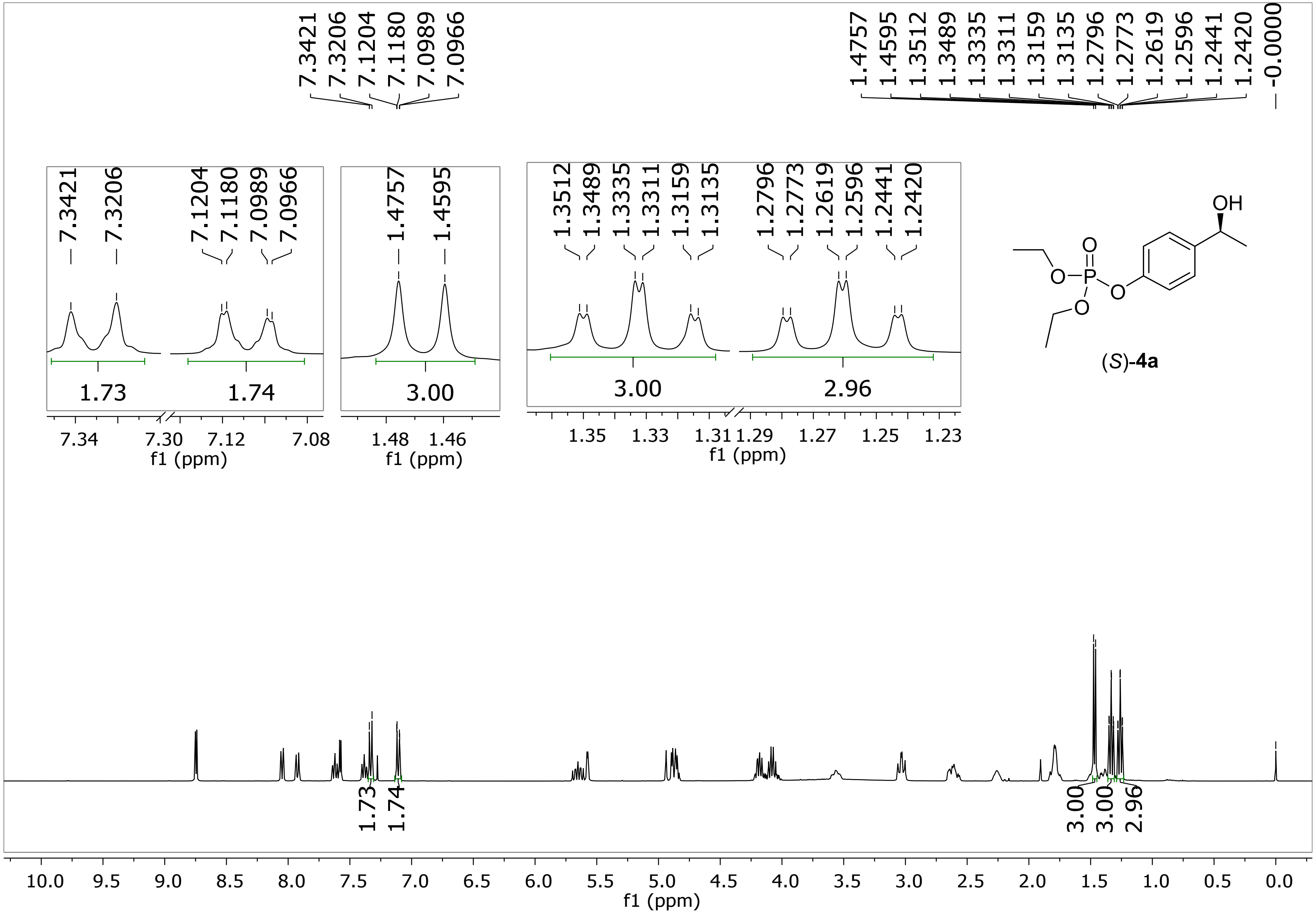

Figure S67. Chiral discrimination of the enantiomerically enriched alcohol **4a** by ^{1}H NMR (400 MHz, $CDCl_3$) in cinchonidine

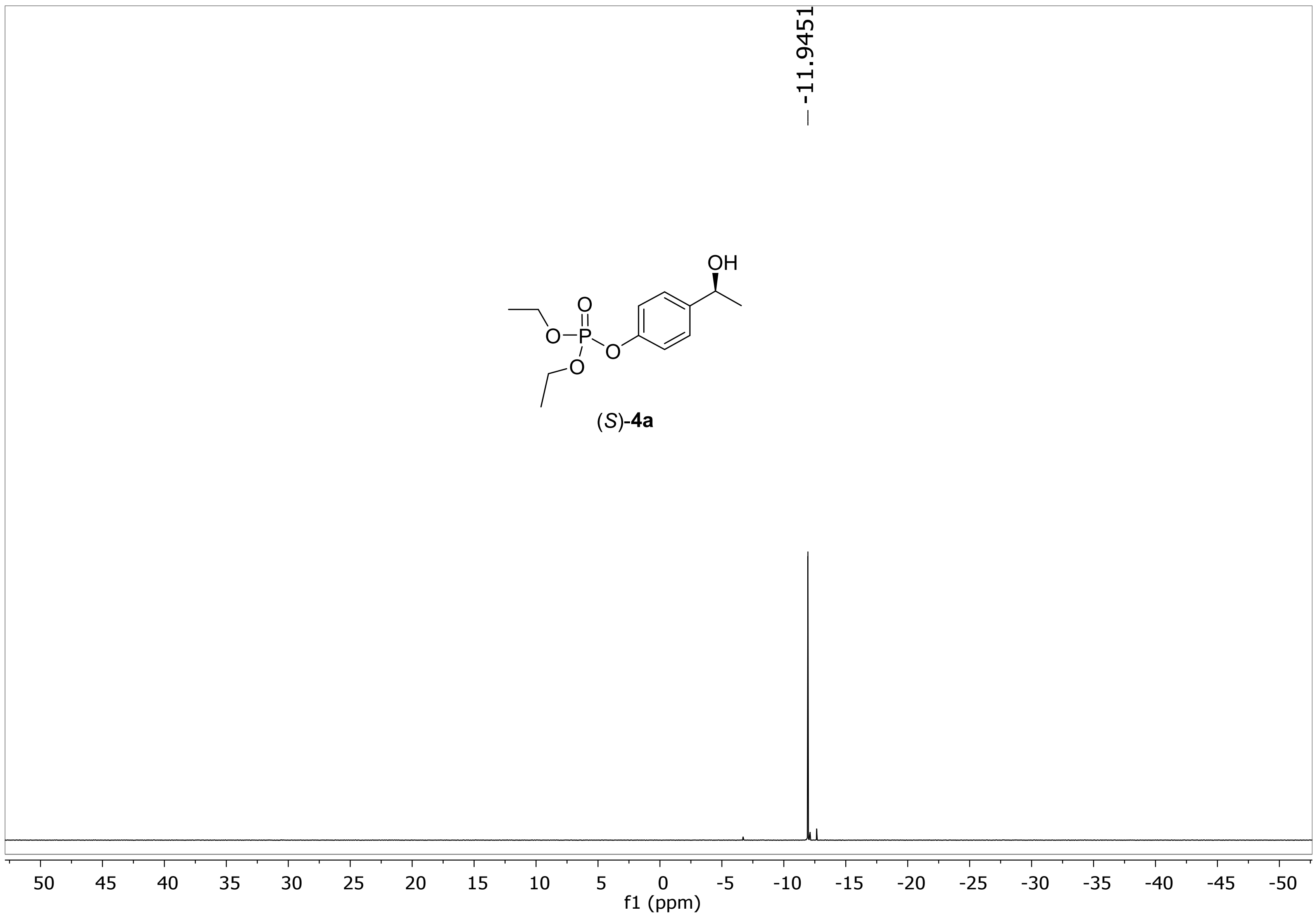

Figure S68. Chiral discrimination of the enantiomerically enriched alcohol **4a** by $^{31}P\{^{1}H\}$ NMR (162 MHz, $CDCl_3$) in cinchonidine

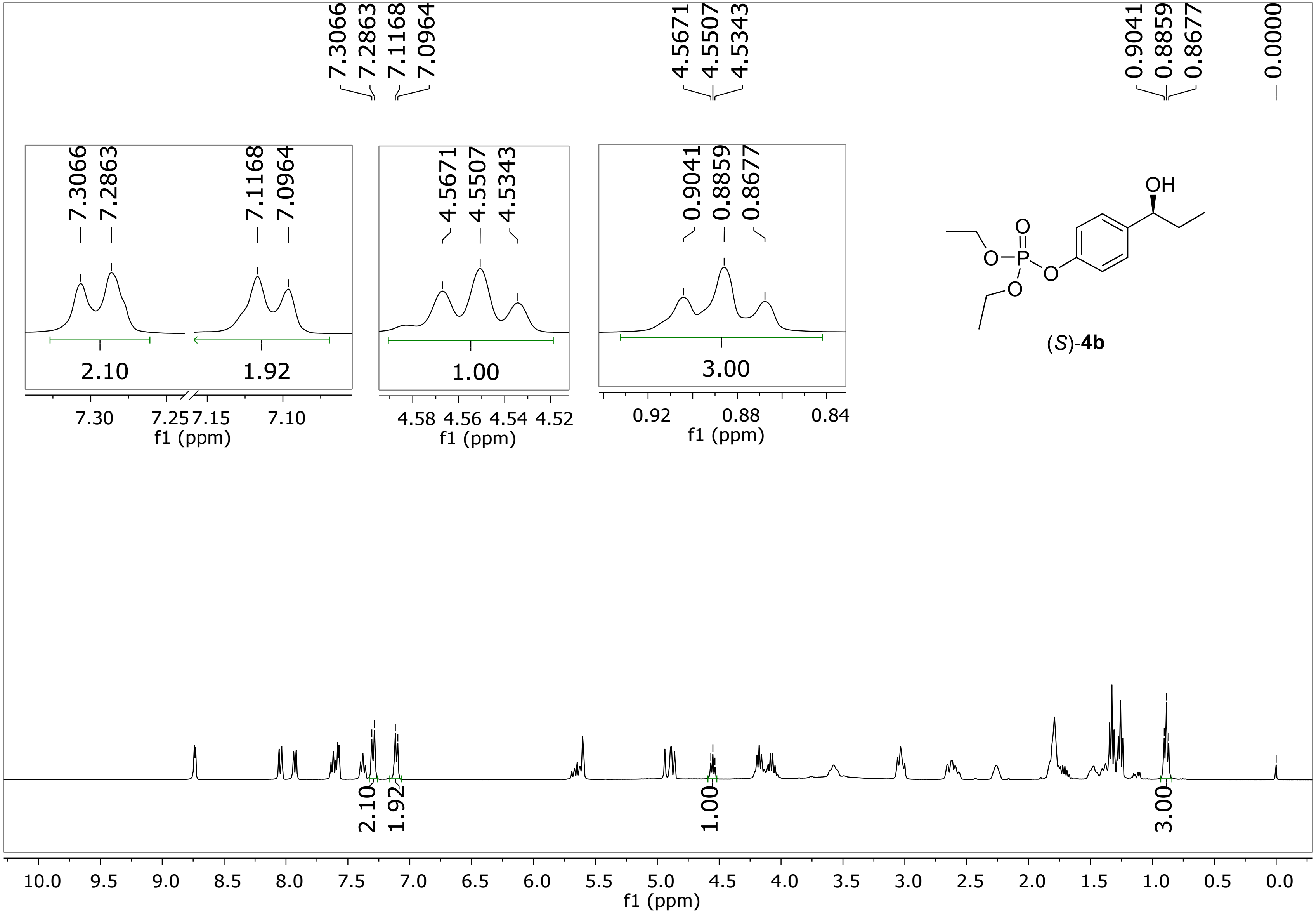

(*S*)-**4b**

Figure S69. Chiral discrimination of the enantiomerically enriched alcohol **4b** by ^{1}H NMR (400 MHz, $CDCl_3$) in cinchonidine

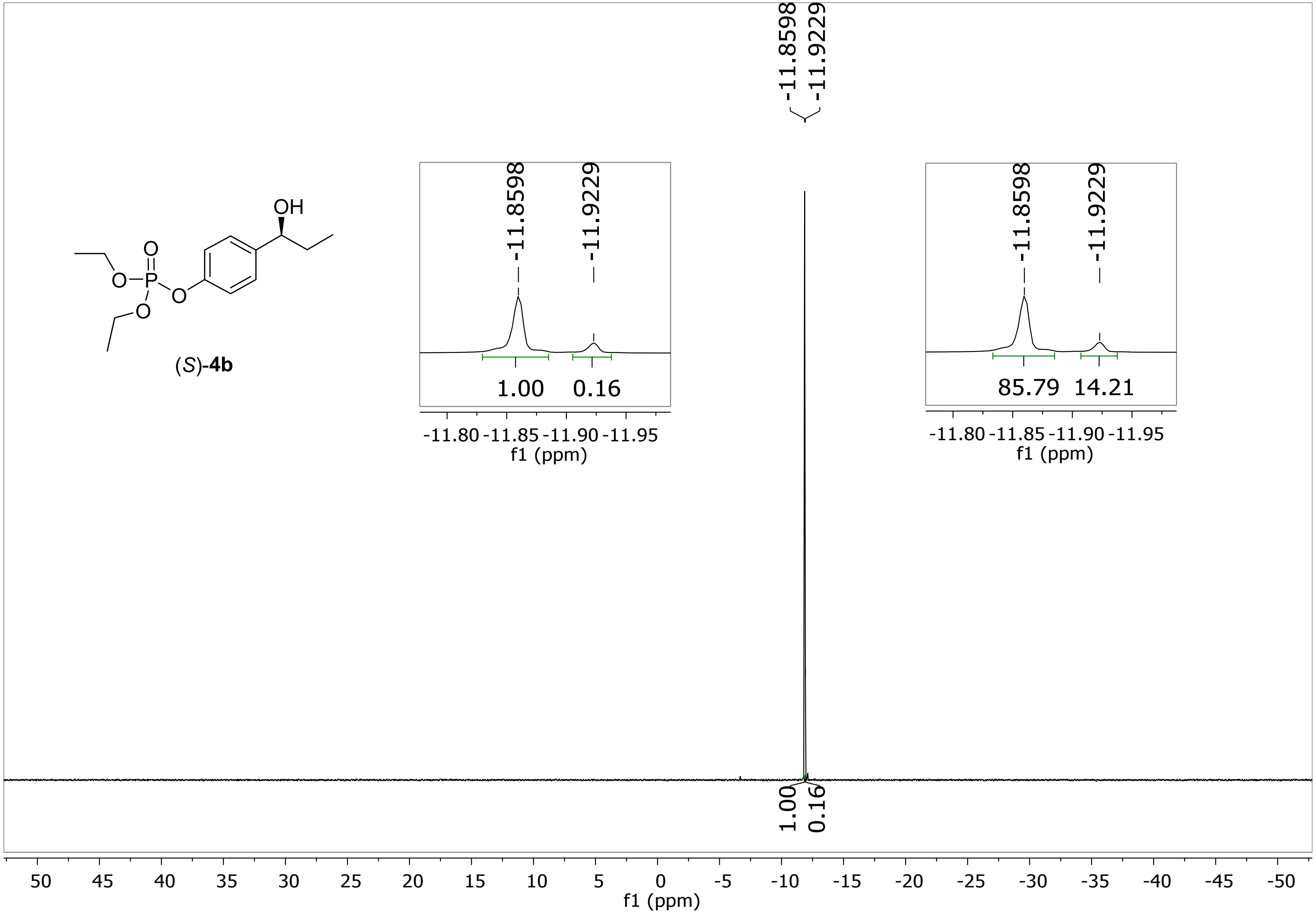

Figure S70. Chiral discrimination of the enantiomerically enriched alcohol **4b** by $^{31}P\{^{1}H\}$ NMR (162 MHz, $CDCl_3$) in cinchonidine

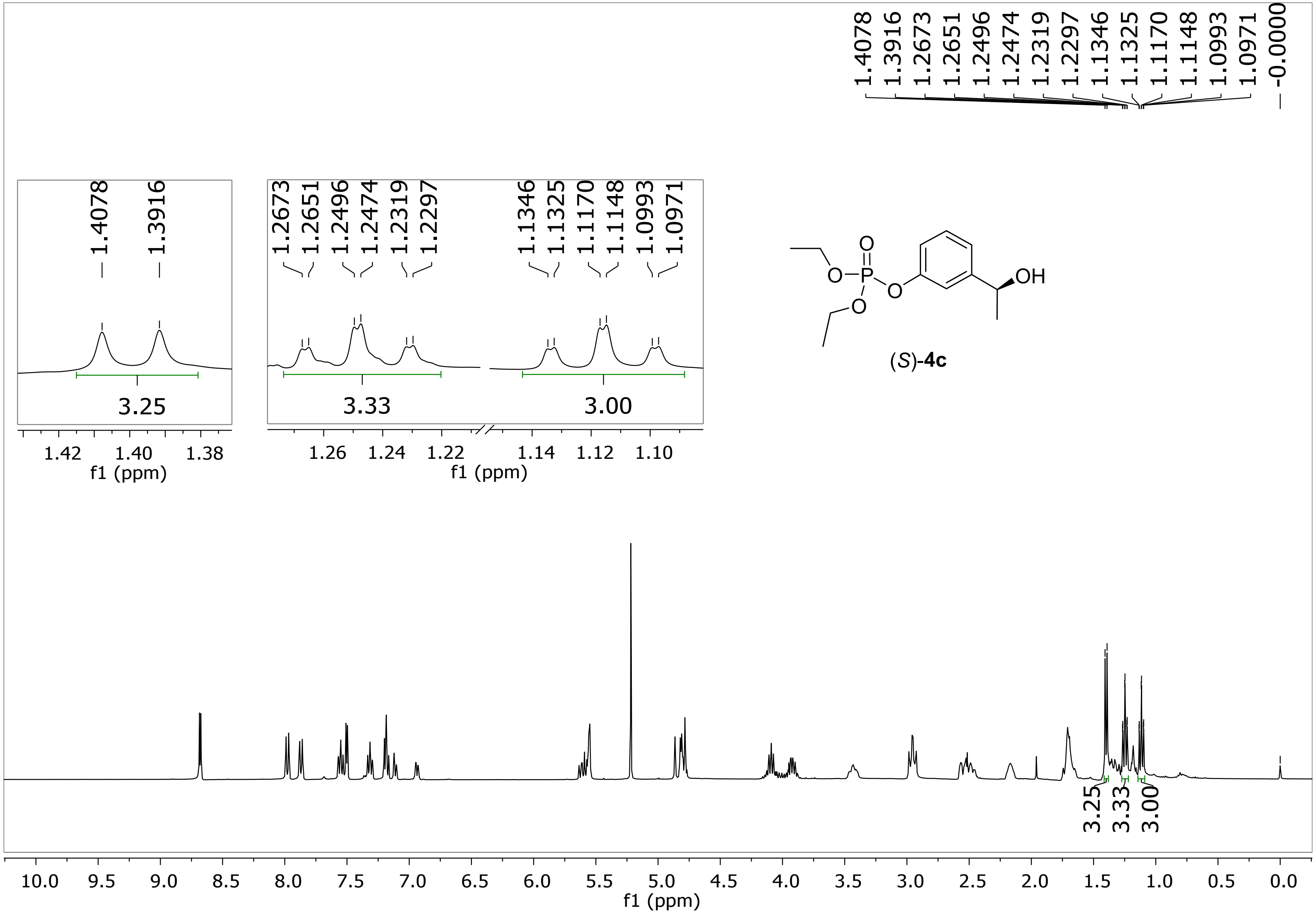

Figure S71. Chiral discrimination of the enantiomerically enriched alcohol **4c** by ^{1}H NMR (400 MHz, $CDCl_3$) in cinchonidine

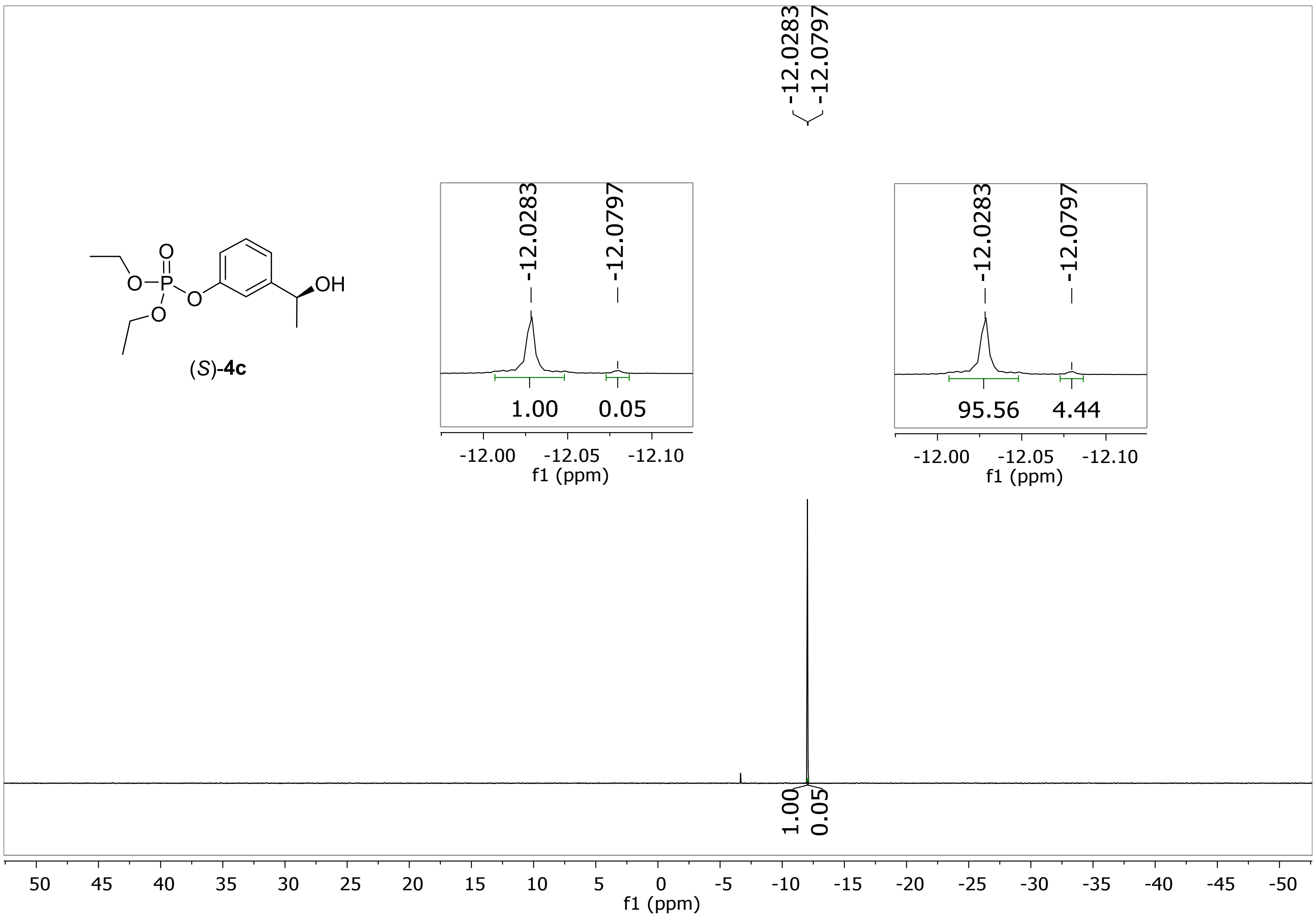

Figure S72. Chiral discrimination of the enantiomerically enriched alcohol **4c** by $^{31}P\{^{1}H\}$ NMR (162 MHz, $CDCl_3$) in cinchonidine

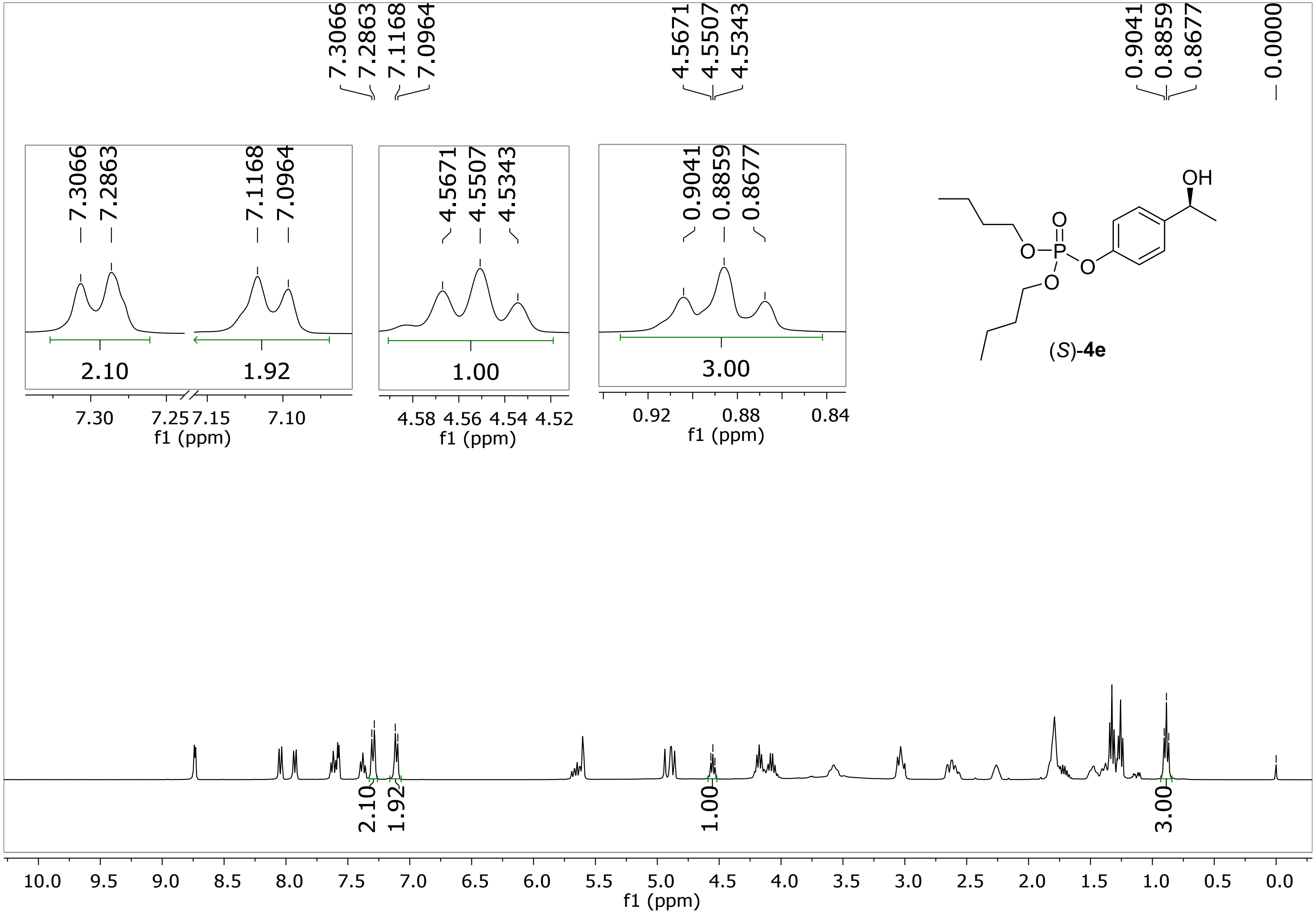

Figure S73. Chiral discrimination of the enantiomerically enriched alcohol **4e** by ^{1}H NMR (400 MHz, $CDCl_3$) in cinchonidine

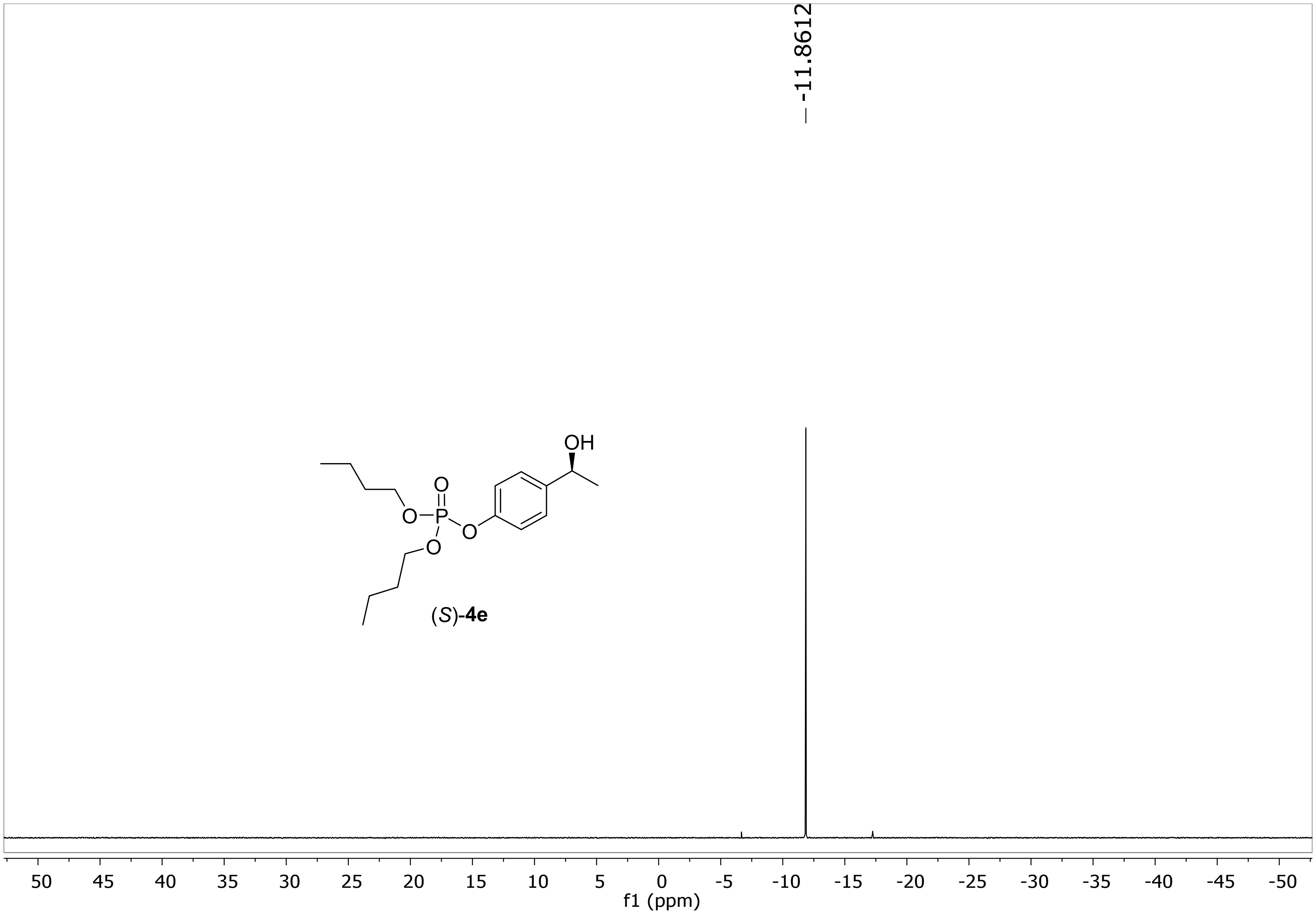

Figure S74. Chiral discrimination of the enantiomerically enriched alcohol **4e** by $^{31}P\{^{1}H\}$ NMR (162 MHz, $CDCl_3$) in cinchonidine

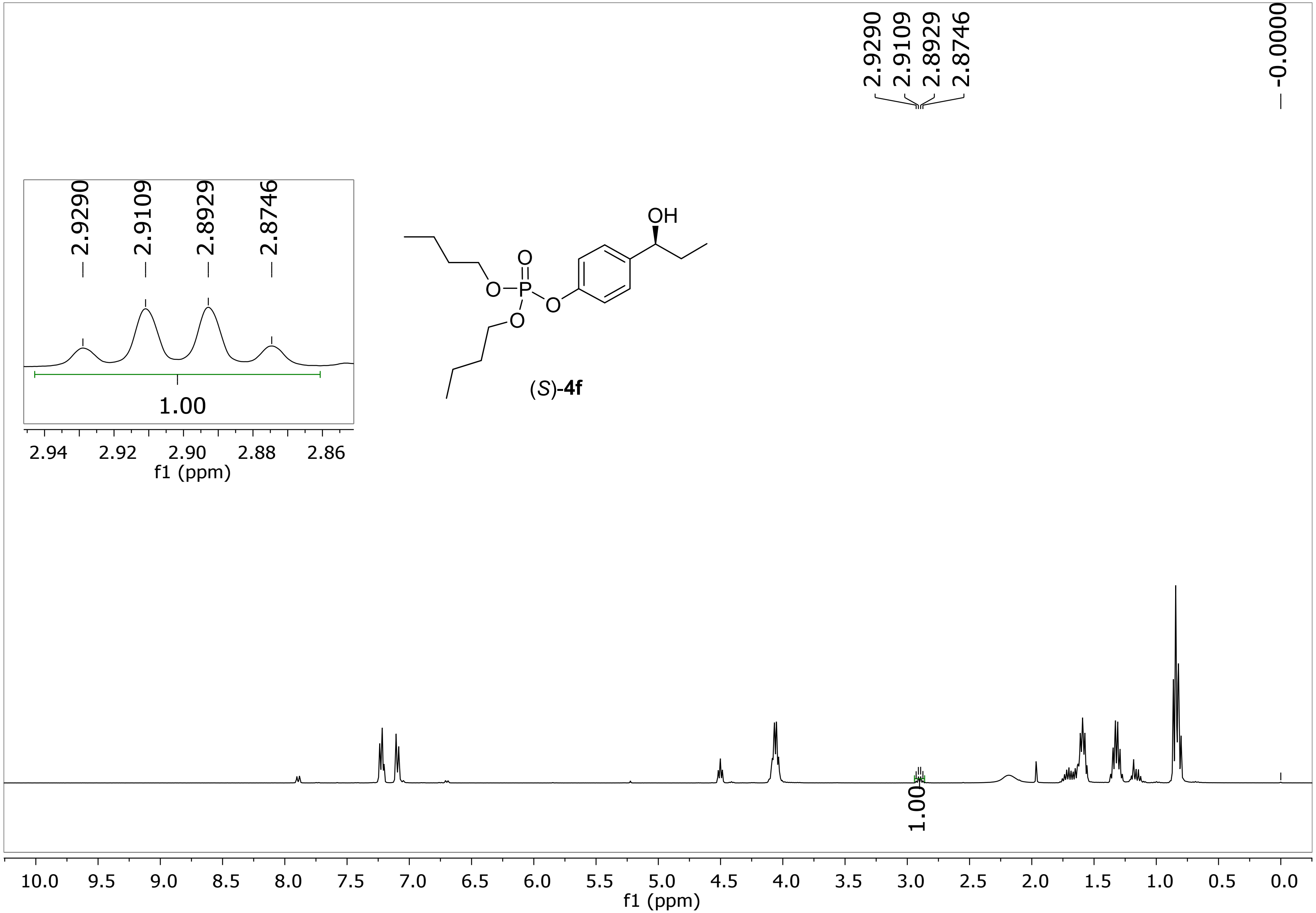

Figure S75. Chiral discrimination of the enantiomerically enriched alcohol **4f** by ^{1}H NMR (400 MHz, $CDCl_3$) in cinchonidine

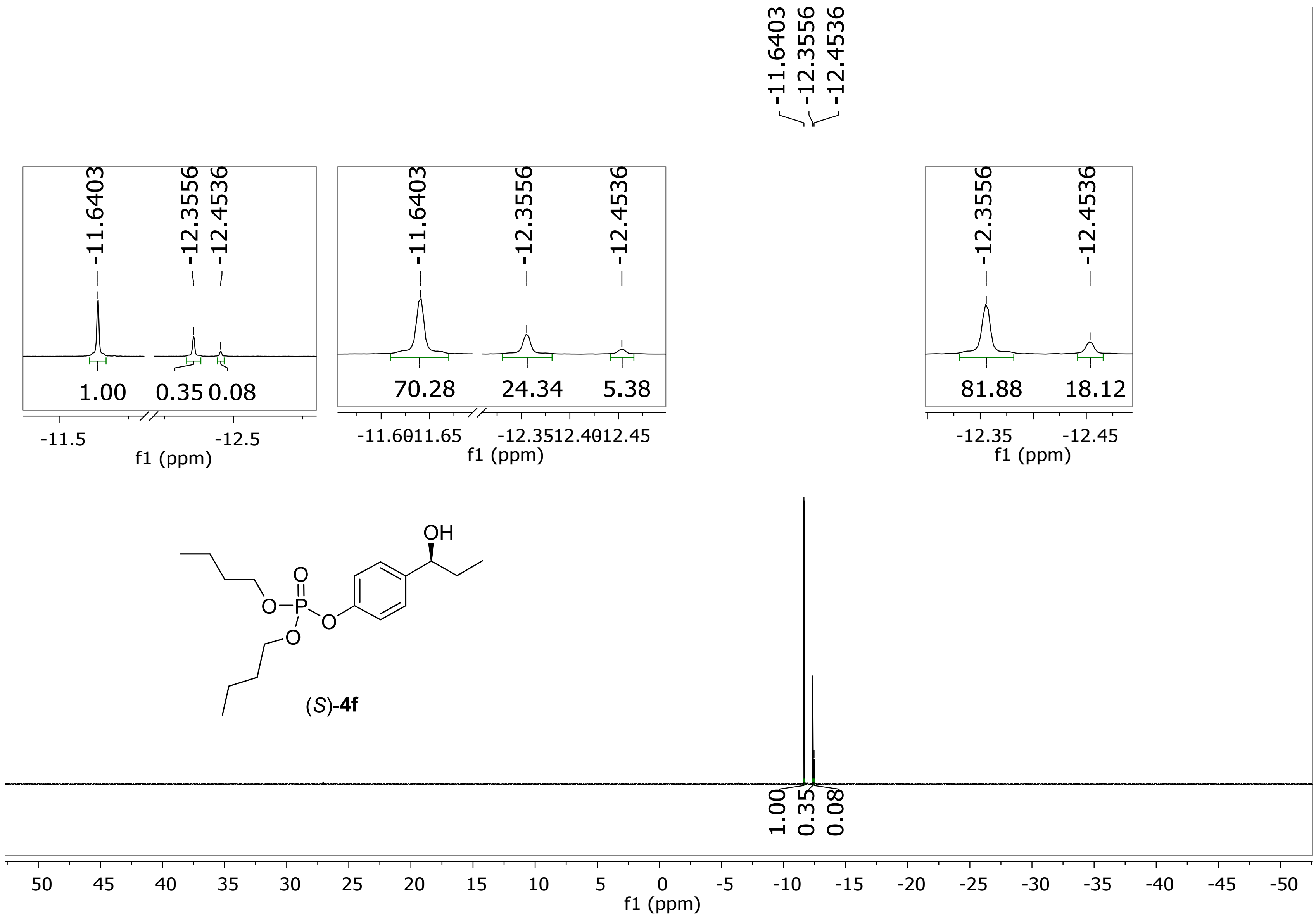

Figure S76. Chiral discrimination of the enantiomerically enriched alcohol **4f** by $^{31}P\{^{1}H\}$ NMR (162 MHz, $CDCl_3$) in cinchonidine

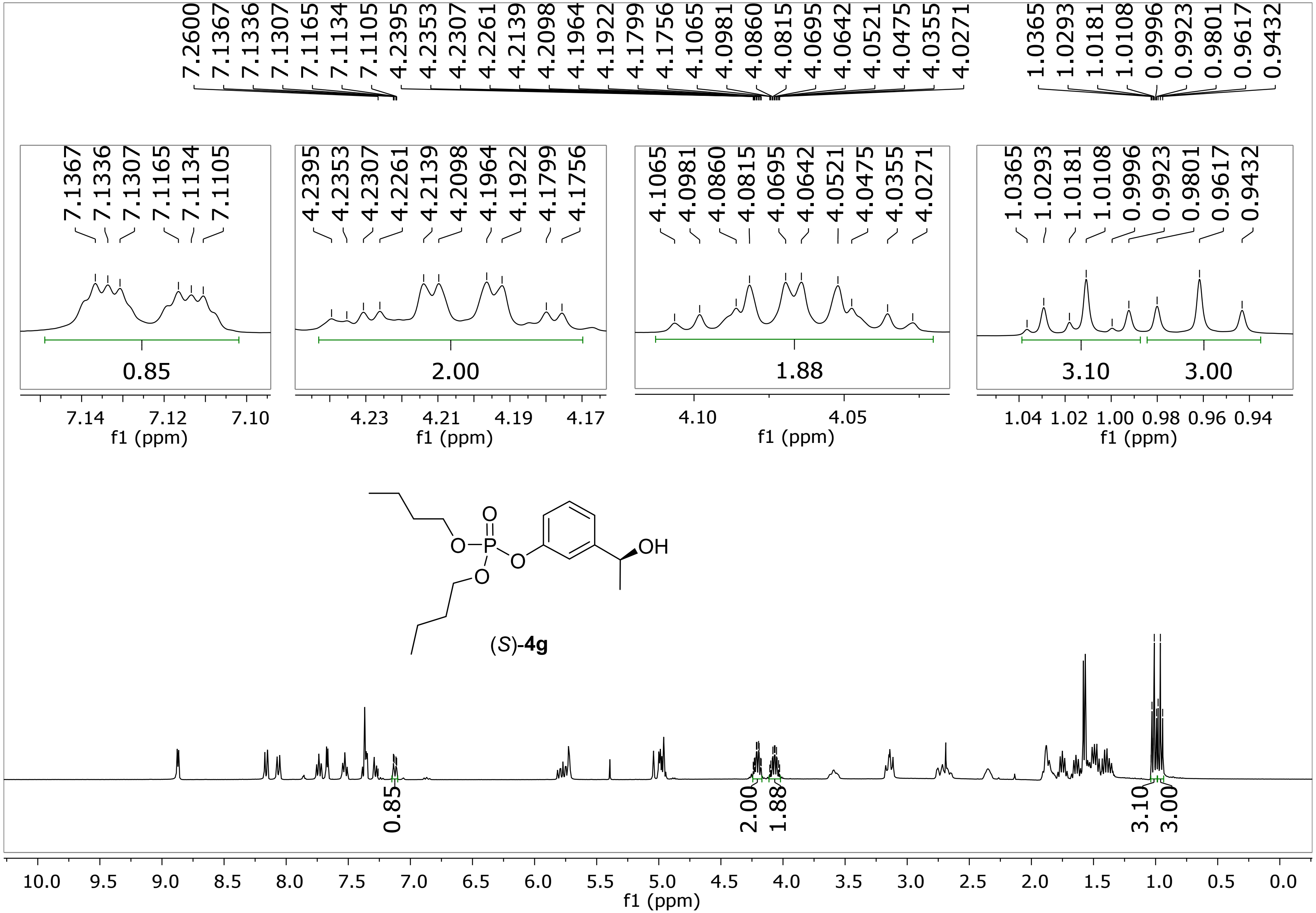

Figure S77. Chiral discrimination of the enantiomerically enriched alcohol **4g** by ^{1}H NMR (400 MHz, $CDCl_3$) in cinchonidine

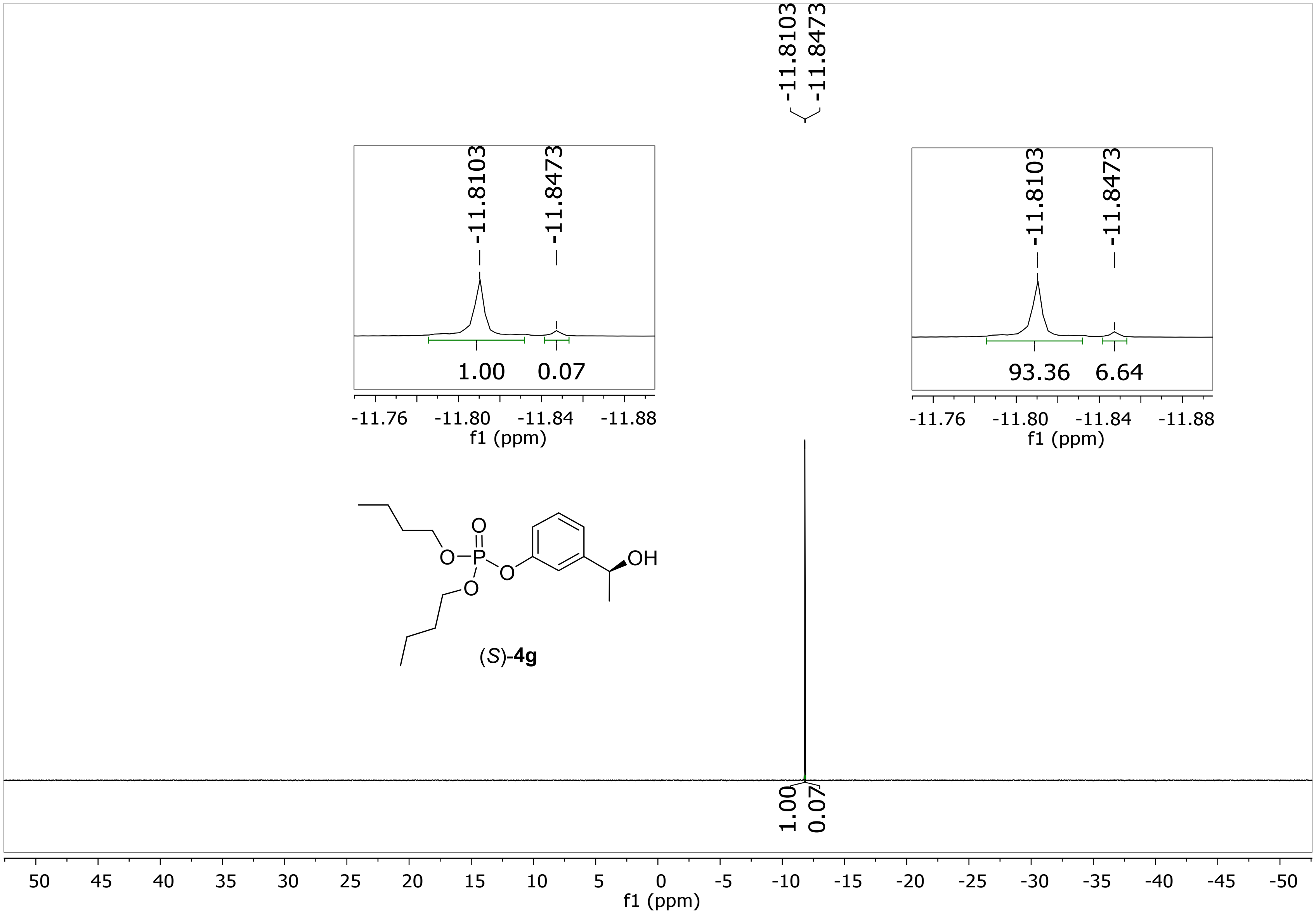

Figure S78. Chiral discrimination of the enantiomerically enriched alcohol **4g** by $^{31}P\{^{1}H\}$ NMR (162 MHz, $CDCl_3$) in cinchonidine

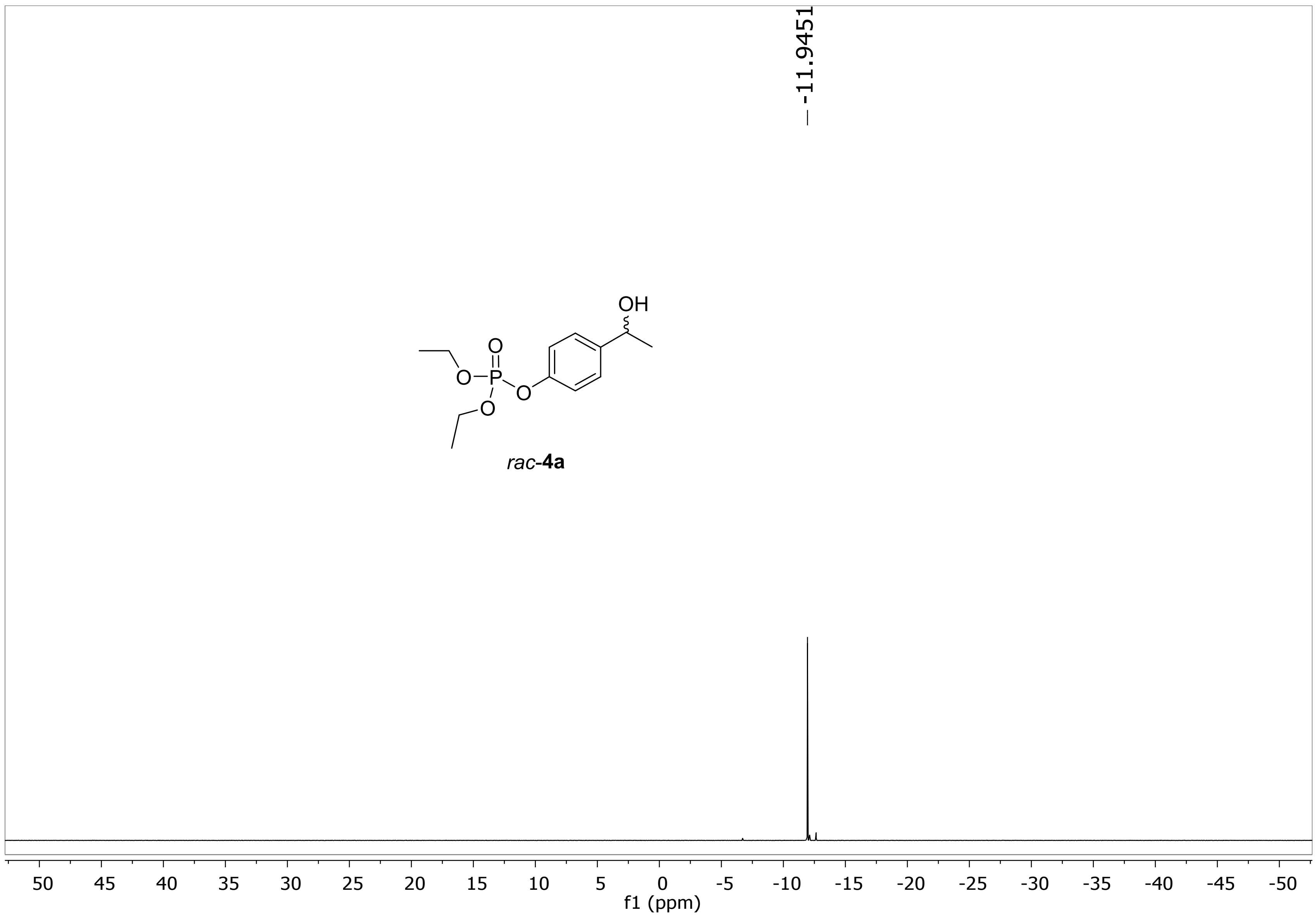

Figure S79. Chiral discrimination spectrum of racemic alcohol **4a** by ^{1}H NMR (400 MHz, $CDCl_3$) in cinchonidine (Reaction at 40 ºC)

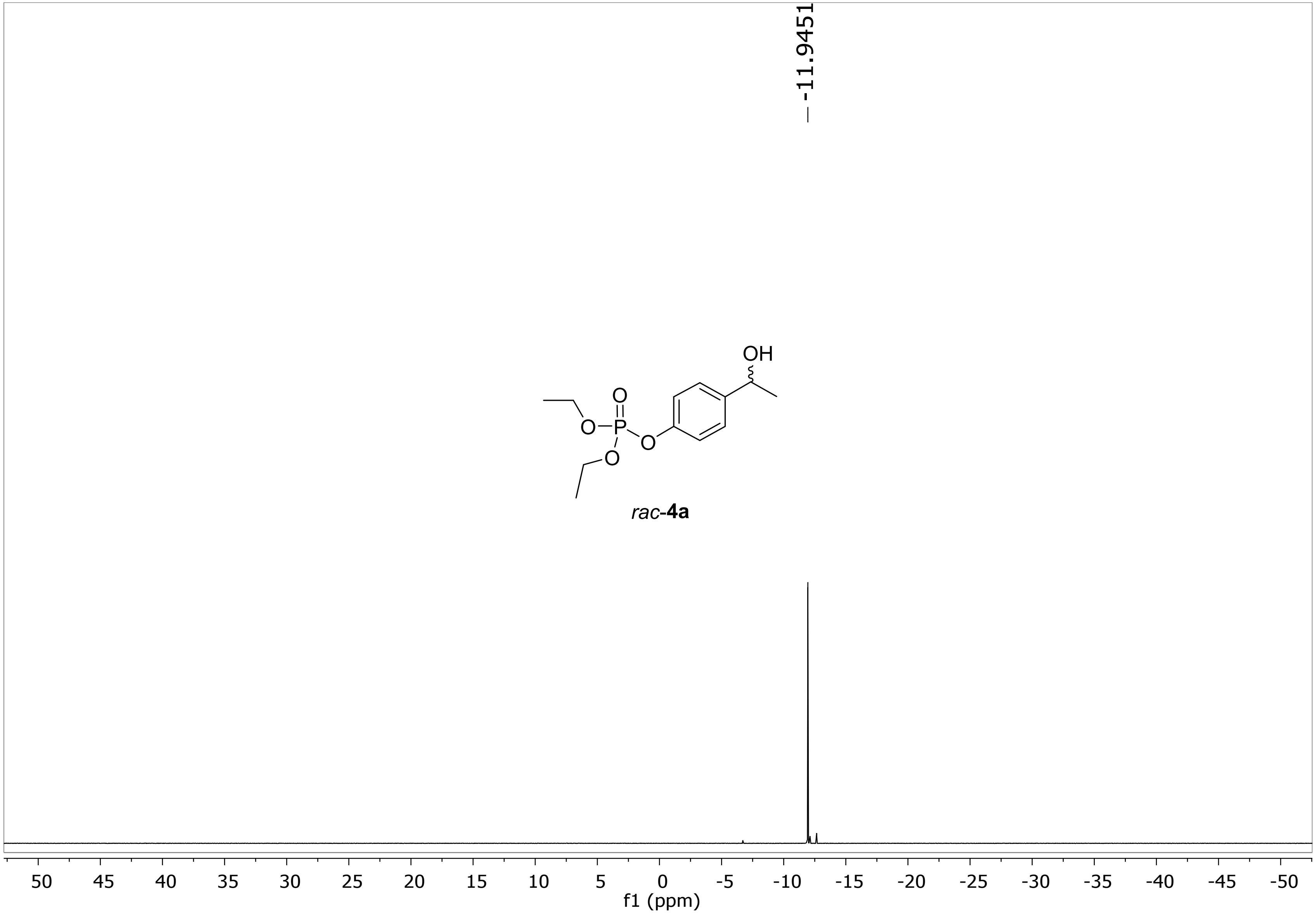

Figure S80. Chiral discrimination spectrum of racemic alcohol **4a** by $^{31}P\{^1H\}$ NMR (162 MHz, $CDCl_3$) in cinchonidine (Reaction at 40 ºC)

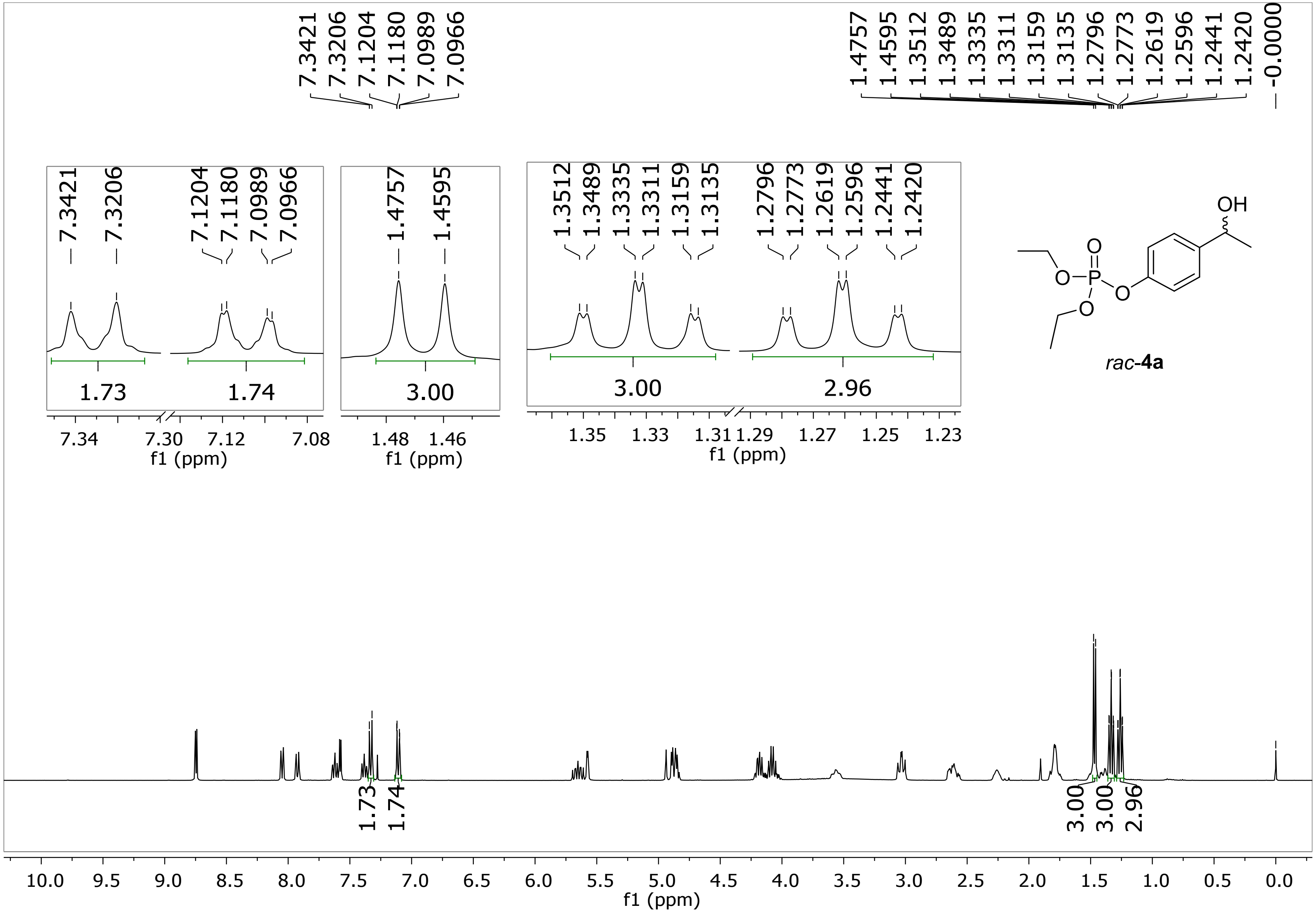

Figure S81. Chiral discrimination spectrum of racemic alcohol **4a** by ^{1}H NMR (400 MHz, $CDCl_3$) in BINOL

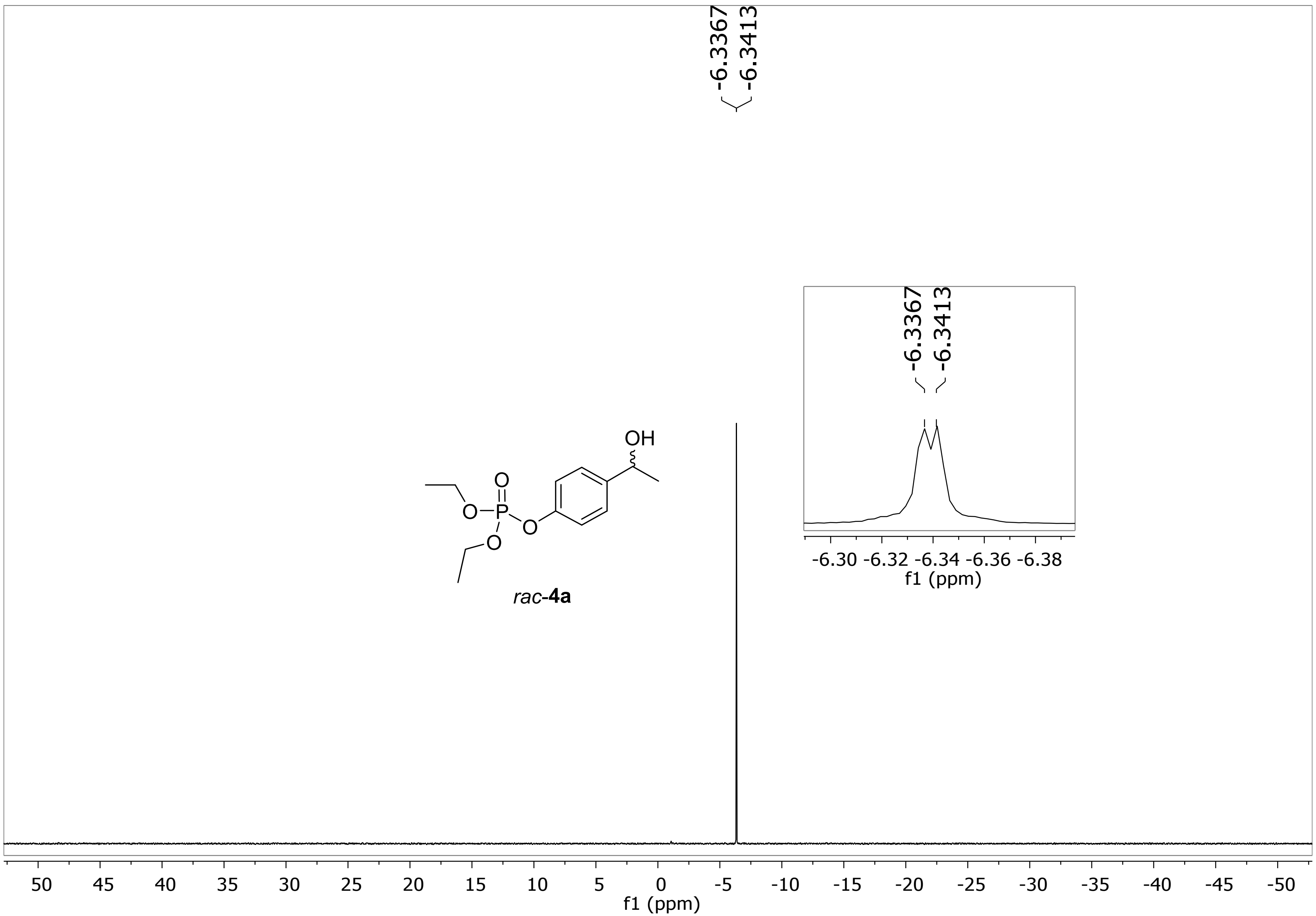

Figure S82. Chiral discrimination spectrum of racemic alcohol **4a** by $^{31}P\{^{1}H\}$ NMR (162 MHz, $CDCl_3$) in BINOL

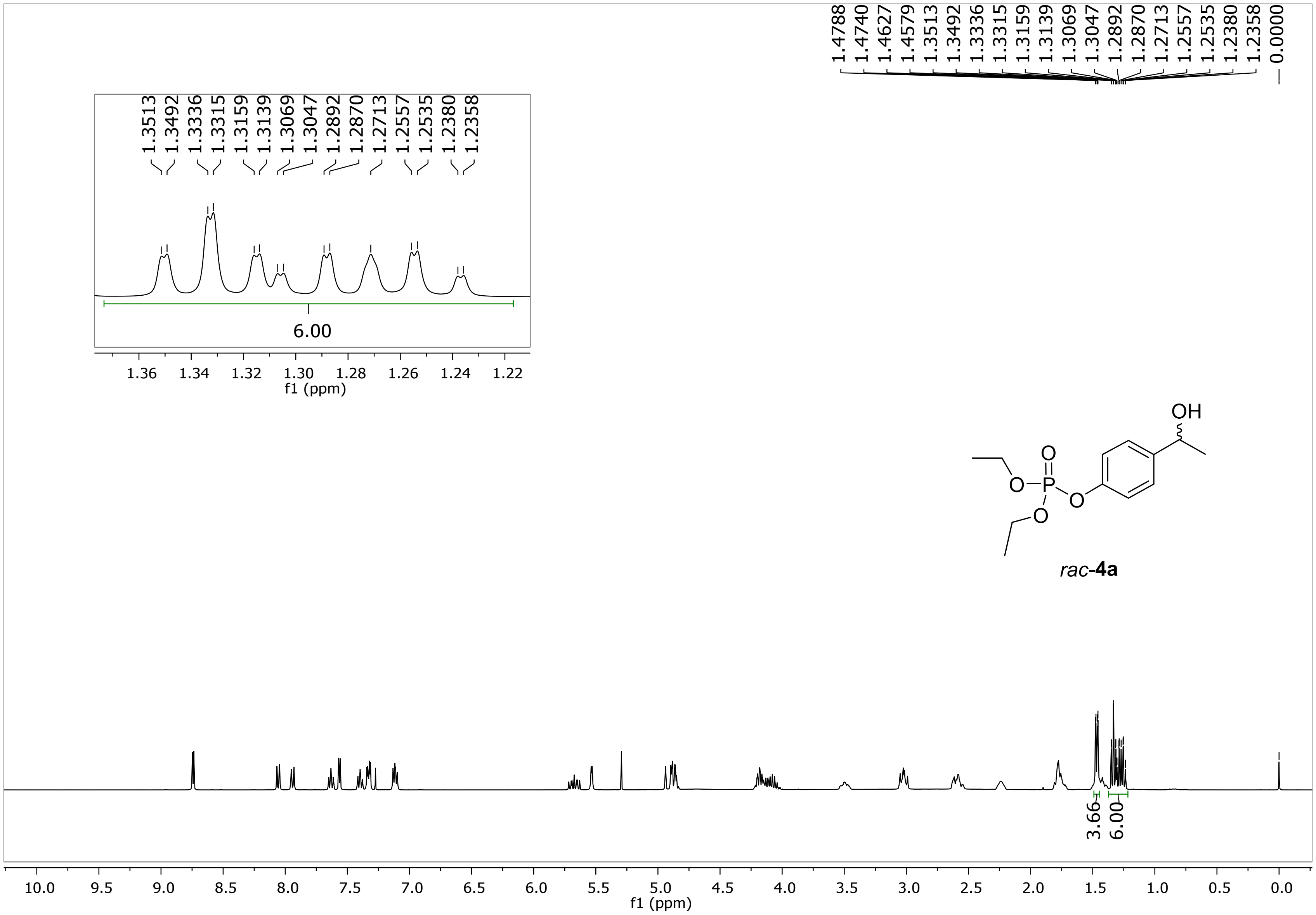

Figure S83. Chiral discrimination spectrum of racemic alcohol **4a** by ^{1}H NMR (400 MHz, $CDCl_3$) in cinchonidine (2.0 equiv.)

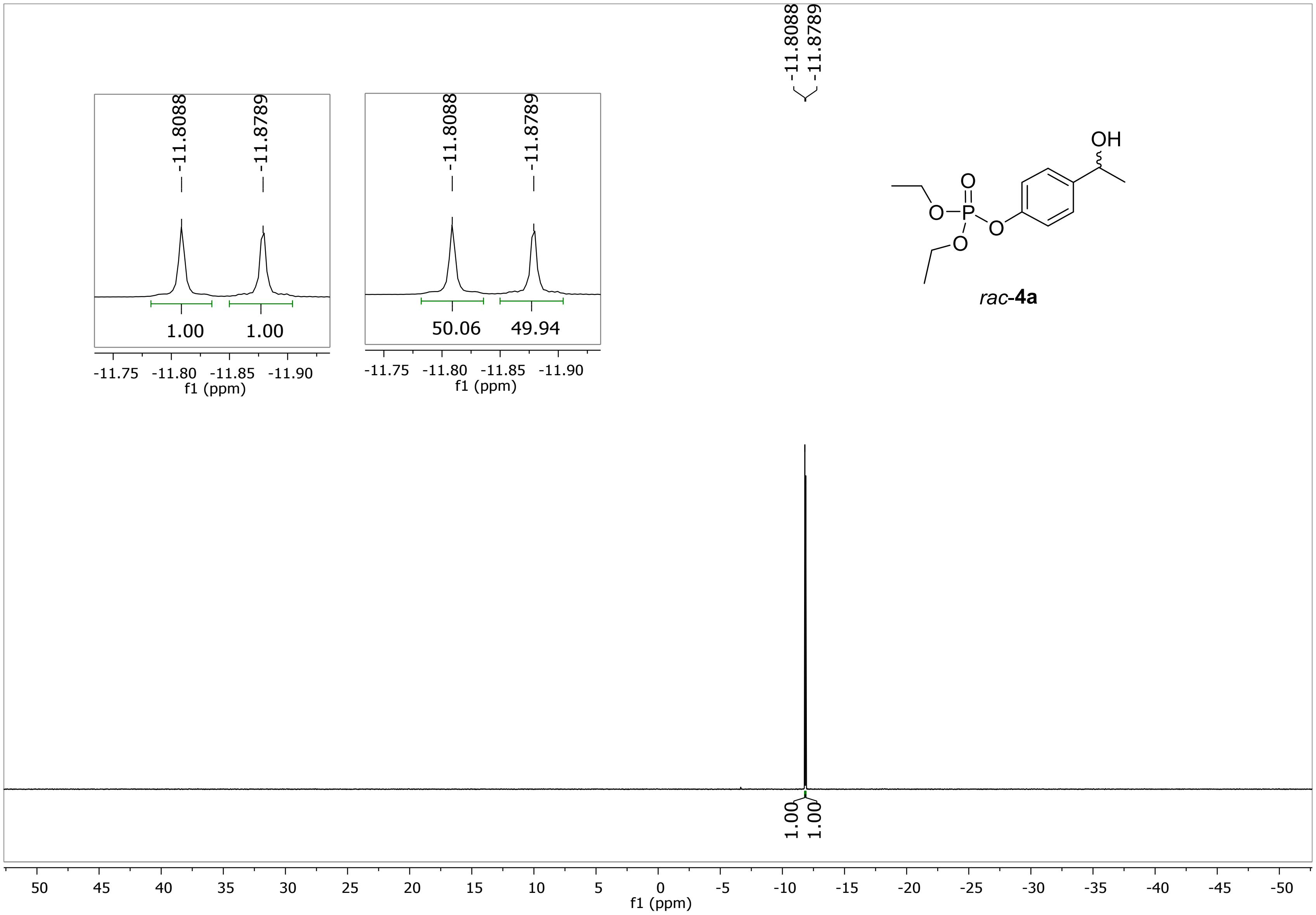

Figure S84. Chiral discrimination spectrum of racemic alcohol **4a** by $^{31}P\{^{1}H\}$ NMR (162 MHz, $CDCl_3$) in cinchonidine (2.0 equiv.)

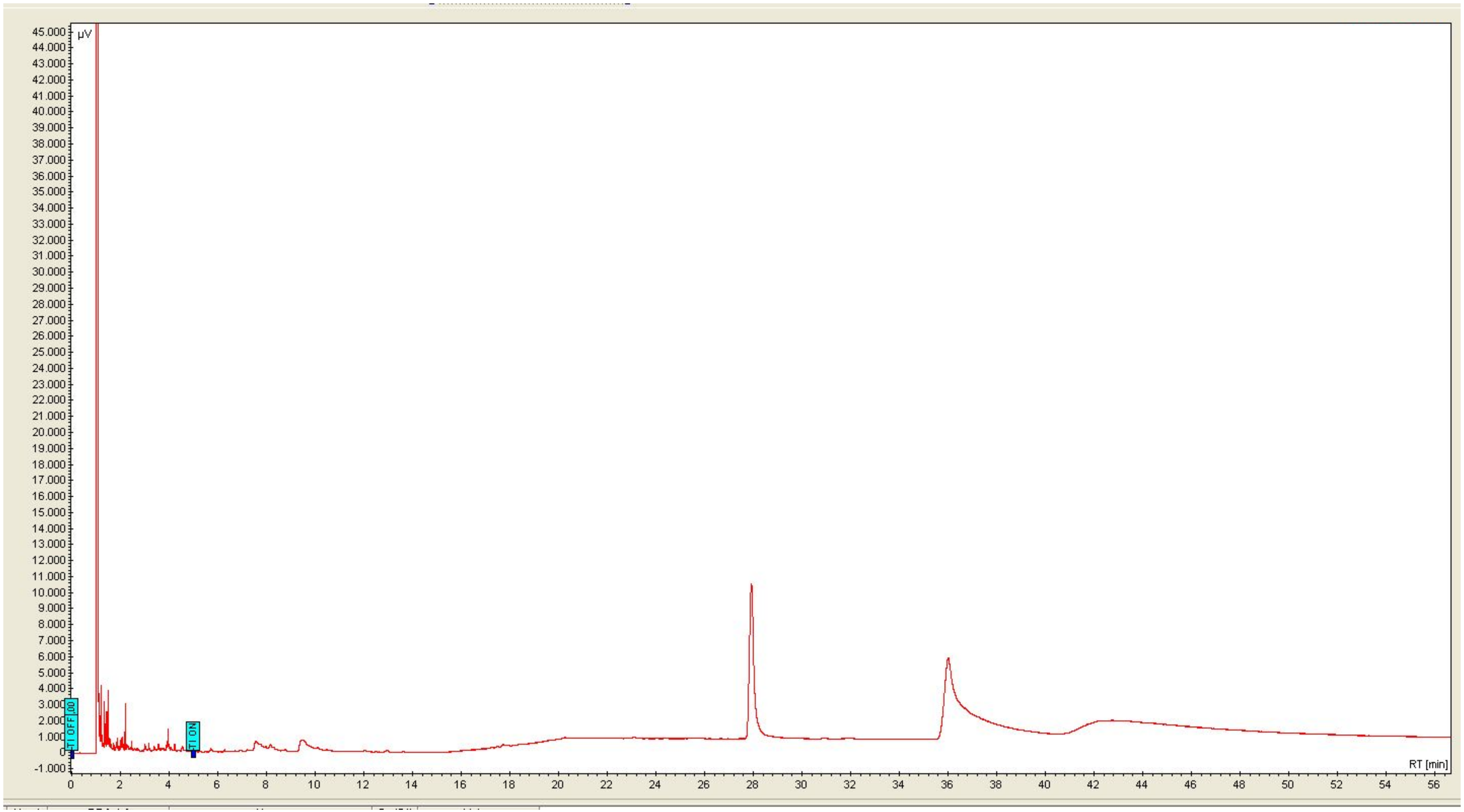

Figure S85. Chiral GC-FID of compound *rac*-**4d**

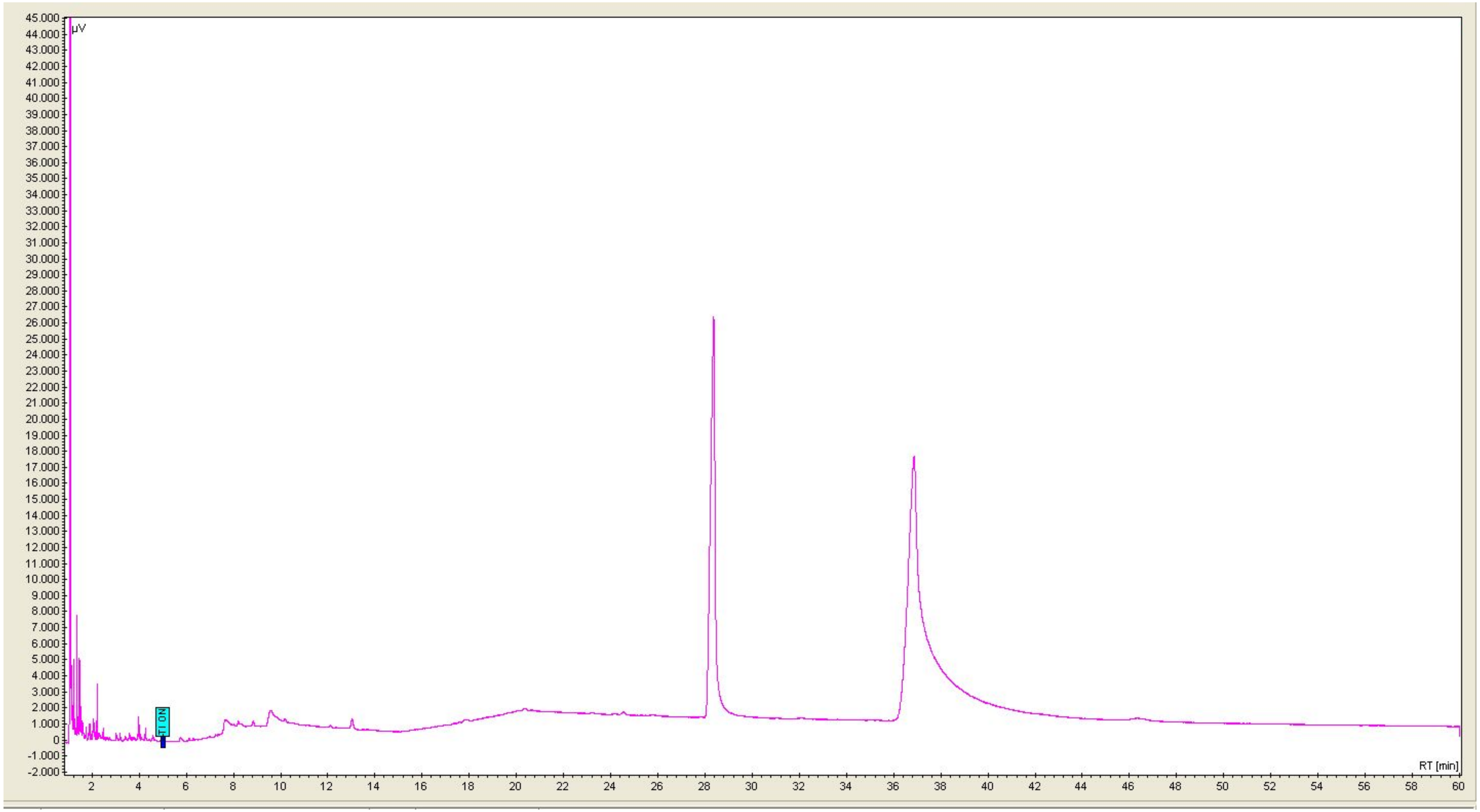

Figure S86. Chiral GC-FID of compound *rac*-**4h**

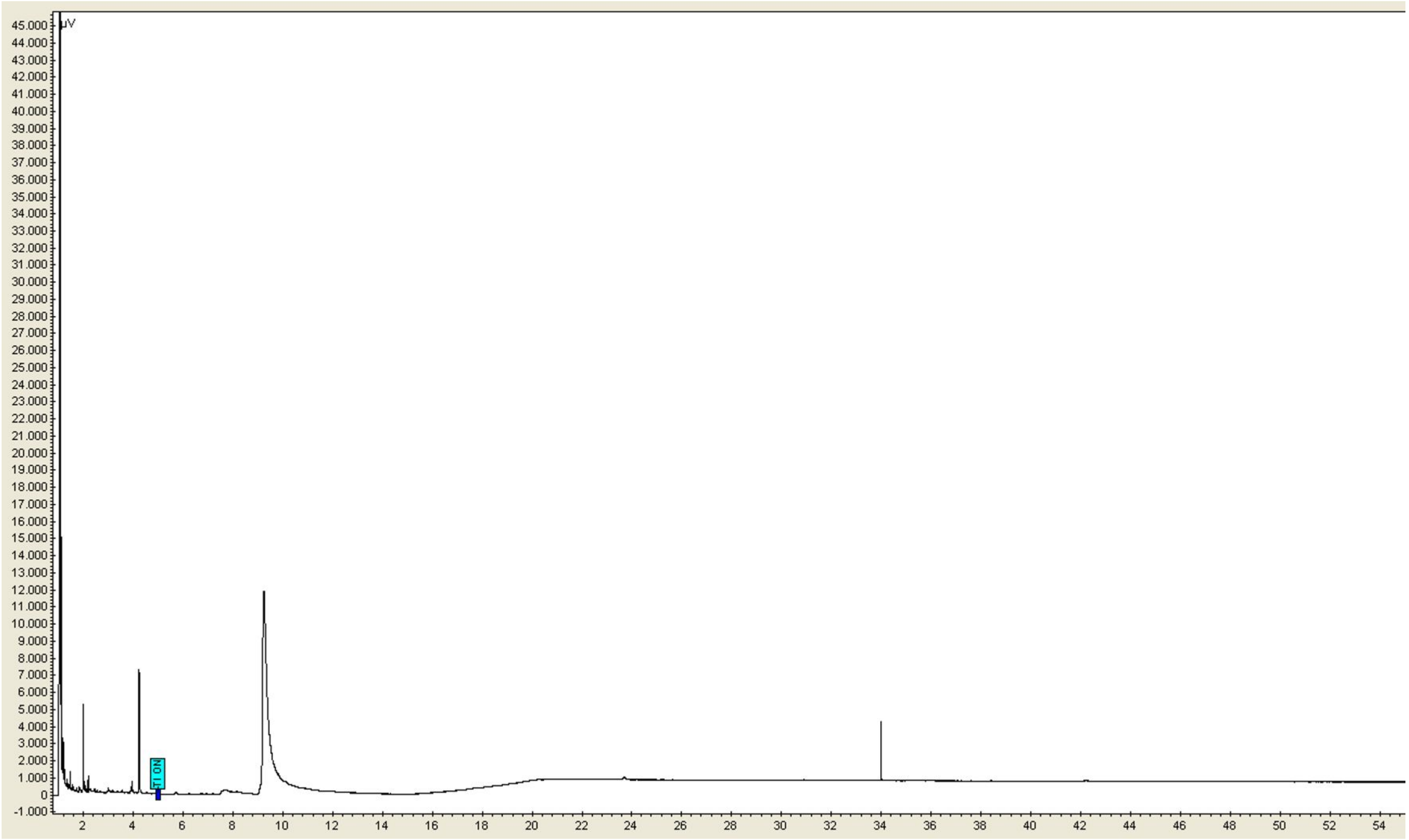

Figure S87. Chiral GC-FID of compound *rac*-**4a**

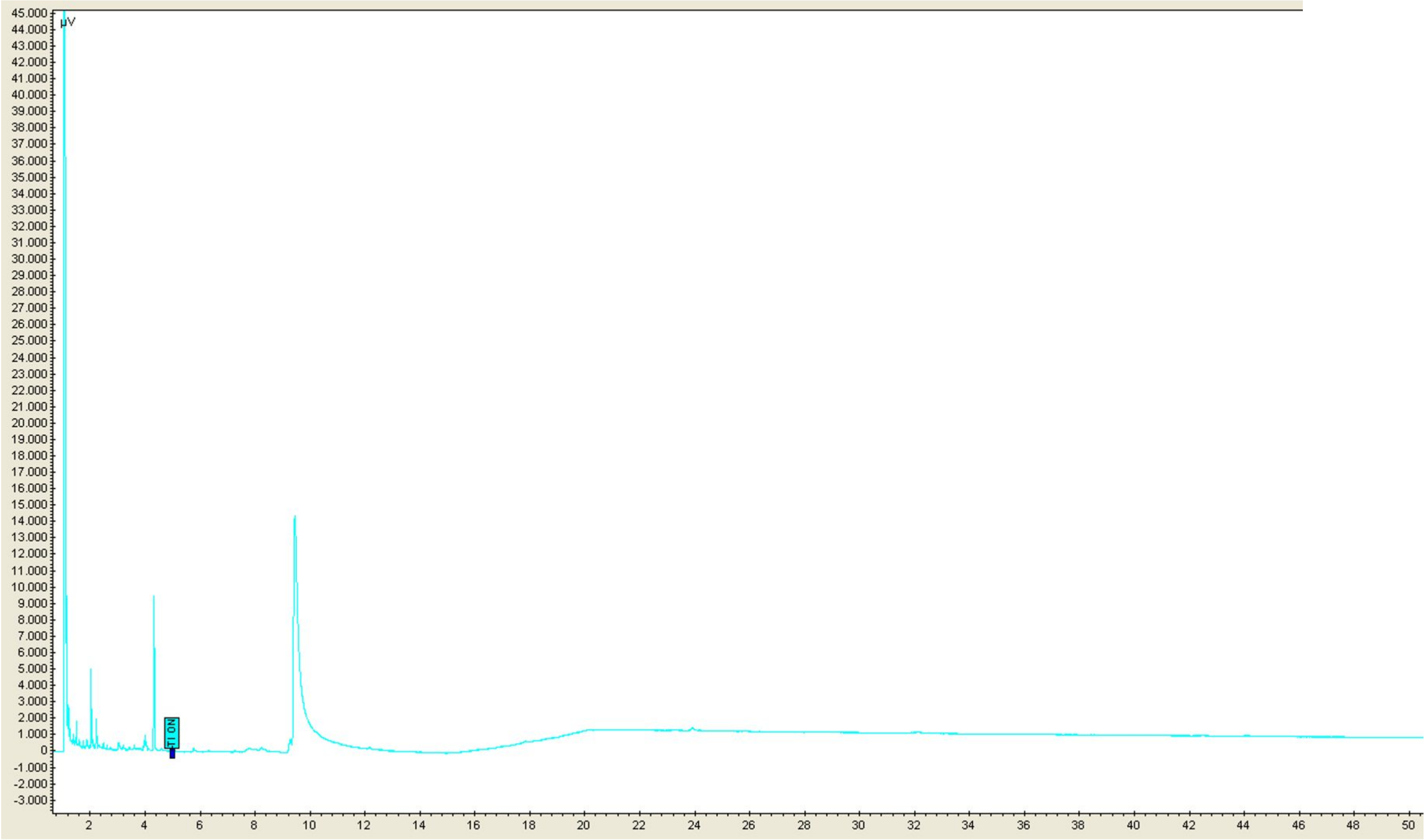

Figure S88. Chiral GC-FID of compound *rac*-**4b**
